# Total Synthesis of JBIR-141 and Its Analogues: Potent Inhibitors of Forkhead Box O3 (FOXO3)

**DOI:** 10.1021/acs.orglett.6c02149

**Published:** 2026-07-18

**Authors:** Hidetoshi Ban, Kotaro Yasoshima, Kosuke Ohsawa, Madoka Suzuki, Noritaka Kagaya, Teppei Kawahara, Hiroyuki Yamakoshi, Masahito Yoshida, Kazuo Shin-ya, Takayuki Doi

**Affiliations:** † Graduate School of Pharmaceutical Sciences, 13101 Tohoku University, 6-3 Aza-aoba, Aramaki, Sendai 980-8578, Japan; ‡ Graduate School of Pharmacy and Pharmaceutical Sciences, Juntendo University, 6-8-1 Hinode, Urayasu 279-0013, Japan; § Department of Chemistry, Institute of Pure and Applied Sciences, University of Tsukuba, 1-1-1 Tennodai, Tsukuba, Ibaraki 305-8571, Japan; ∥ Technology Research Association for Next Generation Natural Products Chemistry, 2-4-7 Aomi, Koto-ku, Tokyo 135-0064, Japan; ⊥ 13508 National Institute of Advanced Industrial Science and Technology (AIST), 2-4-7 Aomi, Koto-ku, Tokyo 135-0064, Japan; # Japan Biological Informatics Consortium (JBIC), 2-4-7 Aomi, Koto-ku, Tokyo 135-0064, Japan

## Abstract

The total synthesis of JBIR-141 and its analogues was achieved through a convergent assembly of three fragments. This synthesis successfully addressed several challenges arising from a highly acid-sensitive oxazoline, a reactive nitrosohydroxyamino group, and an epimerization-prone tetramic acid. The developed synthetic route enabled access to 25-methyl/17-methyl derivatives as potent/stabilized FOXO3 inhibitors. This study provides a robust platform for structure–activity relationship studies and future target identification in chronic myelogenous leukemia therapeutics.

## Graphical abstract

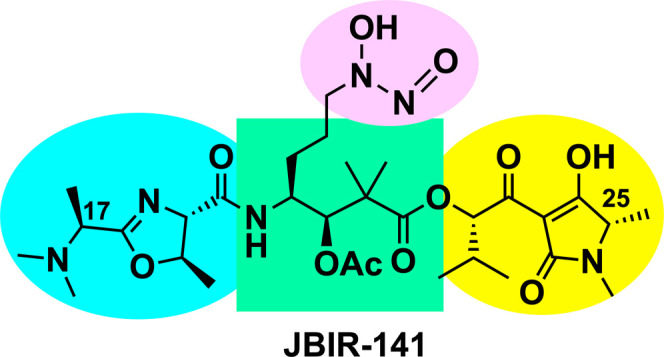

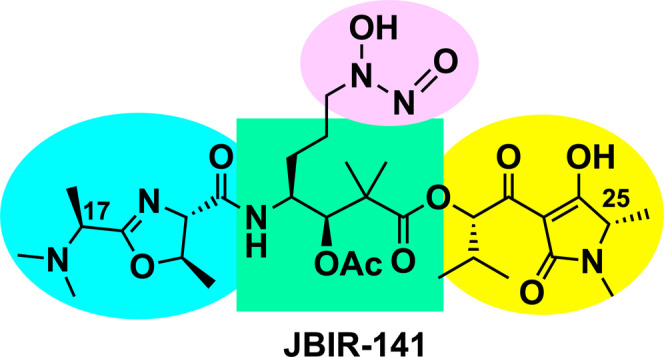

JBIR-141 (**1a**) and JBIR-142 (**2**), isolated from *Streptomyces* sp. 4587H4S, exhibit intriguing biological activities and possess a distinctive molecular architecture composed of three structural fragments: 1-(1-(dimethylamino)­ethyl)-5-methyl-4,5-dihydrooxazole-4-carboxylic acid (fragment A), 3-acetoxy-4-amino-7-(hydroxy­(nitroso)­amino)-2,2-dimethylheptanoic acid (fragment B), and 3-(2-hydroxy-3-methylbutanoyl)-1,5-dimethyltetramic acid (fragment C) (Figure 1).^1^ The *N*-nitroso-*N*-hydroxyamino group found in fragment B is an emerging structural motif of significant interest. Recently, several peptide-based siderophores containing one or two graminine (*N*-nitroso-*N*-hydroxyornithine) residues have been reported, namely, gramibactin, megapolibactins, plantaribactin, gladiobactin, trinickiabactin, tistrellabactins, and pandorachelin.^2–4^ Despite this structural similarity, **1a** and **2** exhibit distinct biological activity; they suppress FOXO3 expression with IC_50_ values of 23 and 166 nM, respectively. Because FOXO3 functions as a stemness factor in chronic myelogenous leukemia (CML) stem cells, its inhibition is expected to regulate their self-renewal capacity, suggesting that JBIR-141 (**1a**) could serve as a promising lead for CML therapeutics.^5,6^

**Figure 1:**
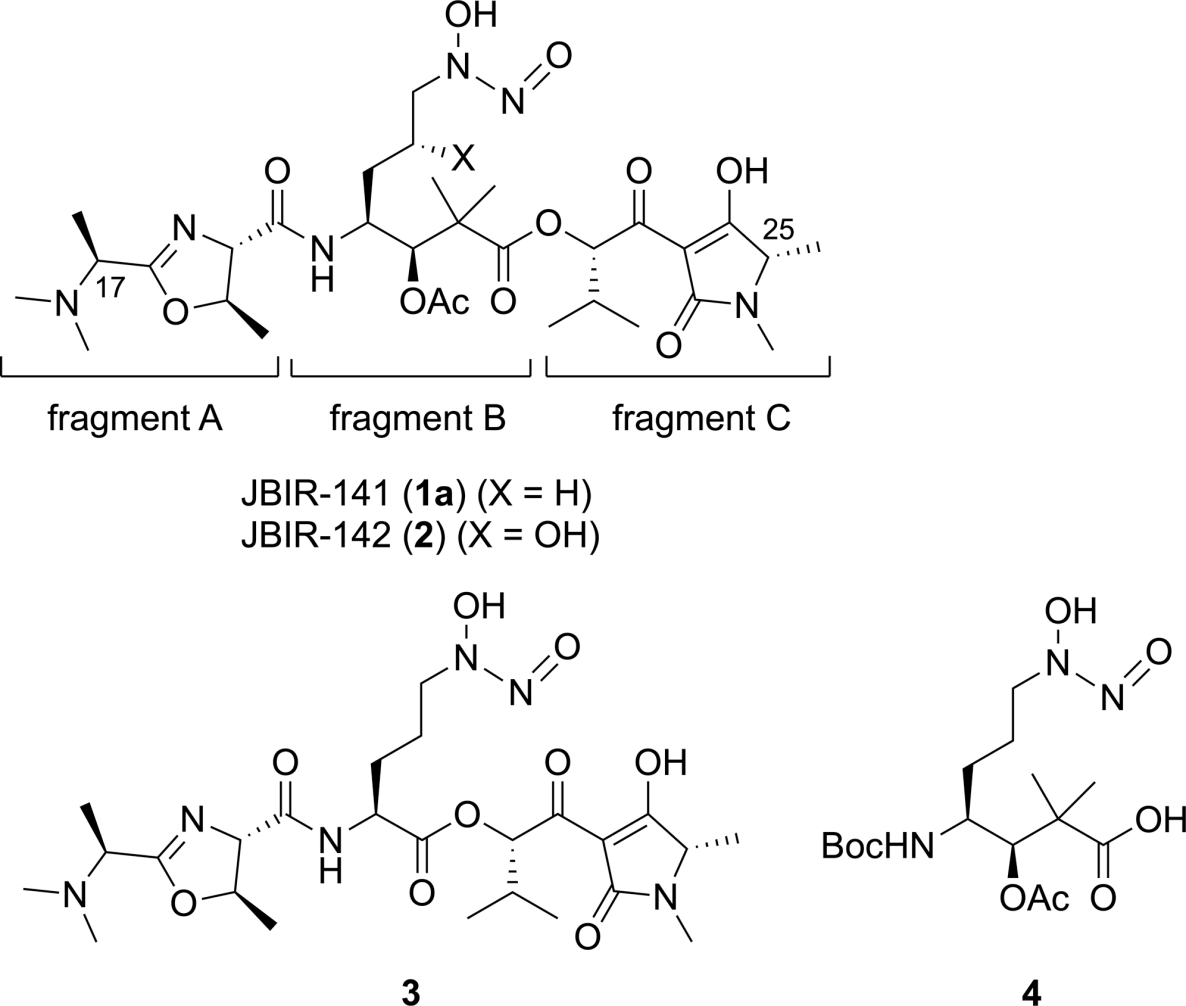
Structures of JBIR-141 (**1a**), JBIR-142 (**2**), reported analogue **3**, and fragment B **4**.

However, JBIR-141 (**1a**) is highly labile: hydrolysis of the oxazoline moiety readily occurs, leading to diminished biological activity.^1^ Wittmann et al. reported the synthesis of compound **3**, lacking the isobutyl acetate linker in fragment B, and they demonstrated that neither **3** nor its synthetic intermediates exhibited cytotoxicity against cancer cell lines (L929 and KB3.1) or antimicrobial activity against a panel of 12 microorganisms.^7^ These findings suggest that the whole framework of JBIR-141 is essential for its potent biological activity. Herein, we report the total synthesis of JBIR-141 (**1a**) and four analogues **1b**, **1c**, **5a**, and **5b**. Notably, the 25-methyl derivative **1b** exhibited enhanced FOXO3 inhibitory activity and cytotoxicity relative to **1a**, whereas replacement of the oxazoline moiety with an oxazole significantly reduced the activity.

We previously reported the synthesis of fragment B **4**;^8^ nevertheless, several synthetic challenges remained in assembling fragments A–C en route to the total synthesis of **1a**: (i) the oxazoline moiety is highly acid-labile, as evidenced by ring-opening during concentration of purified **1a** obtained by preparative reversed-phase HPLC in the presence of 0.1% formic acid;^1^ (ii) fragment B readily undergoes lactamization upon activation at the C-terminus; (iii) the *N-*nitroso-*N-*hydroxyamino group is highly susceptible to nucleophilic substitution and acid-mediated decomposition; and (iv) epimerization at the 25 position readily occurs under acidic conditions, requiring the avoidance of acidic media after the formation of fragment C. Furthermore, because 3-acyltetramic acids exist as multiple tautomers, detection of epimerization by spectroscopic methods is challenging.^9^ We successfully addressed these issues and achieved the total synthesis of JBIR-141 (**1a**).

As an initial strategy, we explored the synthesis of the oxazole analogue **5**, expecting the oxazole ring to serve as an acid-stable bioisostere of the labile oxazoline moiety in **1a** (Figure 2).^10^ Fragments A–C were represented by compounds **7**, **8**, and **9**, respectively. As 3-acyltetramic acid readily undergoes epimerization under both acidic and basic conditions, we planned to use a cyclization precursor for fragment assembly and subsequent late-stage formation of the tetramic acid. As a precursor, 2-naphthylmethyl enol ether **9** was selected because the Nap group can be cleaved under several different conditions, such as oxidative cleavage, HF/pyridine treatment, and hydrogenolysis. Coupling of **8** and **9**, followed by the condensation with **7**, was expected to afford **6**.^11^ Removal of the Nap and Cbz groups in **6**, *N-*nitrosation, Dieckmann condensation to form the tetramic acid,^12^ and hydrogenolysis of the benzyl ether would lead to **5**.

**Figure 2:**
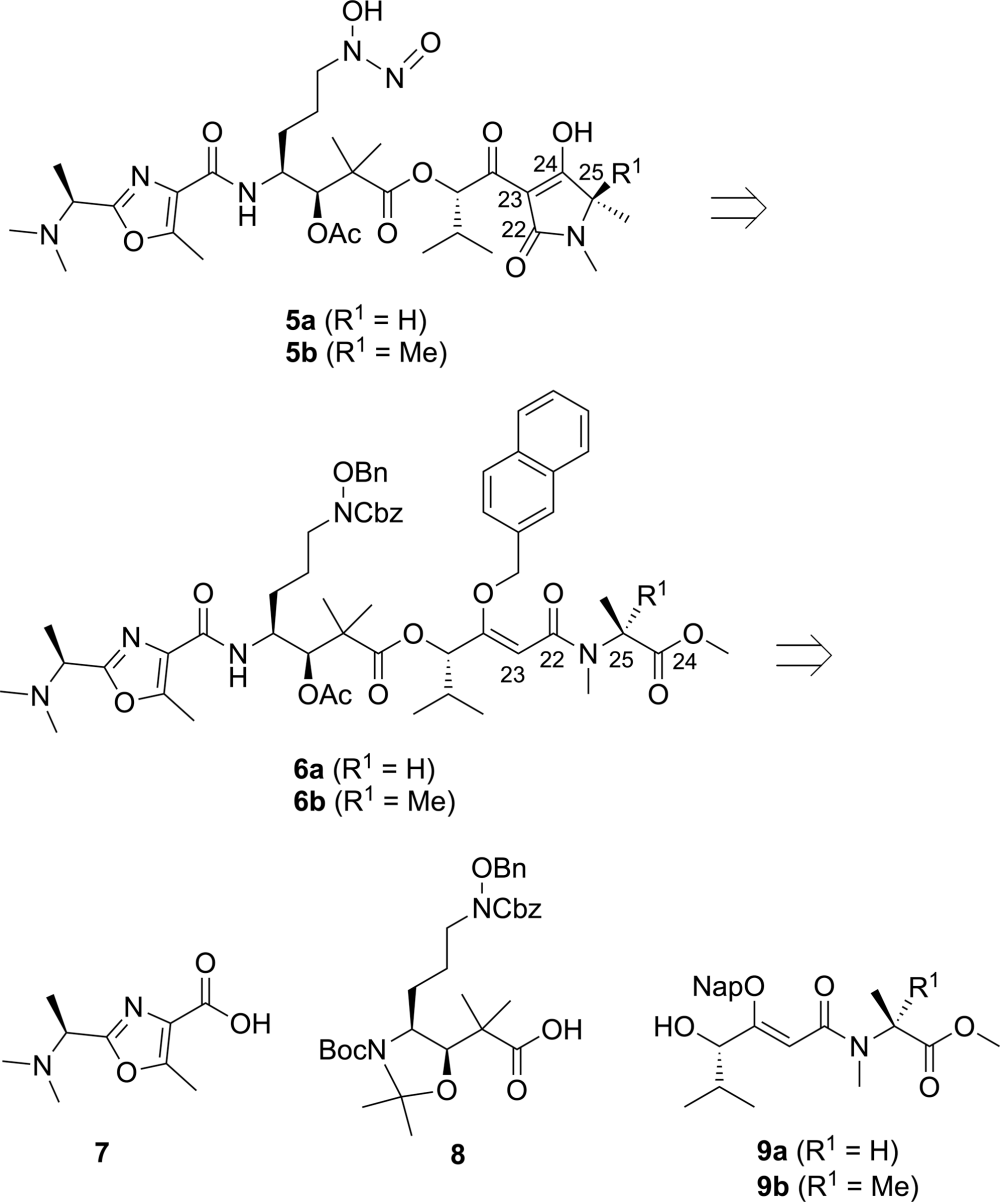
Retrosynthesis of oxazole analogue **5**.

We selected *N-*methylalanate **9a** (R^1^ = H) and *N-*methyl-2-aminoisobutyrate derivative **9b** (R^1^ = Me) as precursors for tetramic acid fragment C. The latter does not have an issue of epimerization at the 25 position, allowing straightforward product analysis. The Mitsunobu reaction of β-ketoester **10** ^13^ with 2-naphthylmethanol afforded the corresponding enol ether, and Pd-catalyzed allyl ester deprotection provided acid **11** (Scheme 1). Condensation of **11** with *N-*methylamino acid methyl esters **12a** and **12b** using HATU, followed by removal of the TBS group furnished alcohols **9a** and **9b**. Fragment B **8**, readily prepared from a synthetic intermediate en route to compound **4**,^8,13^ was coupled with secondary alcohols **9a** and **9b** using TCFH/pyridine/DMAP/MeCN in the presence of MS 3A under heating at 50 °C to give **13a** and **13b** in yields of 78% and 73%, respectively. Importantly, γ-lactam formation was avoided because the 4-amino group of **8** was protected with a Boc group and an acetonide, preventing intramolecular attack on the activated ester. In the absence of MS 3A, partial hydrolysis of the enol ether occurred, likely via 1,4-addition of water followed by elimination of NapOH. Removal of these protecting groups with TMSOTf yielded the desired free amino alcohol. During deprotection, use of 2,6-lutidine^14^ led to decomposition of the enol ether, whereas adding potassium carbonate suppressed this pathway and promoted imine exchange with methanolamine. The *N-*nitroso-*N-*benzyloxyamino function proved unstable under these conditions, necessitating late-stage nitrosation. Coupling of the resulting amino alcohol with acid **7** using TCFH/DIEA/MeCN and subsequent acetylation of the secondary alcohol at 80 °C afforded **6a** and **6b** in overall yields of 54% and 52%.

**Scheme 1:**
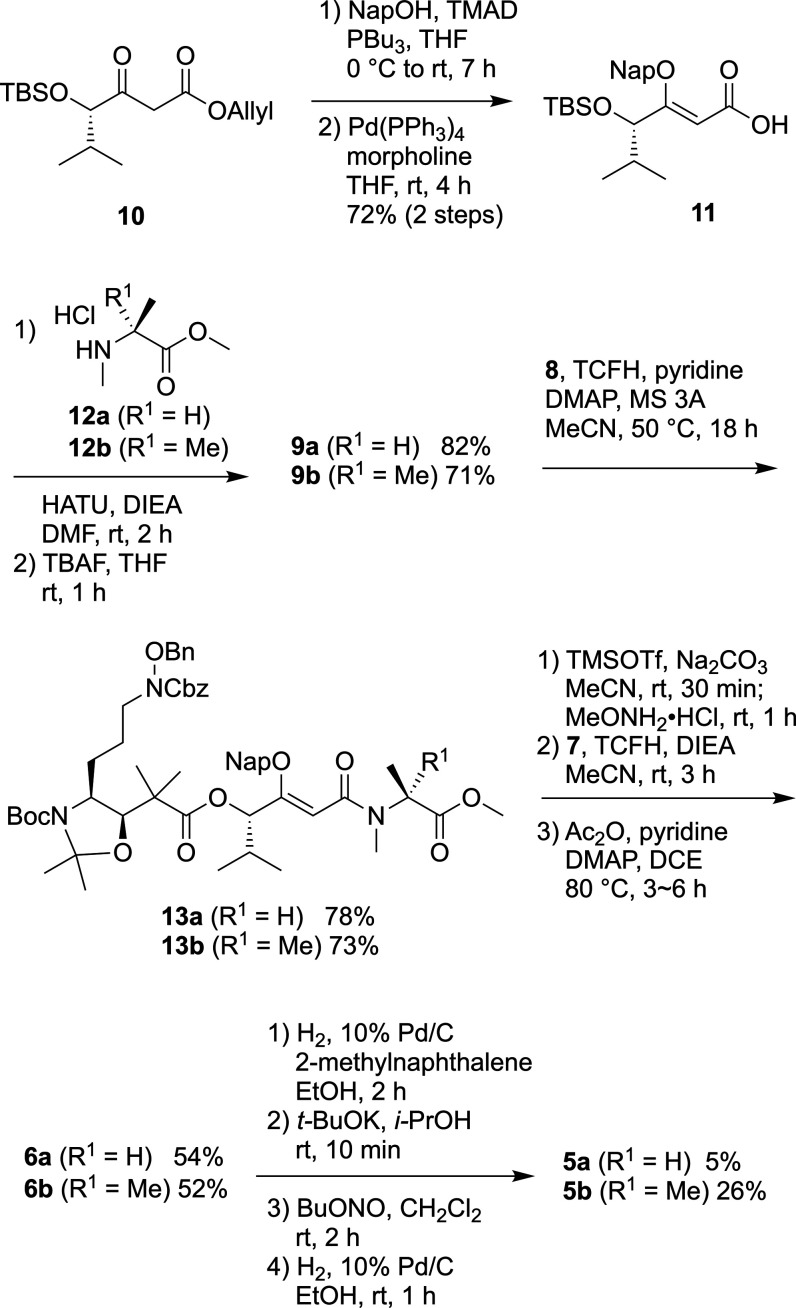
Synthesis of Oxazole Analogues **5a** and **5b**

Selective cleavage of the Nap group in **6** using HF/pyridine, successful in simpler substrates,^15^ was incomplete. Alternatively, hydrogenolysis of **6a** and **6b** in the presence of 2-methylnaphthalene removed the Cbz and Nap groups while preserving the N–O and benzyl ether functionalities. In the absence of 2-methylnaphthalene, premature cleavage of the N–O bond occurred, suggesting that π-stacking of 2-methylnaphthalene on Pd attenuates undesired N–O bond cleavage.^16^

The Dieckmann condensation was performed according to the procedure reported by Ley et al.^12^ Initial attempts using *t*-BuOK in *t*-BuOH resulted in a sluggish reaction. While the use of EtOH promoted undesired ethoxy substitution at the 24 position of the product, switching the solvent to *i*-PrOH successfully afforded the desired product. Subsequent nitrosation with butyl nitrite under conditions similar to those used for the synthesis of **4**,^8,17^ followed by hydrogenolysis of the benzyl ether, furnished the desired oxazole analogues **5a** and **5b**. Although the overall yields are low (5% for **5a** and 26% for **5b**), most of the loss is due to chromatographic isolation and low conversion of the initial hydrogenolysis for **6a** (not optimized). By comparing the experimental results of the synthesis of **5a** and **5b**, it was clearly confirmed that no epimerization occurred at the 25 position during and after the formation of the tetramic acid using UPLC analysis. Conducting nitrosation prior to tetramic acid formation gave an α-hydroxyimino-β-keto ester that did not undergo subsequent Dieckmann condensation.

The total synthesis of JBIR-141 (**1a**) and its oxazoline-containing analogues were accomplished as outlined in Scheme 2. The amino alcohols derived from **13a** and **13b** were coupled with **14a** ^13^ using COMU/DIEA/MeCN. Whereas acetylation of the oxazole derivatives required heating to 80 °C, such conditions decomposed the oxazoline ring of **15**. Fortunately, acetylation of the oxazoline derivatives proceeded smoothly at room temperature, enabling preservation of the oxazoline ring. Subsequent transformations similar to those used in the synthesis of **5** afforded JBIR-141 (**1a**) and its 25-methyl derivative **1b**, respectively. Purification of these products initially required reversed-phase silica gel chromatography, with subsequent lyophilization of eluents because evaporation of eluents resulted in decomposition. The NMR spectra and specific rotation of lyophilized **1a** ([α]_D_ +54 (*c* 0.30, MeOH)), however, differed from those reported for natural JBIR-141 ([α]^23^_D_ + 90 (*c* 0.2, MeOH)).^1^ Remarkably, short passage through normal-phase silica gel chromatography afforded the final product whose specific rotation (**1a**, [α]_D_ + 96 (*c* 0.30, MeOH)), NMR spectra, and UPLC retention time were in good agreement with those of the natural product. It is conceivable that depending on the purification methods, one of them might exist as a zwitterion or a tautomer resulting from the *N-*nitroso-*N-*hydroxyamino group or the 3-acyltetramic acid. Similar phenomena were observed in other synthetic analogues **1** and **5**. Similarly, the 17-methyl JBIR-141 (**1c**) was successfully synthesized from **13a** and **14b**. The 17-methyl analogue **1c** exhibited improved resistance to acid hydrolysis compared to **1a** (Figure S1), validating our design of introducing steric hindrance near the labile oxazoline C=N bond.

**Scheme 2:**
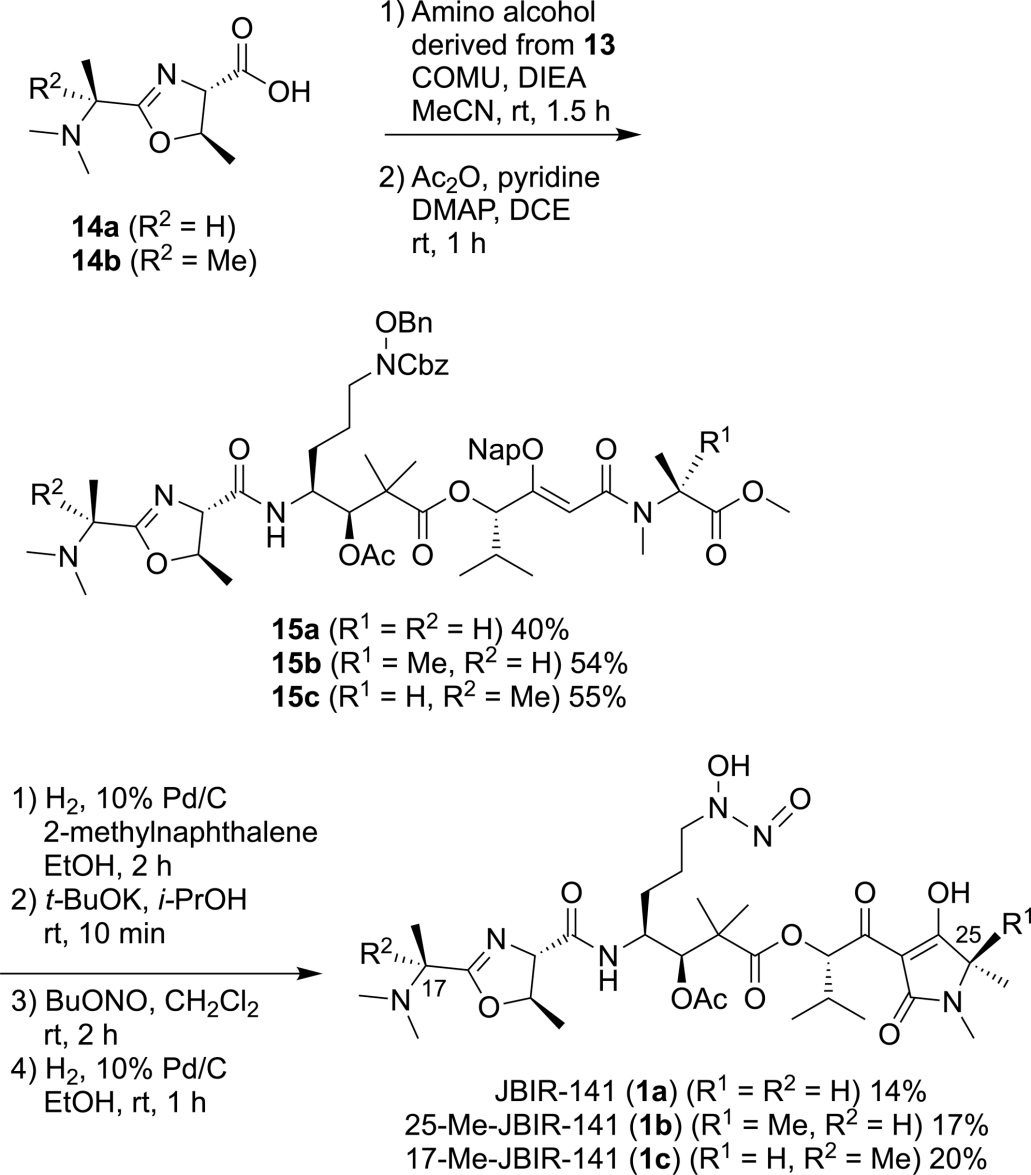
Total Synthesis of JBIR-141 (**1a**) and **25**- and **17**-Methyl Derivatives **1b** and **1c**

Table 1 shows the results of evaluation of FOXO3 inhibition in HEK293 cells and cytotoxicity against Jurkat cells for natural JBIR-141, synthetic **1a**–**1c**, **5a**, and **5b**. The synthetic **1a** exhibited FOXO3 inhibition and cytotoxicity comparable to the natural product (entries 1 and 2). Whereas 25-methyl derivative **1b** surpassed the natural product in cytotoxicity (entry 3), **1a** and **1b** revealed a similar potency in FOXO3 suppression assays. The more acid-stable 17-methyl derivative **1c** shows a slightly reduced, yet sufficiently potent, activity (entry 4). In contrast, the activity of oxazole analogues **5a** and **5b** decreased significantly, indicating that the oxazoline moiety is essential for the potent inhibitory activity (entries 5 and 6).

**Table 1:** FOXO3 Inhibition and Cytotoxicity of Natural JBIR-141, Synthetic **1a**, **1b**, **1c**, **5a**, and **5b**

		IC_50_ (nM)	
Entry	Compound	FOXO3 expression^a^	Cytotoxicity^b^
1	JBIR-141 (natural product)	16 ± 4 (23)^c^	(4.41)^c^
2	**1a**	25 ± 6	6.4 ± 0.4
3	**1b**	25 ± 5	0.9 ± 0.2
4	**1c**	53 ± 11	16.2 ± 0.7
5	**5a**	1200 ± 450	670 ± 140
6	**5b**	450 ± 130	110 ± 5

a Incubation for 24 h (HEK293 cells).

b Incubation for 72 h (Jurkat cells).

c Reported in ref 1.

In summary, we have accomplished the total synthesis of JBIR-141 (**1a**), leading to the identification of a more-potent 25-methyl-JBIR-141 (**1b**) and a more acid-stable 17-methyl-JBIR-141 (**1c**). Further studies on structure–activity relationships and the synthesis of molecular probes for target identification are underway in our laboratories.

## Supplementary Material



## Data Availability

The data underlying this study are available in the published article and its Supporting Information.

## Notes

Additional experimental details and ^1^H and ^13^C­{^1^H} NMR spectra of **1**, **5**, **6**, **8**–**11**, **13**, and **15** (PDF)

## Abbreviations

Cbz: benzyloxycarbonyl
COMU: ethyl 2-cyano-2-((dimethyliminio)­(morpholino)­methyloxyimino)­acetate hexafluorophosphate
DCE: dichloroethane
DIEA: diisopropylethylamine
DMAP: 4-(dimethylamino)­pyridine
DMF: *N*,*N*-dimethylformamide
FOXO3: forkhead box O3 (human)
HATU: *O*-(7-aza-1*H*-benzotriazol-1-yl)-*N*,*N*,*N*′,*N*′-tetramethyluronium hexafluorophosphate
IC_50_: half maximal inhibitory concentration
MS: molecular sieves
Nap: 2-naphthylmethyl
TBAF: tetrabutylammonium fluoride
TCFH: *N*,*N*,*N*′,*N*′-tetramethylchloroformamidinium hexafluorophosphate
TMAD: *N*,*N*,*N*′,*N*′-tetramethylazodicarboxamide
UPLC: ultrahigh-performance liquid chromatography
