# Supplementary material for: Total Synthesis of JBIR-141 and Its Analogues: Potent Inhibitors of Forkhead Box O3 (FOXO3)
Source: Org Lett. 2026 Jul 18;28(30):9591–5. doi: 10.1021/acs.orglett.6c02149 (PMC13435308; doi:10.1021/acs.orglett.6c02149)

Supporting Information

Total Synthesis of JBIR-141 and Its Analogs: Potent Inhibitors of Forkhead Box O3 (FOXO3)

Hidetoshi Ban,[†] Kotaro Yasoshima,[†] Kosuke Ohsawa,[†,‡] Madoka Suzuki,[§] Noritaka Kagaya,[¢] Teppei Kawahara,[$] Hiroyuki Yamakoshi,[†] Masahito Yoshida,[§] Kazuo Shin-ya,[¶] and Takayuki Doi*,[†]

[†] Graduate School of Pharmaceutical Sciences, Tohoku University, 6-3 Aza-aoba, Aramaki, Sendai 980-8578, Japan

[‡] Graduate School of Pharmacy and Pharmaceutical Sciences, Juntendo University, 6-8-1 Hinode, Urayasu 279-0013, Japan

[§] Department of Chemistry, Institute of Pure and Applied Sciences, University of Tsukuba, 1-1-1 Tennodai, Tsukuba, Ibaraki 305-8571, Japan

[¢] Technology Research Association for Next Generation Natural Products Chemistry, 2-4-7 Aomi, Koto-ku, Tokyo 135-0064, Japan

[¶] National Institute of Advanced Industrial Science and Technology (AIST), 2-4-7 Aomi, Koto-ku, Tokyo 135-0064, Japan

[$] Japan Biological Informatics Consortium (JBIC), 2-4-7 Aomi, Koto-ku, Tokyo 135-0064, Japan

*corresponding author: doi_taka@mail.pharm.tohoku.ac.jp

Table of Contents.

1. Experimental Section
1.1. General techniques 3
1.2. Preparation of compounds 3
1.3. Comparison of NMR spectral data of natural product and synthetic **1a** 28
1.4. Comparison of acidic stability of **1a** and **1c** 29
1.5 Biological evaluation 30

2. ^{1}H and $^{13}C\{^{1}H\}$ NMR spectra of synthetic compounds
2.1. Compound **S1** 32
2.2. Compound **S2** 33
2.3. Compound **S4** 34

2.4. Compound **S5** 35
2.5. Compound **S7** 36
2.6. Compound **S8** 37
2.7. Compound **10** 38
2.8. Compound **11** 39
2.9. Compound **9a** 40
2.10. Compound **9b** 41
2.11. Compound **8** 42
2.12. Compound **13a** 43
2.13. Compound **6a** 44
2.14. Compound **5a** 45
2.15. Compound **13b** 46
2.16. Compound **6b** 47
2.17. Compound **5b** 48
2.18. Compound **15a** 49
2.19. JBIR-141 (**1a**) 50
2.20. Compound **15b** 51
2.21. 25-Methyl-JBIR-141 (**1b**) 52
2.22. Compound **15c** 53
2.23. 17-Methyl-JBIR-141 (**1c**) 54

1. Experimental Section

1.1 General techniques

All commercially available reagents were purchased from commercial suppliers and used as received. Dry THF and CH_2Cl_2 (Kanto Chemical Co.) were obtained by passing commercially available pre-dried, oxygen-free formulations through activated alumina column. All reactions in the solution phase were monitored by TLC carried out on Merck silica-gel plates (0.2 mm, 60F-254) with UV light, and visualized by *p*-anisaldehyde/H_2SO_4/EtOH solution, phosphomolybdic acid–EtOH solution or ninhydrin/AcOH/BuOH solution. Column chromatography was carried out with silica-gel 60 N (Kanto Chemical Co. 100–210 μm). ^{1}H NMR spectra (400 and 600 MHz) and ^{13}C{^{1}H} NMR spectra (100 and 150 MHz) were recorded on JEOL JNM-AL400, ECZL400S, JNM-ECA600, and ECZ600 spectrometers in the indicated solvent. Chemical shifts (δ) are reported in units parts per million (ppm) relative to the signal for internal TMS (0.00 ppm for ^{1}H) for solutions in chloroform-*d*. NMR spectral data are reported as follows: chloroform-*d* (7.26 ppm for ^{1}H, 77.00 ppm for ^{13}C), methanol-d_4 (3.31 ppm for ^{1}H and 49.00 ppm for ^{13}C) and DMSO-d_6 (2.50 ppm for ^{1}H, 39.52 ppm for ^{13}C) when internal standard is not indicated. Multiplicities are reported by using standard abbreviations, and coupling constants are given in hertz. High-resolution mass spectra (HRMS) were recorded on JEOL JMS-DX303 (for EI), MS-AX500 (for FAB), and Thermo Scientific Exactive Plus Orbitrap Mass Spectrometer (for ESI). IR spectra were recorded on a JASCO FTIR-4100 spectrophotometer. Only the strongest and/or structurally important absorption are reported as the IR data afforded in wavenumbers (cm^{-1}). Optical rotations were measured on a JASCO P-1010 polarimeter. Melting points were measured with Round Science Inc. RFS-10, and are not corrected.

1.2 Preparation of compounds

Methyl (*S*)-2-(1-(dimethylamino)ethyl)-5-methyloxazole-4-carboxylate hydrochloride (**S2**)

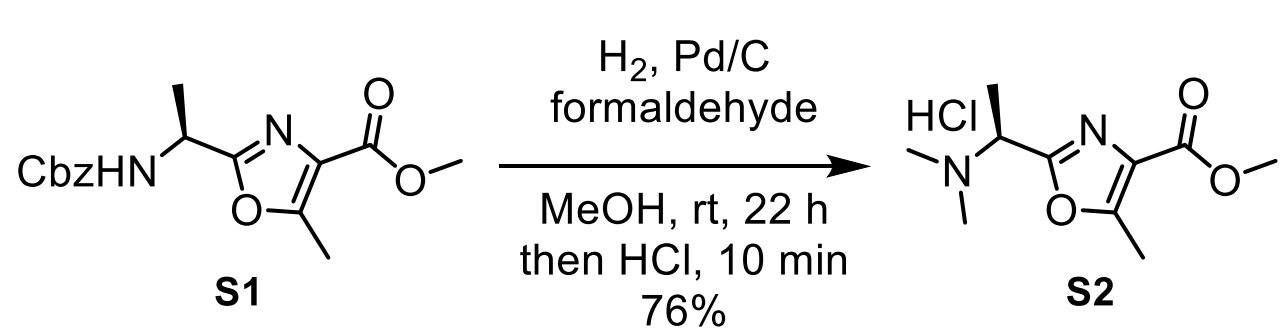

To a suspension of oxazole **S1**[1](905.0 mg, 2.84 mmol, 1.0 equiv) in MeOH (19 mL) were added aqueous 37% formaldehyde (1.3 mL, 17.06 mmol, 6.0 equiv) and 10% Pd/C (350 mg). The flask was purged with argon and was saturated with hydrogen under passive vacuum. After purging and back-filling with hydrogen three times, the reaction mixture was stirred under hydrogen at room temperature for 22 h. Upon completion, the resulting aqueous slurry was filtered through a pad of Celite® and the filtrate was

[1] (a) Phillips, A. J.; Uto, Y.; Wipf, P.; Reno, M. J.; Williams, D. R. *Org. Lett.* **2000**, *2*, 1165-1168. (b) Bagley, M. C.; Buck, R. T.; Hind, S. L.; Moody, C. J. *J. Chem. Soc., Perkin Trans. 1* **1998**, 591-600.

concentrated in vacuo. The residue was passed through a short-pad of silica-gel and the eluent was concentrated in vacuo. The residue was dissolved in Et_2O (1 ml). To this solution was added 1M HCl in Et_2O (8.5 mL, 8.53 mmol, 3.0 equiv). After being stirred for 10 min, the mixture was concentrated in vacuo. The residue was recrystallized from MeOH/Et_2O to afford **S2** (537.1 mg, 2.16 mmol, 76%) as a white solid. Mp 210 °C, $[\alpha]^{25}_D = -28$ (*c* 1.1, $CHCl_3$); ^1H NMR (400 MHz, $CDCl_3$): δ 4.59 (q, 1H, J = 7.2 Hz), 3.94 (s, 3H), 2.84 (brs, 3H), 2.80 (brs, 3H), 2.69 (s, 3H), 1.94 (d, 3H, J = 7.2 Hz); $^{13}C\{^1H\}$ NMR (100 MHz, $CDCl_3$): δ 161.5, 158.1, 154.6, 128.4, 57.6, 52.2, 42.8, 36.9, 14.7, 12.1; IR(neat): 2407, 1718, 1612, 1444, 1351, 1229, 1110, 1079 cm^{-1}; HRMS(EI) *m/z* calcd for $C_{10}H_{16}N_2O_3$ $[M–HCl]^+$ 212.1161, found 212.1156.

(*S*)-2-(1-(dimethylamino)ethyl)-5-methyloxazole-4-carboxylic acid hydrochloride (**7**)

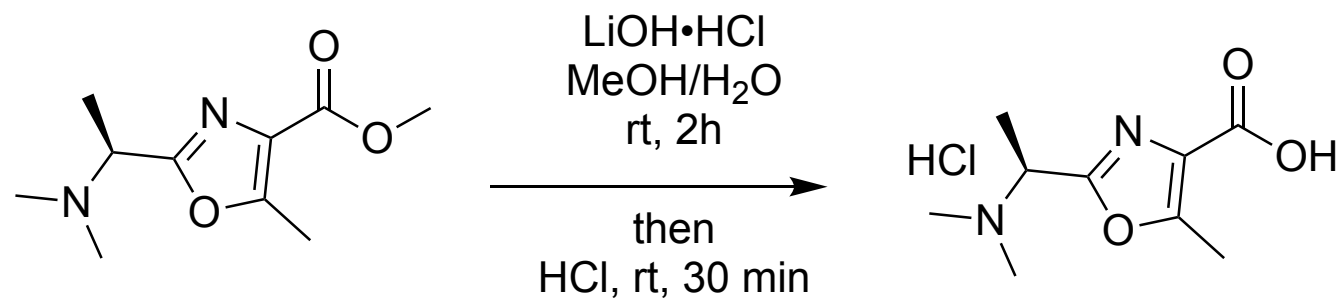

S2 **7**

To a solution of the methyl ester **S2** (150 mg, 603 µmol, 1.0 equiv) in MeOH/H_2O (3/1) (2 mL) was added lithium hydroxide monohydrate (88.6 mg, 2.11 mmol, 3.5 equiv) at room temperature under argon. After being stirred at the same temperature for 2 h, the reaction mixture was quenched with 1 M HCl in Et_2O (3.0 mL, 3.02 mmol, 5.0 equiv). After being stirred for 30 min, the mixture was concentrated in vacuo. The resulting crude carboxylic acid was used for the next reaction without further purification.

Benzyl *N,N*-dimethylalanyl-L-*allo*-threoninate (**S4**)

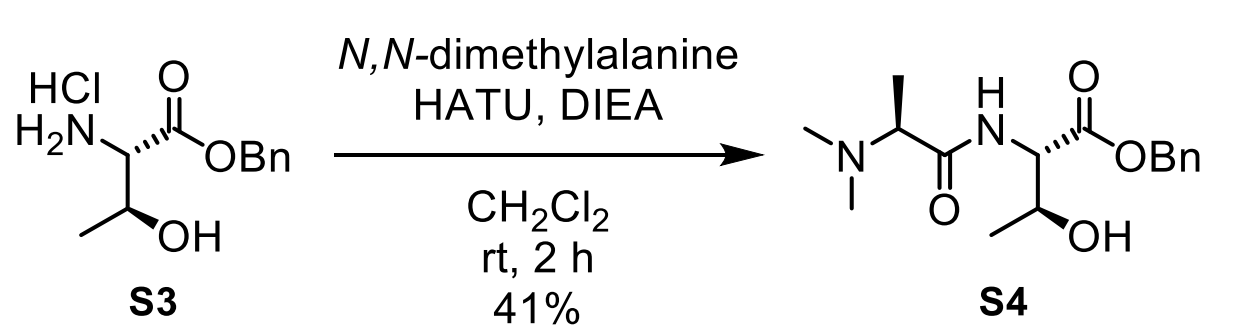

S3 **S4**

To a solution of the **S3**•HCl[2] (2.10 g, 8.54 mmol, 1.0 equiv) and *N,N*-dimethylalanine (1.00 g, 8.54 mmol, 1.0 equiv) in dry CH_2Cl_2 (34 mL) were added DIEA (3.0 mL, 17.1 mmol, 2.0 equiv) and HATU (3.57 g, 9.39 mmol, 1.1 equiv) at 0 °C under argon. After being stirred at room temperature for 2 h, the reaction mixture was quenched with saturated aqueous $NaHCO_3$ and the aqueous layer was extracted with ethyl acetate twice. The organic layers were washed with water and brine, dried with Na_2SO_4, and filtered. The

2 Cohen, S. B.; Halcomb, R. L. *J. Am. Chem. Soc.* **2002**, *124*, 2534-2543.

filtrate was concentrated in vacuo. The residue was purified by column chromatography on silica-gel (toluene/acetone = 2:1) to give benzyl *N,N*-dimethylalanyl-L-*allo*-threoninate (**S4**) (1.09 g, 3.53 mmol, 41%) as a colorless oil. $[\alpha]_D^{23}$ = +17 (*c* 0.50, $CHCl_3$); ^{1}H NMR (400 MHz, $CDCl_3$): δ 8.06 (d, *J* = 7.0 Hz, 1H), 7.36 (m, 5H), 5.24 (d, *J* = 12.2 Hz, 1H), 5.18 (d, *J* = 12.2 Hz, 1H), 4.71 (dd, *J* = 7.0, 3.6 Hz, 1H), 4.23 (m, 1H), 3.52 (br, 1H), 3.02 (q, *J* = 7.0 Hz, 1H), 2.25 (s, 6H), 1.23 (d, *J* = 7.0 Hz, 3H), 1.10 (d, *J* = 6.4 Hz, 3H); $^{13}C\{^1H\}$ NMR (100 MHz, $CDCl_3$): δ 175.8, 170.0, 135.0, 128.6, 128.5, 128.3, 69.0, 67.4, 64.5, 58.0, 42.2, 18.5, 11.8; IR(neat): 3368, 2977, 2787, 1742, 1662, 1512, 1261, 1188 cm^{-1}; HRMS(ESI) *m/z* calcd for $C_{16}H_{25}O_4N_2$ $[M+H]^+$ 309.1809, found 309.1808.

Oxazoline **S5**

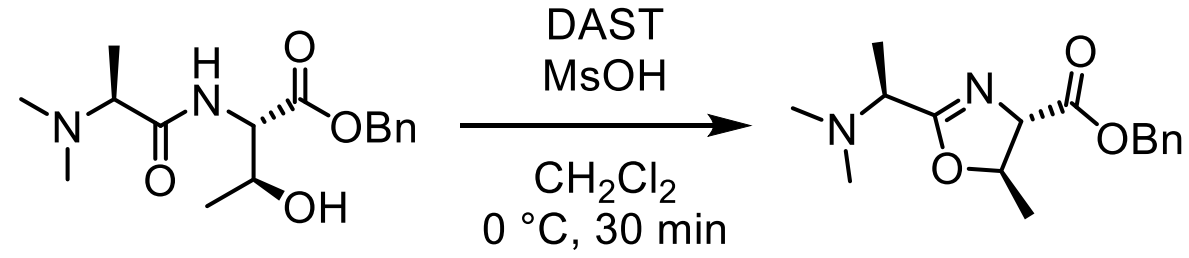

S4 **S5**

To a solution of dipeptide **S4** (1.12 g, 3.64 mmol, 1.0 equiv) and methanesulfonic acid (236 µL, 3.64 mmol, 1.0 equiv) in CH_2Cl_2 (24 mL) was added a solution of DAST (577 µL, 4.37 mmol, 1.2 equiv) dropwise at 0 °C under argon. After being stirred at the same temperature for 30 min, the reaction mixture was poured into saturated aqueous $NaHCO_3$ at 0 °C and the aqueous layer was extracted with CH_2Cl_2 twice. The organic layers were washed with brine, dried with Na_2SO_4, and filtered. The filtrate was concentrated in vacuo. The residue was purified by column chromatography on silica-gel (chloroform/methanol = 75:1) to give oxazoline **S5** (722 mg, 2.49 mmol, 68%) as a colorless oil. $[\alpha]_D^{23}$ = +47.4 (*c* 1.01, $CHCl_3$); ^{1}H NMR (400 MHz, $CDCl_3$): δ 7.35 (m, 5H), 5.20 (s, 2H), 4.82 (quint, *J* = 6.4 Hz, 1H), 4.29 (d, *J* = 6.4 Hz, 1H), 3.35 (q, *J* = 6.8 Hz, 1H), 2.31 (s, 6H), 1.41 (d, *J* = 6.4 Hz, 3H), 1.32 (d, *J* = 6.8 Hz, 3H); $^{13}C\{^1H\}$ NMR (100 MHz, $CDCl_3$): δ 170.9, 170.0, 135.3, 128.5, 128.4, 128.2, 78.7, 74.4, 67.1, 58.3, 42.0, 21.0, 15.2; IR(neat): 3360, 2932, 1740, 1656, 1455, 1266, 1175, 751, 698 cm^{-1}; HRMS(ESI) *m/z* calcd for $C_{16}H_{23}N_2O_3$ $[M+H]^+$ 291.1703, found 291.1700.

Acid **14a**

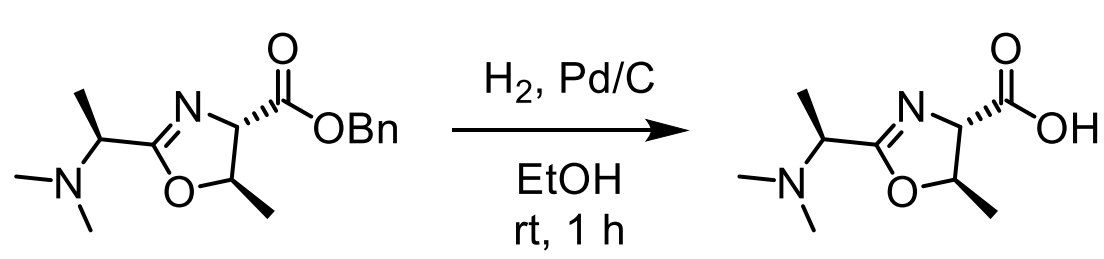

S5 **14a**

To a solution of benzyl ester **S5** (64 mg, 220 µmol, 1.2 equiv) in dry EtOH (1.1 mL) was added 10% Pd/C (20 mg, 30wt%) at room temperature under argon. The reaction vessel was purged with hydrogen gas three times. After being stirred at the same temperature for 1 h, the reaction mixture was filtered through a pad of Celite® and the filtrate was concentrated in vacuo. The residue was used for the next reaction without further purification.

Benzyl *N,N*-dimethyl-2-amino-2-methylpropanoyl-L-*allo*-threoninate (**S7**)

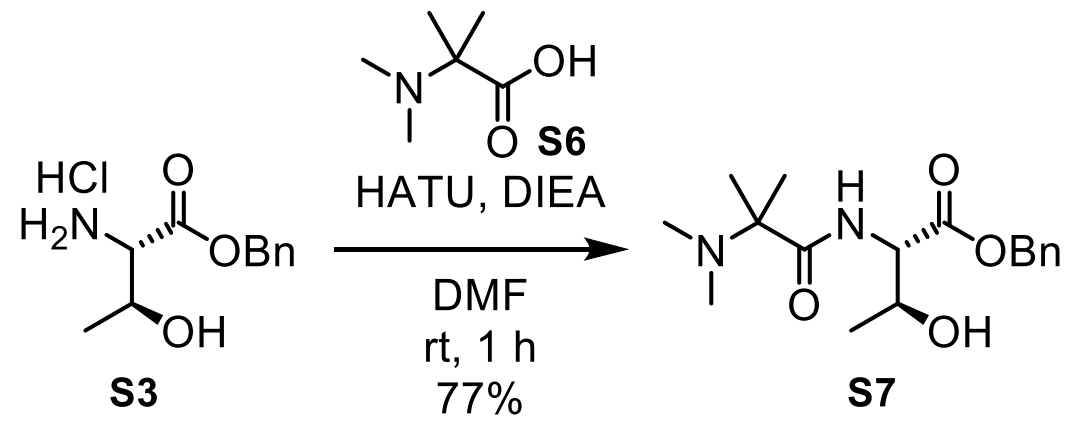

To a solution of the 2-(dimethylamino)-2-methylpropanoic acid (**S6**) (1.00 g, 7.62 mmol, 1.0 equiv) in DMF (25 mL), were added DIEA (2.7 mL, 15.3 mmol, 2.0 equiv), benzyl L-*allo*-threoninate hydrochloride (**S3**) (2.06 g, 8.39 mmol, 1.1 equiv) and HATU (3.48 g, 9.15 mmol, 1.2 equiv) at 0 °C under argon. After being stirred at room temperature for 1 h, the reaction mixture was quenched with saturated aqueous $NaHCO_3$ and the aqueous layer was extracted with ethyl acetate twice. The organic layers were washed with water, brine, dried with Na_2SO_4, and filtered. The filtrate was concentrated in vacuo. The residue was purified by column chromatography on silica-gel ($CHCl_3$/MeOH = 30:1) to give amide **S7** (1.88 g, 5.84 mmol, 77%) as a colorless oil. $[\alpha]_D^{16}$ = +5.0 (*c* 1.3, $CHCl_3$); ^{1}H NMR (400 MHz, $CDCl_3$): δ 8.17 (d, J = 7.6 Hz, 1H), 7.34–7.39 (m, 5H), 5.24 (d, J = 12.4 Hz, 1H), 5.17 (d, J = 12.4 Hz, 1H), 4.67 (dd, J = 7.6, 4.0 Hz, 1H), 4.23 (m, 1H), 3.46 (d, J = 6.0 Hz, 1H), 2.19 (s, 6H), 1.20 (s, 3H), 1.15 (s, 3H), 1.10 (d, J = 6.4 Hz, 3H); ^{13}C{^{1}H} NMR (100 MHz, $CDCl_3$): δ 178.8, 170.8, 135.0, 128.59, 128.55, 128.4, 69.2, 67.4, 63.5, 58.1, 39.3, 21.0, 18.9, 18.5; IR(neat): 3366, 2979, 1741, 1658, 1503, 1264, 1178 cm^{-1}; HRMS(EI) *m/z* calcd for $C_{17}H_{26}N_2O_4$ $[M]^+$ for 322.1893, found 322.1883.

Oxazoline benzyl ester **S8**

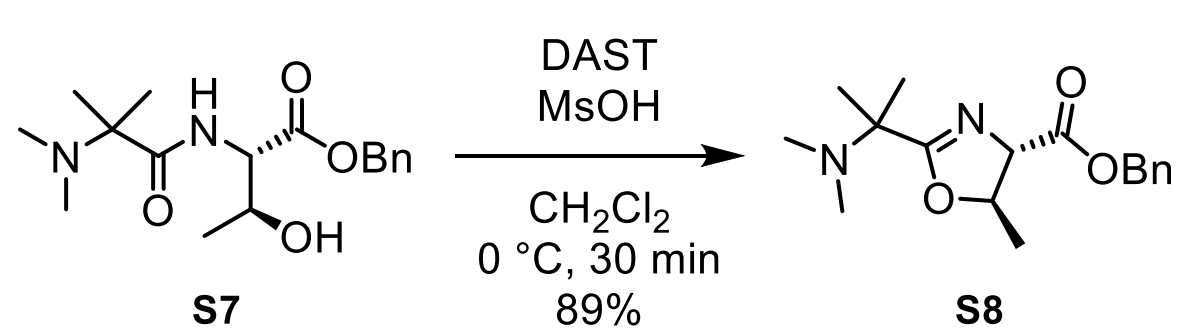

To a solution of dipeptide **S7** (762 mg, 2.36 mmol, 1.0 equiv) in CH_2Cl_2 (15.8 mL) were added methanesulfonic acid (153 µL, 2.36 mmol, 1.0 equiv) and DAST (343 µL, 2.60 mmol, 1.1 equiv) dropwise at 0 °C under argon. After being stirred at the same temperature for 30 min, the reaction mixture was poured into saturated aqueous $NaHCO_3$ at 0 °C and the aqueous layer was extracted with CH_2Cl_2 twice. The organic layers were washed with brine, dried with Na_2SO_4, and filtered. The filtrate was concentrated in vacuo. The residue was purified by column chromatography on silica-gel (chloroform/methanol = 30:1) to give oxazoline **S8** (643 mg, 2.11 mmol, 89%) as a colorless oil. $[\alpha]_D^{16}$ = +125 (*c* 0.90, $CHCl_3$); ^{1}H NMR (400 MHz, $CDCl_3$): δ 7.32–7.38 (m, 5H), 5.22 (d, J = 12.4 Hz, 1H), 5.17 (d, J = 12.4 Hz, 1H), 4.81 (quint, J = 6.4 Hz, 1H), 4.30 (d, J = 6.4 Hz, 1H), 2.27 (s, 6H), 1.40 (d, J = 6.4 Hz, 3H), 1.35 (s, 3H), 1.33 (s, 3H);

$^{13}C\{^1H\}$ NMR (100 MHz, $CDCl_3$): δ 173.2, 171.0, 135.3, 128.5, 128.3, 128.2, 78.8, 74.4, 67.0, 58.2, 39.0, 22.6, 21.0, 20.7; IR(neat): 2981, 1741, 1639, 1251, 1173, 1131 cm^{-1}; HRMS(ESI) *m/z* calcd for $C_{17}H_{23}N_2O_3$ [*M*–H]$^-$ for 303.1714, found 303.1707.

Acid **14b**

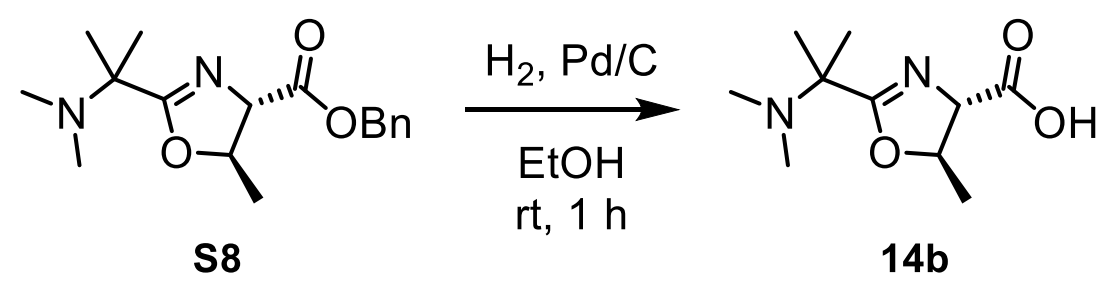

To a solution of benzyl ester **S8** (111 mg, 365 μmol, 1.2 equiv) in dry EtOH (1.8 mL) was added 10% Pd/C (35 mg, 32wt%) at room temperature under argon. The reaction vessel was purged with hydrogen gas three times. After being stirred at the same temperature for 1 h, the reaction mixture was filtered through a pad of Celite® and the filtrate was concentrated in vacuo. The residue was used for the next reaction without further purification.

Benzyl (*S*)-2-*tert*-butyldimethylsilyloxy-3-methylbutanoate (**S10**)

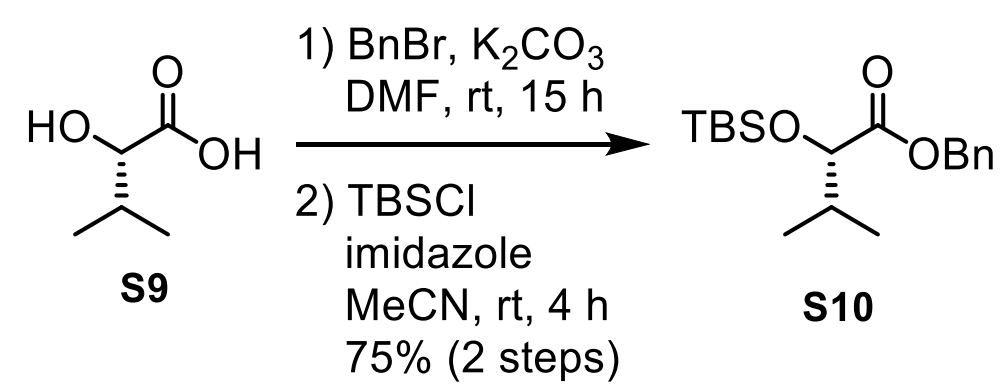

To a solution of acid **S9** (BLDpharm, 7.00 g, 59.26 mmol, 1 equiv) in DMF (119 mL) were added potassium carbonate (4.91 g, 35.55 mmol, 0.6 equiv) and benzyl bromide (7.4 mL, 62.22 mmol, 1.05 equiv) at 0 °C under argon. After being stirred for 15 h at room temperature, the reaction mixture was filtered through a pad of Celite® and the filtrate was washed with 1 M aqueous HCl. The aqueous layer was extracted with ethyl acetate twice. The organic layer was washed with 1 M aqueous HCl, saturated aqueous $NaHCO_3$ and brine, dried with $MgSO_4$, and filtered. The filtrate was concentrated in vacuo. The residue was used for the next reaction without further purification.

To a solution of the crude residue and imidazole (4.44 g, 65.18 mmol, 1.1 equiv) in dry MeCN (99 mL) was added TBSCl (9.38 g, 62.22 mmol, 1.05 equiv) at 0 °C under argon. After being stirred at room temperature for 4 h, the reaction mixture was quenched with water. The aqueous layer was extracted with CH_2Cl_2 twice. The organic layer was washed with brine, dried with Na_2SO_4, and filtered. The filtrate was concentrated in vacuo. The residue was purified by column chromatography on silica-gel (hexane/ethyl acetate = 40:1) to give ester **S10** (14.27 g, 44.24 mmol, 75% over 2 steps) as a colorless oil. $[\alpha]_D^{19}$ = +39.4 (*c* 1.02, $CHCl_3$); ^1H NMR (400 MHz, $CDCl_3$): δ 7.26–7.29 (m, 5H), 5.12 (d, *J* = 12.2 Hz, 1H), 5.07 (d, *J* = 12.2 Hz, 1H), 3.99 (d, *J* = 4.4 Hz, 1H), 2.07 (m, 1H), 0.85–0.92 (m, 15H), 0.01 (s, 3H), 0.00 (s, 3H); $^{13}C\{^1H\}$ NMR (100 MHz, $CDCl_3$): δ 172.5, 135.6, 128.2, 128.1, 127.9, 76.6, 65.8, 32.4, 25.4, 18.8, 17.9,

16.4, –5.3, –5.8; IR(neat): 2959, 2858, 1753, 1731, 1253, 1140, 1071, 859, 836, 777 cm^{-1}; HRMS(ESI) *m/z* calcd for $C_{18}H_{31}O_3Si$ $[M+H]^+$ 323.2037, found 323.2038.

Allyl ester **10**

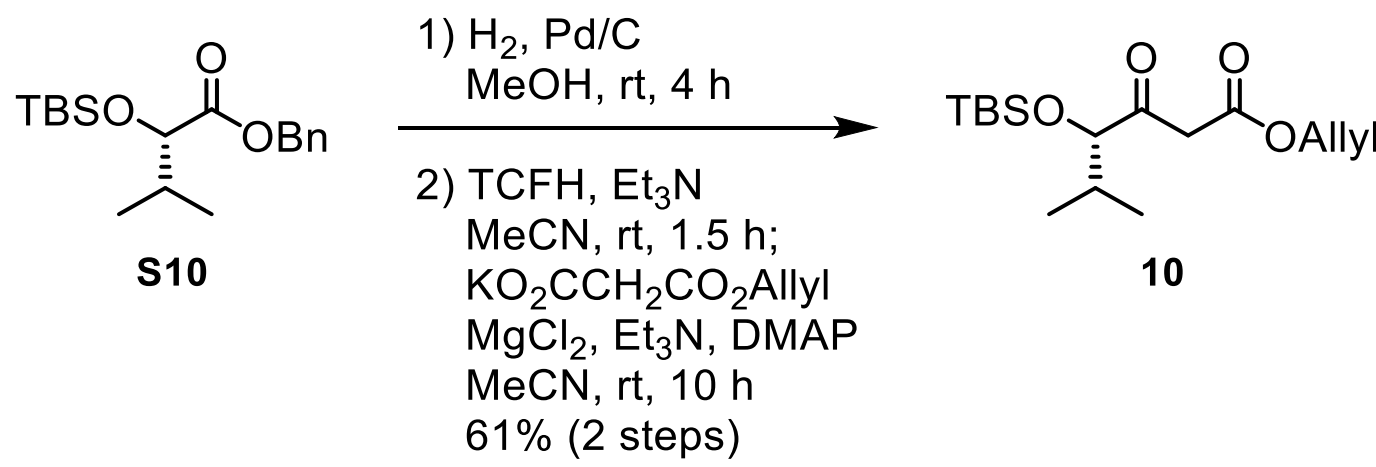

To a solution of benzyl ester **S10** (12.03 g, 37.31 mmol, 1 equiv) in dry MeOH (93 mL) was added 10% Pd/C (2.50 g) at room temperature under argon. The reaction vessel was purged with hydrogen gas three times. After being stirred at the same temperature for 4 h, the reaction mixture was filtered through a pad of Celite® and the filtrate was concentrated in vacuo. The residue was used for the next reaction without further purification.

To a solution of crude carboxylic acid and triethylamine (10.9 mL, 78.35 mmol, 2.1 equiv) in dry MeCN (62 mL) was added TCFH (10.99 g, 39.18 mmol, 1.05 equiv) at 0 °C under argon. After being stirred at room temperature for 1.5 h, the reaction mixture was added to a solution of crude mono allyl potassium malonate (12.24 g, 67.16 mmol, 1.8 equiv), anhydrous $MgCl_2$ (6.75 g, 70.89 mmol, 1.9 equiv) and triethylamine (9.4 mL, 67.16 mmol, 1.8 equiv) in dry MeCN (124 mL) at 0 °C. To the suspension was added DMAP (2.28 g, 18.66 mmol, 0.50 equiv) at the same temperature. After being stirred at room temperature for 15 h, the reaction mixture was quenched with 1 M aqueous HCl at 0 °C and the aqueous layer was extracted with ethyl acetate twice. The organic layers were washed with 1 M aqueous HCl, saturated aqueous $NaHCO_3$ twice and brine, dried with $MgSO_4$, and filtered. The filtrate was concentrated in vacuo. The residue was purified by column chromatography on silica-gel (hexane/ethyl acetate = 20:1) to afford β-ketoester **10** (7.16 g, 22.8 mmol, 61% in 2 steps) as a colorless oil. $[\alpha]_D^{23} = -42$ (*c* 0.70, $CHCl_3$); Keto form : enol form = 5.8 : 1 in the ^{1}H NMR spectrum, ^{1}H NMR (600 MHz, $CDCl_3$, major tautomer): δ 11.91 (s, 1H), 5.89 (m, 1H), 5.32 (dd, J = 17.4, 1.2 Hz, 1H), 5.25 (dd, J = 10.4, 1.2 Hz, 1H), 4.62 (d, J = 6.0 Hz, 2H), 3.78 (d, J = 6.0 Hz, 1H), 3.61 (d, J = 11.2 Hz, 1H), 3.59 (d, J = 11.2 Hz, 1H), 1.93 (m, 1H), 0.91–0.96 (m, 15H), 0.06 (s, 3H), 0.04 (s, 3H); $^{13}C\{^1H\}$ NMR (125 MHz, $CDCl_3$, a mixture of tautomers): δ 206.0, 180.0, 172.6, 167.4, 132.3, 131.8, 118.6, 118.3, 88.5, 83.4, 76.7, 65.9, 64.8, 45.0, 32.8, 32.8, 25.9, 25.8, 19.5, 18.6, 18.2, 18.1, 17.6, 16.3, −4.6, −4.9, −5.0, −5.1.; IR(neat): 2958, 2932, 2859, 1753, 1722, 1649, 1311, 1254, 1216, 1065, 839, 778 cm^{-1}; HRMS(ESI) *m/z* calcd for $C_{16}H_{30}O_4NaSi$ $[M+Na]^+$ 337.1806, found 337.1801.

(*S*,*Z*)-4-((*tert*-Butyldimethylsilyl)oxy)-5-methyl-3-(naphthalen-2-ylmethoxy)hex-2-enoic acid (**11**)

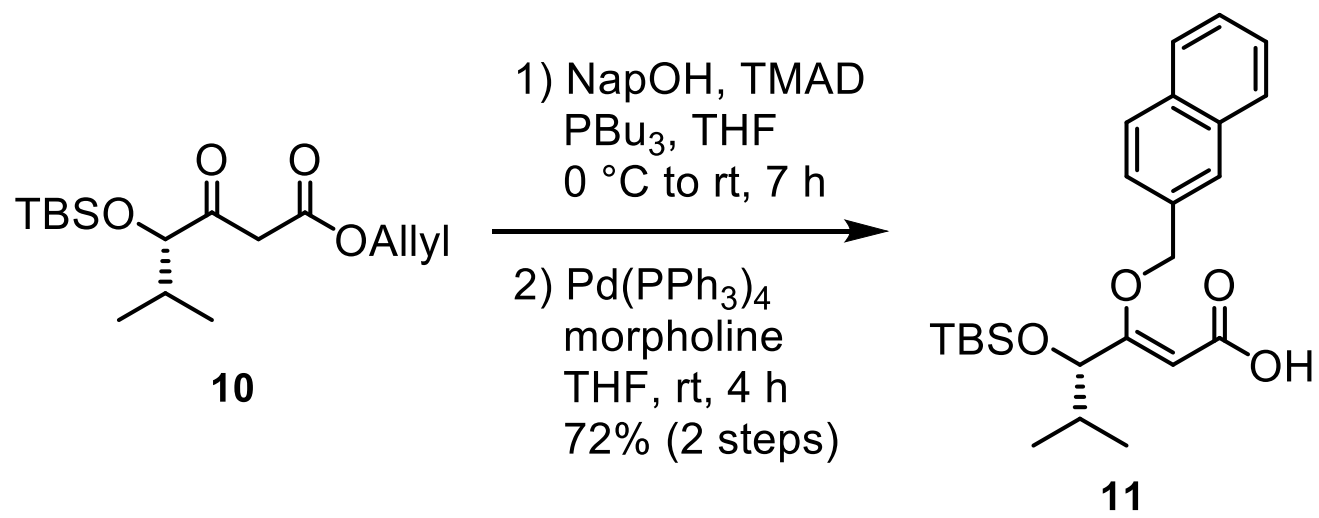

To a solution of *β*-ketoester **10** (720.2 mg, 2.29 mmol, 1.0 equiv), 2-naphthalenmethanol (579.6 mg, 3.66 mmol, 1.6 equiv), and tributylphosphine (960 μL, 3.89 mmol, 1.7 equiv) in dry THF (4.6 mL) was added *N,N,N',N'*-tetramethylazodicarboxamide (630.9 mg, 3.66 mmol, 1.6 equiv) at 0 °C under argon. After being stirred at room temperature for 7 h, the reaction mixture was quenched with water at 0 °C and the aqueous layer was extracted with hexane twice. The combined organic layers were washed with methanol and brine, dried with $MgSO_4$, and filtered. The filtrate was concentrated in vacuo. The resulting residue was used for the next reaction without further purification.

To a solution of the crude residue and morpholine (790 μL, 9.16 mmol, 4.0 equiv) in dry THF (6.5 mL) was added $Pd(PPh_3)_4$ (132.3 mg, 114.5 μmol, 0.05 equiv) at room temperature under argon. After being stirred at the same temperature for 4 h, the reaction mixture was quenched with 1 M aqueous HCl and the aqueous layer was extracted with ethyl acetate twice. The combined organic layers were washed with brine, dried with $MgSO_4$, and filtered. The filtrate was concentrated in vacuo. The residue was purified by column chromatography on silica-gel (eluted with hexane/ethyl acetate = 4:1) to afford carboxylic acid **11** (680.3 mg, 1.64 mmol, 72% over 2 steps) as a pale-yellow oil. $[\alpha]_D^{23}$ = +21 (*c* 1.1, $CHCl_3$); ^{1}H NMR (400 MHz, $CDCl_3$, major isomer) *δ* 7.83−7.90 (m, 4H, h, i, l and m), 7.47−7.54 (m, 3H, g, j and k), 5.56 (d, *J* = 11.8 Hz, 1H, f), 5.47 (s, 1H, n), 5.26 (d, *J* = 11.8 Hz, 1H, f), 4.05 (d, *J* = 4.8 Hz, 1H, c), 2.03 (m, 1H, d), 0.98 (d, *J* = 6.6 Hz, 3H, e), 0.92 (d, *J* = 6.6 Hz, 3H, e), 0.91 (s, 9H, a), 0.01 (s, 3H, b), −0.09 (s, 3H, b); ^{13}C{^{1}H} NMR (100 MHz, $CDCl_3$, major isomer) *δ* 172.5, 168.7, 133.4, 133.3, 133.2, 128.7, 128.0, 127.7, 127.2, 126.4, 126.4, 125.5, 98.6, 74.8, 32.4, 25.8, 20.0, 18.1, 16.3, −4.6, −5.3; IR(neat): 2958, 2930, 1685, 1621, 1253, 1230, 1062, 852, 838, 813 cm^{-1}; HRMS(FAB) *m/z* calcd for $C_{24}H_{33}O_4Si$ [*M*–H]$^-$ 413.2154, found 413.2140.

Methyl *N*-((*S*,*Z*)-4-hydroxy-5-methyl-3-(naphthalen-2-ylmethoxy)hex-2-enoyl)-*N*-methyl-L-alaninate (**9a**)

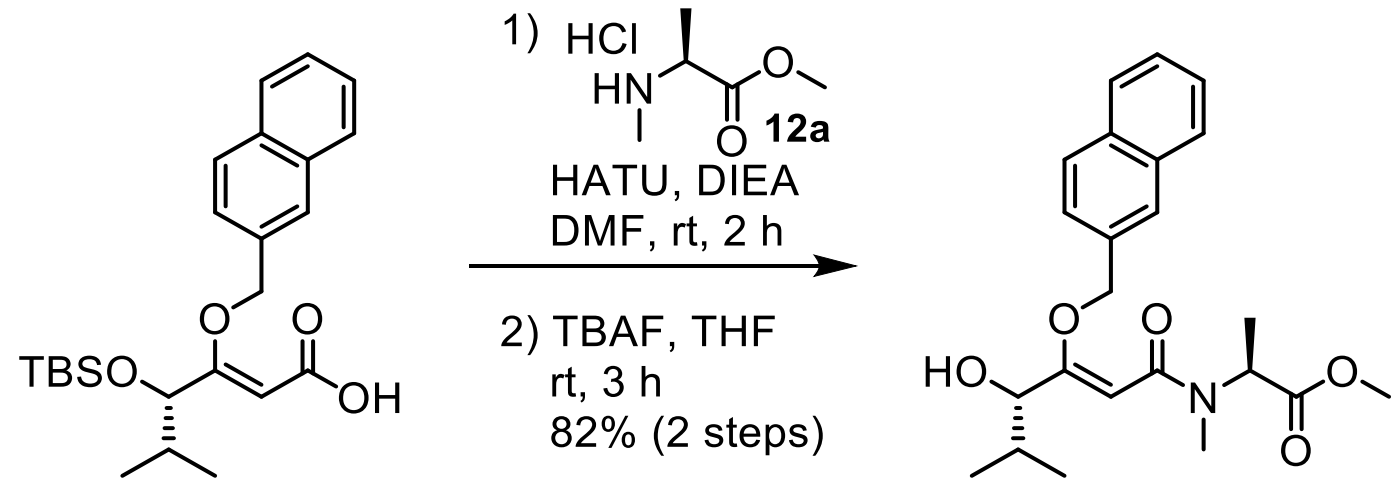

To a solution of acid **11** (313.5 mg, 756.1 μmol, 1.0 equiv) and H-MeAla-OMe•HCl (**12a**) (127.8 mg, 831.7 μmol, 1.1 equiv) in dry DMF (2.2 mL) were added DIEA (395 μL, 2.27 mmol, 3.0 equiv) and HATU (316.3 mg, 831.7 μmol, 1.1 equiv) at 0 °C under argon. After being stirred at room temperature for 2 h, the reaction mixture was quenched with 1 M aqueous HCl and the aqueous layer was extracted with ethyl acetate twice. The combined organic layers were washed with 1 M aqueous HCl, saturated aqueous $NaHCO_3$ and brine, dried with $MgSO_4$, and filtered. The filtrate was concentrated in vacuo. The resulting crude amide was used for the next reaction without further purification.

To a solution of the crude residue in THF (1.5 mL) was added 1.0 M TBAF in THF (2.3 mL, 2.27 mmol, 3.0 equiv) at room temperature. After being stirred at the same temperature for 3 h, the reaction mixture was quenched with $CaCO_3$. The mixture was further stirred at the same temperature for 10 min and was filtered through a pad of Celite®. The filtrate was concentrated in vacuo. The residue was purified by column chromatography on silica-gel (eluted with hexane/ethyl acetate = 1:1) to afford alcohol **9a** (246.6 mg, 617 μmol, 82%) as a colorless oil. $[\alpha]_D^{22} = -8.0$ (*c* 1.2, $CHCl_3$); ^{1}H NMR (400 MHz, $CDCl_3$, major isomer) *δ* 7.78−7.85 (m, 4H), 7.43−7.51 (m, 3H), 5.42 (s, 0.65H), 5.40 (s, 0.35H), 5.37 (d, *J* = 12.0 Hz, 0.5H), 5.34 (d, *J* = 12.0 Hz, 0.2H), 5.31 (d, *J* = 12.0 Hz, 0.3H), 5.27 (q, *J* = 7.2 Hz, 0.40H), 5.21 (q, *J* = 7.2 Hz, 0.34H), 5.10 (d, *J* = 12.0 Hz, 0.47H), 5.06 (d, *J* = 12.0 Hz, 0.13H), 4.99 (d, *J* = 12.0 Hz, 0.4H), 4.86 (q, *J* = 7.2 Hz, 0.13H), 4.79 (q, *J* = 7.2 Hz, 0.13H), 3.78−3.85 (m, 1H), 3.72 (s, 0.38H), 3.70 (s, 0.50H), 3.69 (s, 0.98H), 2.67 (s, 1.14H), 3.06 (s, 1.2H), 3.02 (s, 0.97H), 2.90 (s, 0.46H), 2.89 (s, 0.37H), 1.92−2.06 (m, 2H), 1.45 (d, *J* = 7.2 Hz, 1.2H), 1.43 (d, *J* = 7.2 Hz, 1.1H), 1.41 (d, *J* = 7.2 Hz, 0.35H), 1.32 (d, *J* = 7.2 Hz, 0.35H), 0.93−1.00 (m, 6H); ^{13}C{^{1}H} NMR (100 MHz, $CDCl_3$, major isomer) *δ* 172.3, 172.2, 171.7, 171.6, 168.8, 168.2, 165.2, 164.2, 134.46, 134.44, 134.38, 134.27, 133.14, 133.11, 133.0, 128.21, 128.19, 128.0, 127.9, 127.6, 126.97, 126.94, 126.86, 126.85, 126.2, 126.2, 126.1, 126.1, 126.1, 125.9, 125.7, 125.6, 96.9, 96.3, 95.4, 77.8, 73.7, 73.5, 73.4, 73.2, 56.5, 56.4, 52.43, 52.40, 52.2, 51.7, 32.9, 32.6, 31.8, 31.75, 31.69, 28.4, 28.3, 19.6, 19.54, 19.50, 16.81, 16.78, 16.64, 16.56, 15.12, 15.10, 14.4, 14.3; IR(neat): 3372, 2958, 1742, 1651, 1604, 1462, 1279, 1213, 1136, 1031 cm^{-1}; HRMS(EI) *m/z* calcd for $C_{23}H_{29}NO_5$ $[M]^+$ 399.2046, found 399.2049.

Methyl (*S*,*Z*)-2-(4-hydroxy-*N*,5-dimethyl-3-(naphthalen-2-ylmethoxy)hex-2-enamido)-2-methylpropanoate (**9b**)

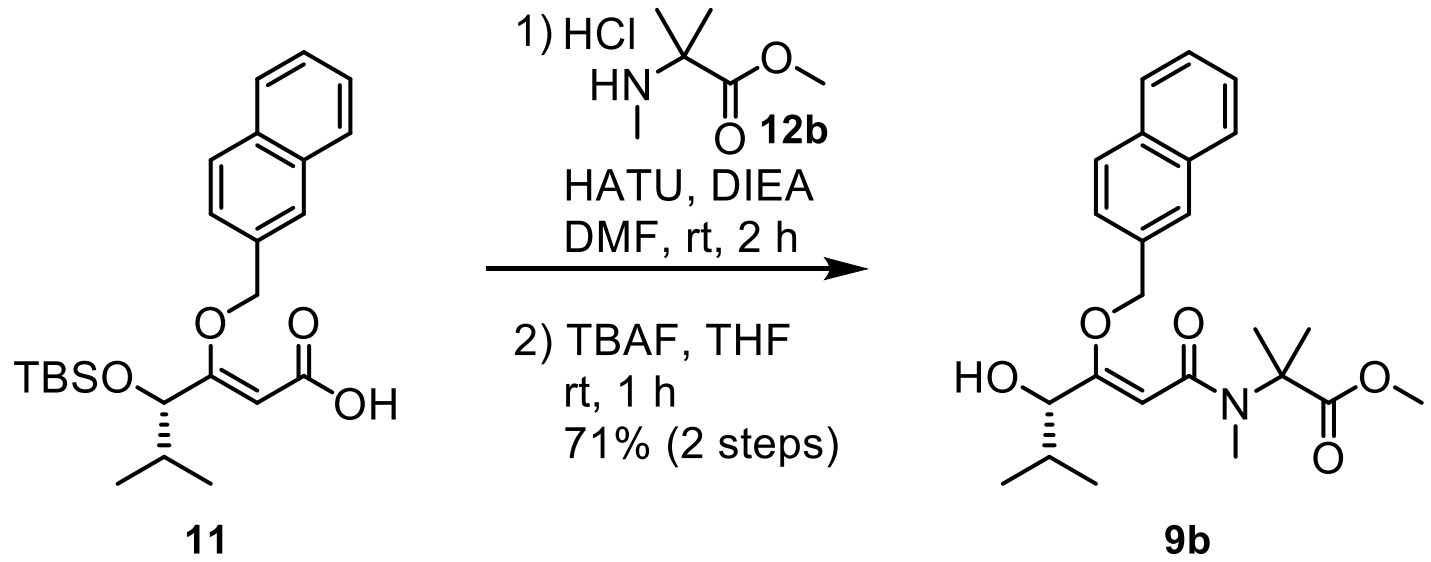

To a solution of acid **11** (415.7 mg, 1.00 mmol, 1 equiv) and **12b** (184.9 mg, 1.10 mmol, 1.1 equiv) in DMF (3.0 mL) were added DIEA (524 µL, 3.01 mmol, 3.0 equiv) and HATU (419.4 mg, 1.10 mmol, 1.1 equiv) at 0 °C under argon. After being stirred at room temperature for 2 h, the reaction mixture was quenched with 1 M aqueous HCl and the aqueous layer was extracted with ethyl acetate twice. The combined organic layers were washed with 1 M aqueous HCl, saturated aqueous $NaHCO_3$ and brine, dried with $MgSO_4$, and filtered. The filtrate was concentrated in vacuo. The resulting crude amide was used for the next reaction without further purification.

To a solution of the crude residue in THF (2.0 mL) was added 1.0 M TBAF in THF (3.0 mL, 3.0 mmol, 3.0 equiv) at room temperature. After being stirred at the same temperature for 1 h, the reaction mixture was quenched with $CaCO_3$. The mixture was further stirred at the same temperature for 10 min and was filtered through a pad of Celite®. The filtrate was concentrated in vacuo. The residue was purified by column chromatography on silica-gel (eluted with hexane/ethyl acetate = 1:1) to afford alcohol **9b** (294.7 mg, 713 µmol, 71%) as a colorless oil. $[\alpha]_D^{25} = -20$ (*c* 1.3, $CHCl_3$); ^1H NMR (400 MHz, $CDCl_3$, major isomer) δ 7.80−7.85 (m, 4H), 7.44−7.51 (m, 3H), 5.38 (s, 1H), 5.31 (d, J = 11.6 Hz, 1H), 4.97 (d, J = 11.6 Hz, 1H), 3.81 (brs, 1H), 3.67 (s, 3H), 3.08 (s, 3H), 1.96−2.02 (m, 2H), 1.48 (s, 6H), 0.98 (d, J = 6.8 Hz, 3H), 0.94 (d, J = 6.8 Hz, 3H); $^{13}C\{^1H\}$ NMR (100 MHz, $CDCl_3$, major isomer) δ 174.7, 167.8, 163.5, 134.4, 133.1, 133.0, 128.3, 128.0, 127.7, 126.9, 126.2, 126.1, 125.8, 97.5, 78.1, 73.8, 60.2, 52.2, 32.1, 31.9, 23.3, 23.1, 19.5, 16.9; IR(neat): 3383, 2958, 1739, 1651, 1603, 1469, 1382, 1281, 1159, 1125, 754 cm^{-1}; HRMS(EI) *m/z* calcd for $C_{24}H_{31}NO_5$ $[M]^+$ 413.2202, found 413.2182.

Carboxylic acid **8**

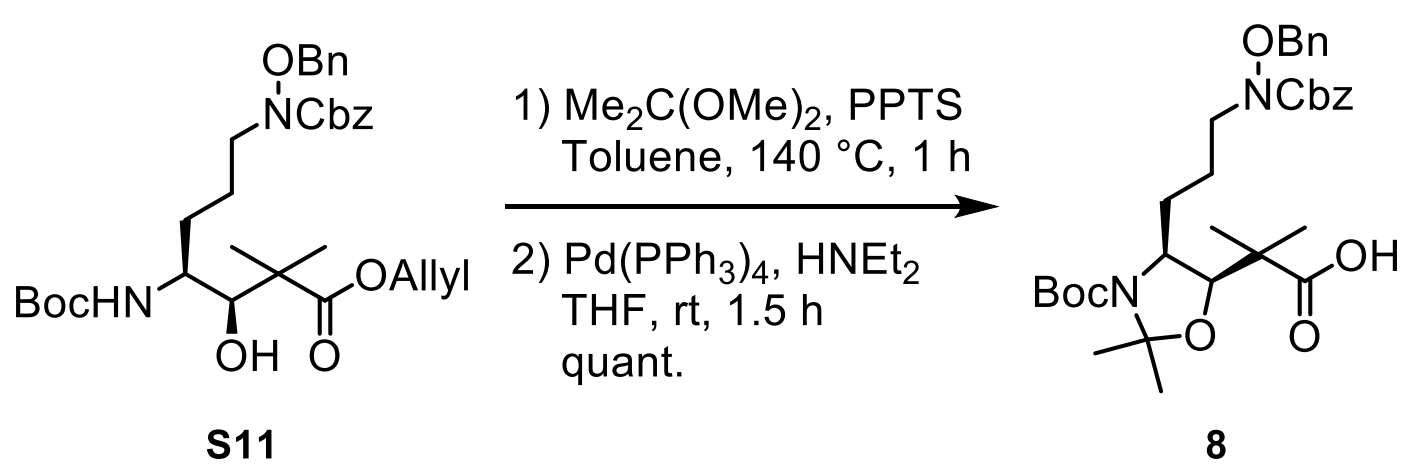

To a solution of alcohol **S11**[3] (604 mg, 1.03 mmol, 1 equiv) and 2,2-dimethoxypropane (1.26 mL, 10.3 mmol, 10 equiv) in toluene (6.0 mL) was added PPTS (77.8 mg, 310 µmol, 0.30 equiv) at room temperature. After being stirred at 140 °C with dean-stark apparatus for 1 h, the reaction mixture was poured into saturated aqueous $NaHCO_3$ at room temperature and the aqueous layer was extracted with ethyl acetate. The organic layer was washed with brine, dried with $MgSO_4$, and filtered. The filtrate was concentrated in vacuo. The residue was used for the next reaction without further purification.

To a solution of the crude residue and diethylamine (536 µL, 5.17 mmol, 5.0 equiv) in dry THF (10 mL) was added $Pd(PPh_3)_4$ (55.8 mg, 48.3 µmol, 0.05 equiv) at room temperature under argon. After being stirred at the same temperature for 1.5 h, the reaction mixture was concentrated in vacuo. The residue was purified by flash column chromatography on silica-gel ($CHCl_3$/MeOH = 90:1 to 75:1) to give acid **8** (635 mg, 1.09 mmol, quant.) as a yellow oil. $[\alpha]_D^{24} = -22.8$ (*c* 0.595, $CHCl_3$); ^1H NMR (600 MHz, DMSO-d_6, 60 °C): 7.33–7.38 (m, 10H), 5.13 (s, 2H), 4.81 (s, 2H), 4.04 (d, *J* = 3.2 Hz, 1H), 3.97 (broad, 1H), 3.45 (t, *J* = 4.2 Hz, 2H), 1.49–1.60 (m, 3H), 1.47 (s, 3H), 1.45 (s, 3H), 1.45–1.49 (m, 1H), 1.36 (s, 9H), 1.22 (s, 3H), 1.13 (s, 3H); ^{13}C NMR (150 MHz, DMSO-d_6, 60 °C): δ 176.7, 156.0, 136.1, 135.2, 128.6, 128.1, 128.0, 127.9, 127.6, 127.3, 91.2, 79.6, 78.7, 75.8, 66.6, 58.3, 48.8, 42.4, 27.74, 27.66, 25.5, 19.7; IR(neat): 2978, 2936, 1694, 1455, 1386, 1274, 1173, 1027, 750, 698 cm^{-1}; HRMS(ESI) *m/z* calcd for $C_{32}H_{44}N_2O_8Na$ $[M+Na]^+$ 607.2990, found 607.2989.

Ester **13a**

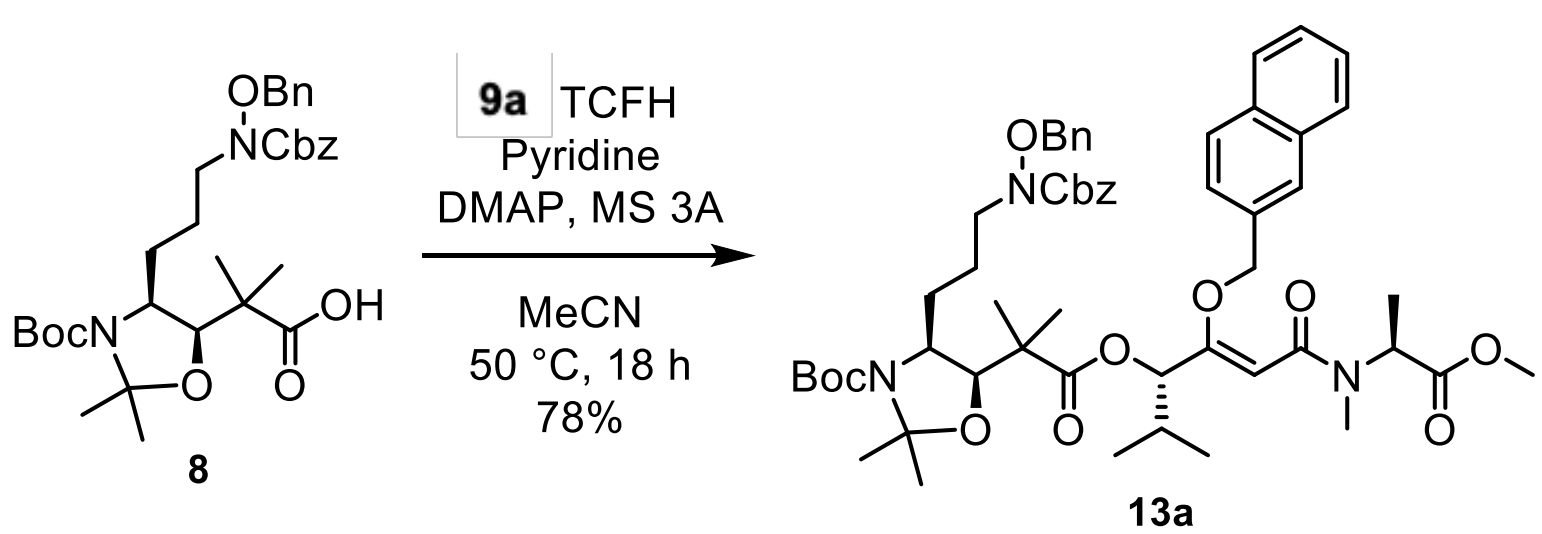

To a solution of **8** (1.01 g, 1.74 mmol, 1.0 equiv) in dry MeCN (8.7 mL) were added alcohol **9a** (693 mg, 1.74 mmol, 1.0 equiv), pyridine (1.4 mL, 17.35 mmol, 10 equiv), activated MS 3A (870 mg), TCFH (974 mg, 3.47 mmol, 2.0 equiv) and DMAP (424 mg, 3.47 mmol, 2.0 equiv) at 0 °C under argon. After being stirred at 50 °C for 18 h, the mixture was diluted with MeCN and the solution was filtered through a pad of Celite®, and the filtrate was concentrated in vacuo. The residue was diluted with ethyl acetate and the solution was quenched with 10% aqueous citric acid at 0 °C. The aqueous layer was extracted with ethyl acetate twice. The combined organic layers were washed with saturated aqueous $NaHCO_3$ and brine twice, dried with $MgSO_4$, and filtered. The filtrate was concentrated in vacuo. The residue was purified by

---

[3] Yasoshima, K.; Yoshida, M.; Doi, T. *Bull. Chem. Soc. Jpn.* **2022**, *95*, 830-832.

column chromatography on silica-gel (eluted with hexane/ethyl acetate = 2:1) to afford ester **13a** (1.30 g, 1.35 mmol, 78%) as a pale-yellow amorphous solid. $[\alpha]_D^{25} = -28$ (*c* 1.1, $CHCl_3$); ^{1}H NMR (600 MHz, $CDCl_3$, a mixture of rotamers) *δ* 7.79−7.83 (m, 4H), 7.43−7.48 (m, 3H), 7.30−7.36 (m, 10H), 5.23−5.38 (m, 2H,), 5.16−5.22 (m, 1H), 5.17 (s, 2H), 5.08 (d, *J* = 11.4 Hz, 0.6H), 4.98 (d, *J* = 11.4 Hz, 0.4H), 4.94 (d, *J* = 6.6 Hz, 1H), 4.83 (s, 2H), 4.20−4.22 (m, 0.5H), 4.10−4.14 (m, 1H), 4.02−4.07 (m, 0.5H), 3.68 (s, 0.6H), 3.66 (s, 2H), 3.65 (s, 0.4H), 3.47−3.56 (m, 1H), 3.36−3.45 (m, 1H), 2.99 (s, 1.3H), 2.98 (s, 1.0H), 2.86 (s, 0.7H), 2.19 (quint, 1H, *J* = 6.6 Hz), 1.64−1.70 (m, 2H), 1.47−1.53 (m, 7H), 1.34−1.42 (m, 13H), 1.11−1.32 (m, 6H), 0.95 (d, *J* = 6.6 Hz, 3H), 0.95 (d, *J* = 6.6 Hz, 3H); $^{13}C\{^1H\}$ NMR (150 MHz, $CDCl_3$, a mixture of rotamers) *δ* 175.4, 175.4, 175.2, 172.2, 172.2, 172.1, 171.6, 171.6, 167.5, 167.5, 167.3, 167.3, 159.2, 158.9, 158.8, 157.1, 152.8, 152.0, 136.2, 136.1, 135.3, 134.6, 134.5, 134.5, 134.5, 134.5, 134.4, 134.4, 134.4, 134.4, 133.2, 133.0, 129.3, 129.2, 128.5, 128.4, 128.4, 128.3, 128.1, 128.0, 127.9, 127.7, 126.6, 126.5, 126.5, 126.1, 126.1, 126.0, 125.6, 98.9, 98.9, 98.7, 92.5, 92.1, 80.1, 79.9, 79.8, 79.6, 79.5, 79.5, 79.3, 77.1, 73.6, 73.5, 73.5, 73.3, 67.6, 67.5, 58.9, 58.4, 56.4, 56.3, 52.4, 52.3, 52.2, 52.0, 51.8, 49.9, 49.7, 44.0, 32.8, 32.7, 32.6, 30.4, 29.7, 28.8, 28.7, 28.4, 28.3, 28.2, 27.4, 25.0, 24.6, 24.3, 23.4, 23.3, 21.3, 21.3, 20.7, 19.0, 19.0, 17.9, 17.8, 17.8, 15.2, 15.1, 14.3, 14.3; IR(neat): 2974, 1737, 1693, 1386, 1273, 1213, 1174, 1139, 1116, 1095 cm^{-1}; HRMS(EI) *m/z* calcd for $C_{55}H_{71}N_3O_{12}$ $[M]^+$ 965.5038, found 965.5039.

Amide **6a**

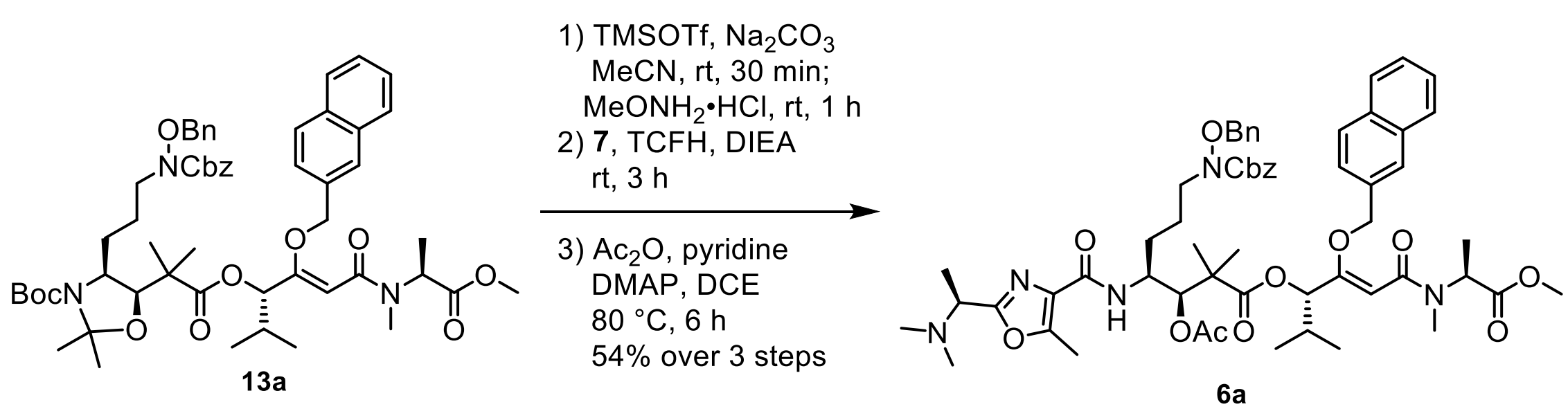

To a solution of **13a** (422 mg, 437 μmol, 1.0 equiv) in dry MeCN (2.2 mL) were added Na_2CO_3 (208 mg, 1.97 mmol, 4.5 equiv) and TMSOTf (237 μL, 1.31 mmol, 3.0 equiv) at room temperature under argon. After stirring at the same temperature for 30 min, *O*-methylhydroxylamine hydrochloride (36.5 mg, 437 μmol, 1.0 equiv) was added to the reaction mixture at the same temperature. After being stirred at the same temperature for 1 h, the mixture was diluted with MeCN and filtered through a pad of Celite®. The filtrate was concentrated in vacuo and the resulting residue was used for the next reaction without further purification.

To a suspension of the crude residue in dry MeCN (2.2 mL) were added **7** (113 mg, 480 μmol, 1.1 equiv) and TCFH (147 mg, 524 μmol, 1.2 equiv) at 0 °C under argon. After stirring at the same temperature for 10 min, DIEA (304 μL, 1.75 mmol, 4.0 equiv) was added to the reaction mixture at 0 °C, and then the

mixture was allowed to warm to room temperature. After being stirred at room temperature for 3 h, the mixture was quenched with saturated aqueous $NaHCO_3$ at 0 °C and the aqueous layer was extracted with ethyl acetate twice. The combined organic layers were washed with brine, dried with Na_2SO_4, and filtered. The filtrate was concentrated in vacuo. The residue was passed through a short-pad of silica-gel and the eluent was concentrated in vacuo. The resulting residue was used for the next reaction without further purification.

To a suspension of the crude residue in dry $(CH_2Cl)_2$ (2.2 mL) were added pyridine (141 μL, 1.75 mmol, 4.0 equiv), activated MS 3A (220 mg), acetic anhydride (82 μL, 873 μmol, 2.0 equiv) and DMAP (16 mg, 131 μmol, 0.3 equiv) at room temperature under argon. After being stirred at 80 °C for 6 h, the mixture was diluted with CH_2Cl_2 and filtered through a pad of Celite®. The filtrate was quenched with saturated aqueous $NaHCO_3$ at room temperature and the aqueous layer was extracted with ethyl acetate twice. The combined organic layers were washed with brine, dried with Na_2SO_4, and filtered. The filtrate was concentrated in vacuo. The residue was purified by flash column chromatography on amino silica-gel (eluted with hexane/EtOAc = 1:1) to afford acetate **6a** (249 mg, 238 μmol, 54% over 3 steps) as a colorless glass. $[\alpha]_D^{27} = -37$ (*c* 1.5, $CHCl_3$); ^{1}H NMR (600 MHz, $CDCl_3$, a mixture of rotamers) *δ* 7.70−7.83 (m, 4H), 7.43−7.50 (m, 3H), 7.24−7.43 (m, 10H), 7.06−7.11 (m, 1H), 5.14−5.33 (m, 6H), 5.03 (d, *J* = 12 Hz, 0.6H), 4.94 (d, *J* = 12 Hz, 0.4H), 4.76−4.86 (m, 2H), 4.55−4.65 (m, 2H), 4.32−4.40 (m, 1H), 3.60 −3.70 (m, 4H), 3.32−3.50 (m, 2H), 2.98 (s, 1H), 2.97 (s, 1.1H), 2.84 (s, 0.9H), 2.54−2.61 (m, 3H), 2.25 (s, 0.8H), 2.19 (s, 1.4H), 2.18 (s, 0.9H), 2.14 (s, 2.9H), 1.99−2.10 (m, 4H), 1.53−1.73 (m, 3H), 1.37−1.45 (m, 3H), 1.01−1.36 (m, 10H), 0.74−0.94 (m, 6H); ^{13}C{^{1}H} NMR (150 MHz, $CDCl_3$, a mixture of rotamers) *δ* 179.1, 175.3, 175.2, 172.3, 172.2, 171.8, 171.0, 170.3, 170.1, 167.7, 167.6, 167.5, 161.6, 161.4, 159.2, 159.1, 158.9, 158.8, 157.3, 157.2, 153.4, 136.2, 135.8, 135.3, 134.5, 134.4, 133.4, 133.3, 133.14, 133.11, 132.8, 129.5, 129.4, 128.7, 128.6, 128.4, 128.3, 128.1, 128.06, 128.05, 127.79, 127.77, 127.76, 126.7, 126.6, 126.5, 126.4, 126.2, 126.1, 126.1, 125.9, 125.7, 125.5, 98.7, 98.1, 80.9, 80.2, 80.1, 79.8, 79.18, 79.15, 79.0, 73.6, 73.3, 68.0, 67.7, 57.8, 56.5, 56.1, 53.5, 52.5, 52.3, 52.2, 52.0, 49.4, 48.0, 45.8, 44.0, 41.7, 32.9, 32.8, 32.0, 31.3, 30.5, 30.4, 30.3, 29.8, 29.7, 28.5, 28.4, 24.1, 23.49, 23.41, 22.6, 22.5, 20.9, 20.8, 20.0, 19.8, 18.9, 18.8, 17.8, 17.7, 15.2, 15.1, 15.0, 14.5, 14,4, 11.9; IR(neat): 2965, 1740, 1664, 1632, 1512, 1455, 1230, 752 cm^{-1}; HRMS(FAB) *m/z* calcd for $C_{58}H_{74}N_5O_{13}$ $[M+H]^+$ 1048.5278, found 1048.5261.

Oxazole analog **5a**

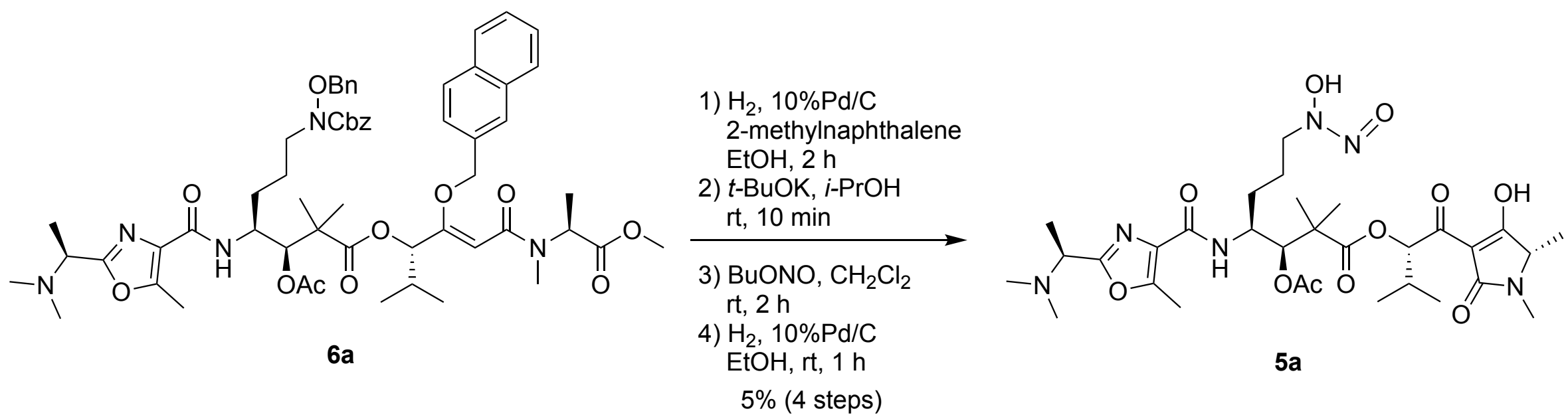

To a solution of **6a** (169 mg, 162 μmol, 1.0 equiv) in dry EtOH (3.2 mL) were added anhydrous 2-methylnaphthalene (27.6 mg, 194 μmol, 1.2 equiv) and 10% Pd/C (55 mg, 32wt%) at room temperature under argon. The reaction vessel was purged with hydrogen gas three times. After being stirred at room temperature for 2 h, the reaction mixture was filtered through a pad of Celite® and the filtrate was concentrated in vacuo. The residue was used for the next reaction without further purification.

To a suspension of the crude residues in dry *i*-PrOH (3.2 mL) was added *t*-BuOK (18.1 mg, 162 μmol, 1.0 equiv) at room temperature under argon. After being stirred at the same temperature for 10 min, the mixture was quenched with a phosphate buffer at 0 °C and the aqueous layer was extracted with ethyl acetate three times. The combined organic layers were washed with brine, dried with Na_2SO_4, and filtered. The filtrate was concentrated in vacuo. The resulting residue was used for the next reaction without further purification.

To a suspension of the crude residue in dry CH_2Cl_2 (2.0 mL) was added butyl nitrite (189 μL, 1.62 mmol, 10 equiv) at room temperature under argon. After being stirred at the same temperature for 1 h in dark, the mixture was concentrated in vacuo. The residue was passed through a short-pad of silica-gel and the eluent was concentrated in vacuo. Two-thirds of the residue was used for the next reaction without further purification.

To a suspension of the crude residue in dry EtOH (600 μL) was added 10% Pd/C (5 mg, 36wt%) at room temperature under argon. The reaction vessel was purged with hydrogen gas three times. After being stirred at the same temperature for 1 h, the reaction mixture was filtered through a pad of Celite®, and the filtrate was concentrated in vacuo. The residue was purified by MPLC (Duo 6g, eluted with H_2O/MeCN = 70:30) to afford **5a** (3.4 mg, 5.0 μmol, 5% over 4 steps) as a white amorphous solid. $[\alpha]_D^{25} = -22$ (c 0.50, CH_3OH); ^{1}H NMR (600 MHz, CD_3OD) δ 7.72 (d, J = 10.5 Hz, 0.6H), 7.67 (d, J = 10.5 Hz, 0.4H), 5.66 (d, J = 11.4 Hz, 1H), 5.58 (d, J = 9.0 Hz, 1H), 4.87−4.89 (m, 1H), 4.29−4.34 (m, 1H), 4.15−4.19 (m, 1H), 4.11−4.07 (m, 1H), 3.44−3.51 (m, 1H), 2.96 (s, 2.7H), 2.95 (s, 3.3H), 2.85 (s, 1.3H), 2.85 (s, 1.7H), 2.57 (s, 0.8H), 2.57 (s, 2.2H), 2.16−2.20 (m, 1H), 2.13 (s, 3.0H), 1.98 (brs, 1H), 1.82 (brs, 1H), 1.71 (d, J = 6.6 Hz, 1.2H), 1.70 (d, J = 1.8 Hz, 1H), 1.52−1.61 (m, 2H), 1.26 (d, J = 6.6 Hz, 1.6H), 1.25 (d, J = 6.6 Hz, 1.4H), 1.23 (s, 2.2H), 1.22 (s, 0.8H), 1.21 (s, 1H), 1.20 (s, 2H), 0.80−0.84 (m, 6H); $^{13}C\{^1H\}$ NMR (150

MHz, CD_3OD) δ 197.0, 196.9, 190.5, 177.0, 176.9, 175.1, 175.0, 172.3, 163.3, 163.2, 156.9, 156.8, 156.0, 155.9, 131.0, 130.9, 100.5, 81.8, 78.4, 78.4, 74.2, 62.4, 61.8, 59.3, 49.8, 49.7, 49.6, 47.0, 46.9, 44.1, 43.5, 41.4, 31.8, 31.8, 30.2, 30.2, 26.9, 26.7, 26.0, 24.0, 20.8, 20.3, 20.2, 18.1, 18.1, 16.7, 16.13, 16.10, 12.8, 12.8, 11.7, 11.65, 11.63; IR(neat): 3397, 1730, 1659, 1621, 1606, 1523, 1462, 1229 cm^{-1}; HRMS(FAB) *m/z* calcd for $C_{31}H_{49}N_6O_{11}$ $[M+H]^+$ 681.3454, found 681.3433.

Ester **13b**

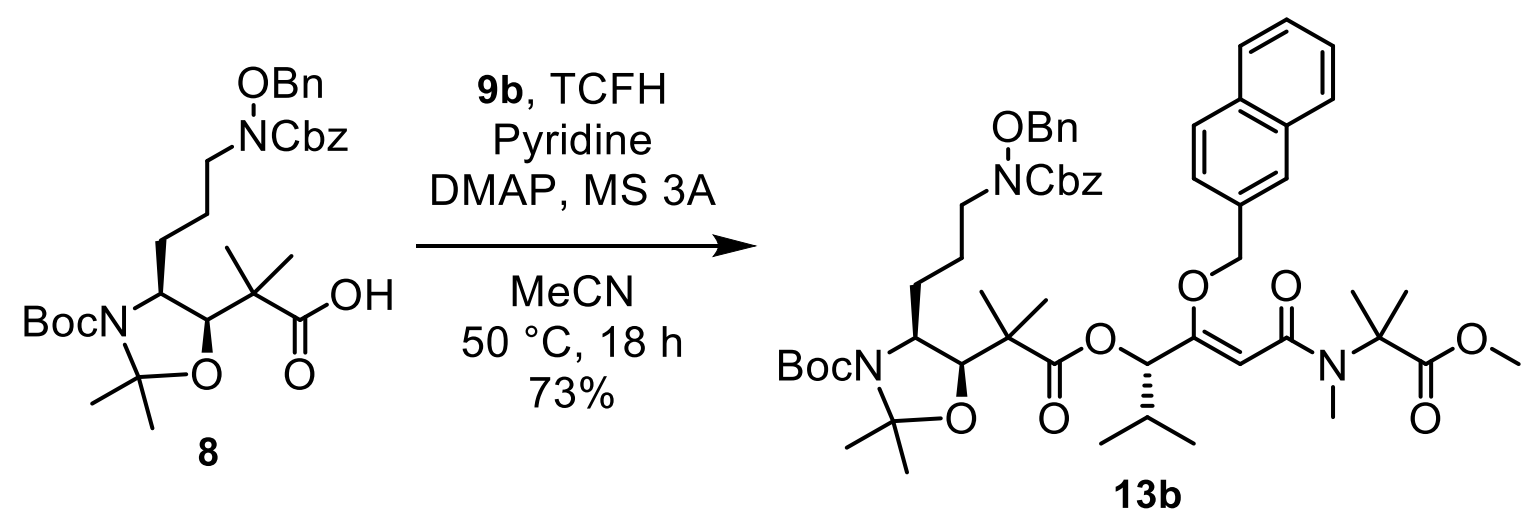

To a solution of **8** (356 mg, 608 μmol, 1.0 equiv) in dry MeCN (3.0 mL) were added alcohol **9b** (251 mg, 608 μmol, 1.0 equiv), pyridine (492 μL, 6.08 mmol, 10 equiv), activated MS 3A (300 mg), TCFH (341 mg, 1.22 mmol, 2.0 equiv) and DMAP (149 mg, 1.22 mmol, 2.0 equiv) at 0 °C under argon. After being stirred at 50 °C for 18 h, the mixture was diluted with ethyl acetate and the solution was filtered through a pad of Celite®. The filtrate was concentrated in vacuo. The residue was diluted with ethyl acetate and the solution was quenched with 10% aqueous citric acid at 0 °C. The aqueous layer was extracted with ethyl acetate twice. The combined organic layers were washed with 10% aqueous citric acid, saturated aqueous $NaHCO_3$ and brine twice, dried with $MgSO_4$, and filtered. The filtrate was concentrated in vacuo. The residue was purified by column chromatography on silica-gel (eluted with hexane/ethyl acetate = 2:1) to afford ester **13b** (435 mg, 444 μmol, 73%) as a pale-yellow amorphous solid. $[\alpha]_D^{26} = -30$ (*c* 1.4, $CHCl_3$); ^1H NMR (600 MHz, $CDCl_3$, a mixture of rotamers) δ 7.80−7.83 (m, 4H), 7.45−7.48 (m, 3H), 7.30−7.37 (m, 10H), 5.26 (d, *J* = 12.0 Hz, 1H), 5.24 (s, 1H), 5.17 (s, 1.7H), 5.17 (s, 0.3H), 4.97 (d, *J* = 12.0 Hz, 1H), 4.91 (d, *J* = 6.6 Hz, 1H), 4.83 (s, 1.7H), 4.81 (s, 0.3H), 4.19−4.21 (m, 0.5H), 4.10−4.13 (m, 1H), 4.01−4.02 (m, 0.5H), 3.67 (s, 0.6H), 3.66 (s, 2.4H), 3.47−3.56 (m, 1H), 3.36−3.45 (m, 1H), 3.03 (s, 1.3H), 3.02 (s, 1.3H), 2.84 (s, 0.4H), 2.14−2.20 (m, 1H), 1.64−1.68 (m, 2H), 1.52 (s, 1.2H), 1.52 (s, 1.8H), 1.47 (s, 3H), 1.35−1.44 (m, 17H), 1.30 (s, 1.2H), 1.29 (s, 1.4H), 1.26 (s, 0.7H), 1.24 (s, 1.2H), 1.21 (s, 1.1H), 1.20 (s, 0.2H), 1.17 (s, 0.2H), 0.94 (d, *J* = 7.2 Hz, 6H); $^{13}C\{^1H\}$ NMR (150 MHz, $CDCl_3$, a mixture of rotamers) δ 175.4, 175.2, 174.52, 174.46, 167.0, 166.9, 158.2, 157.1, 152.8, 152.0, 136.2, 136.1, 135.3, 134.6, 134.4, 133.2, 133.0, 129.3, 129.2, 128.51, 128.50, 128.43, 128.37, 128.32, 128.2, 128.14, 128.11, 128.0, 127.9, 127.7, 126.5, 126.4, 126.2, 126.12, 126.07, 126.0, 125.6, 99.7, 92.5, 92.1, 80.1, 79.9, 79.8, 79.7, 79.6, 77.1, 73.6, 73.5, 67.6, 67.5, 60.23, 60.18, 58.9, 58.4, 52.1, 49.9, 49.7, 44.00, 43.97, 43.8, 32.0, 30.4, 28.8, 28.7, 28.4, 28.3, 28.2, 27.4, 25.0, 24.8, 24.6, 24.3, 23.5, 23.2, 23.00, 22.96, 22.2, 21.2, 20.7,

19.0, 17.9; IR(neat): 2976, 1735, 1694, 1385, 1277, 1158, 1116 cm^{-1}; HRMS(EI) *m/z* calcd for $C_{56}H_{73}N_3O_{12}$ $[M]^+$ 979.5194, found 979.5208.

Amide **6b**

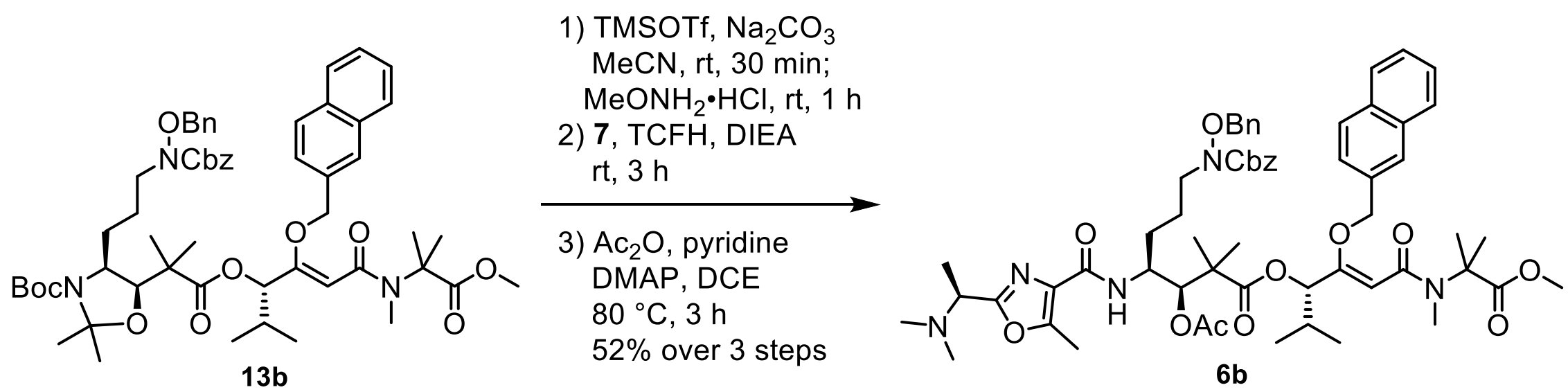

To a solution of **13b** (892 mg, 910 μmol, 1 equiv) in dry MeCN (4.6 mL) were added Na_2CO_3 (434 mg, 4.10 mmol, 4.5 equiv) and TMSOTf (493 μL, 2.73 mmol, 3.0 equiv) at room temperature under argon. After stirring at the same temperature for 30 min, *O*-methylhydroxylamine hydrochloride (76 mg, 910 μmol, 1.0 equiv) was added to the reaction mixture at the same temperature. After being stirred at the same temperature for 1 h, the mixture was diluted with MeCN and filtered through a pad of Celite®. The filtrate was concentrated in vacuo and the resulting residue was used for the next reaction without further purification.

To a suspension of the crude residue in dry MeCN (4.6 mL) were added **7** (235 mg, 1.00 mmol, 1.1 equiv) and TCFH (306 mg, 1.09 mmol, 1.2 equiv) at 0 °C under argon. After stirring at the same temperature for 10 min, DIEA (634 μL, 3.64 mmol, 4.0 equiv) was added to the reaction mixture at the same temperature, and then the mixture was allowed to warm to room temperature. After being stirred at the same temperature for 3 h, the mixture was quenched with saturated aqueous $NaHCO_3$ at 0 °C and the aqueous layer was extracted with ethyl acetate twice. The combined organic layers were washed with brine, dried with Na_2SO_4, and filtered. The filtrate was concentrated in vacuo. The residue was passed through a short-pad of silica-gel and the eluent was concentrated in vacuo. The resulting residue was used for the next reaction without further purification.

To a suspension of the crude residue in dry $(CH_2Cl)_2$ (4.6 mL) was added pyridine (294 μL, 3.64 mmol, 4.0 equiv), activated MS 3A (460 mg), acetic anhydride (172 μL, 1.82 mmol, 2.0 equiv) and DMAP (33 mg, 273 μmol, 0.3 equiv) at room temperature under argon. After being stirred at 80 °C for 3 h, the mixture was diluted with $CHCl_3$ and filtered through a pad of Celite®. The filtrate was quenched with saturated aqueous $NaHCO_3$ at room temperature and the aqueous layer was extracted with ethyl acetate twice. The combined organic layers were washed with brine, dried with Na_2SO_4, and filtered. The filtrate was concentrated in vacuo. The residue was purified by flash column chromatography on amino silica-gel (eluted with hexane/EtOAc = 1:1) to afford acetate **6b** (505 mg, 476 μmol, 52% over 3 steps) as a colorless

glass. $[\alpha]_D^{24}$ = −42 (*c* 1.2, $CHCl_3$); ^{1}H NMR (600 MHz, $CDCl_3$) δ 7.72−7.83 (m, 4H), 7.40−7.49 (m, 3H), 7.26−7.38 (m, 10H), 7.08 (d, *J* = 10.8 Hz, 1H), 5.03 (d, *J* = 7.2 Hz, 1H), 5.23 (d, *J* = 12.0 Hz, 1H), 5.17 (s, 1H, l), 5.16 (s, 1H, b'), 4.95 (d, *J* = 12.0 Hz, 1H, t), 4.80 (d, *J* = 10.2 Hz, 1H, j), 4.77 (d, *J* = 10.2 Hz, 1H), 4.61 (d, *J* = 6.6 Hz, 1H), 4.35−4.40 (m, 1H), 3.65−3.68 (m, 1H), 3.64 (s, 3H), 3.46−3.49 (m, 2H), 3.03 (s, 3H), 2.57 (s, 3H), 2.26 (s, 0.5H), 2.18 (s, 5.5H), 2.00−2.06 (m, 1H), 2.01 (s, 3H), 1.68−1.74 (m, 1H), 1.56−1.65 (m, 2H), 1.44 (s, 6H), 1.31 (d, *J* = 7.2 Hz, 3H), 1.25−1.31 (m, 1H), 1.25 (s, 3H), 1.14 (s, 3H), 0.80 (d, *J* = 6.6 Hz, 3H), 0.77 (d, *J* = 6.6 Hz, 3H); $^{13}C\{^1H\}$ NMR (150 MHz, $CDCl_3$) δ 175.1, 174.4, 170.8, 167.0, 161.5, 161.24, 161.17, 158.1, 157.0, 153.2, 136.1, 135.1, 134.4, 133.1, 133.0, 129.3, 128.5, 128.4, 128.3, 128.2, 128.1, 128.0, 127.9, 127.6, 126.5, 126.2, 126.1, 125.9, 125.6, 99.1, 80.0, 79.0, 77.1, 73.5, 67.6, 60.2, 57.7, 52.1, 49.2, 47.9, 47.8, 45.7, 43.8, 41.8, 41.6, 32.0, 30.3, 29.6, 23.4, 23.2, 23.0, 22.6, 20.6, 19.7, 18.6, 17.7, 14.9, 11.7; IR(neat): 2977, 1739, 1663, 1630, 1455, 1278, 1231, 1157, 1121, 1031, 752 cm^{-1}; HRMS(FAB) *m/z* calcd for $C_{59}H_{76}N_5O_{13}$ $[M+H]^+$ 1062.5434, found 1062.5431.

Oxazole analog **5b**

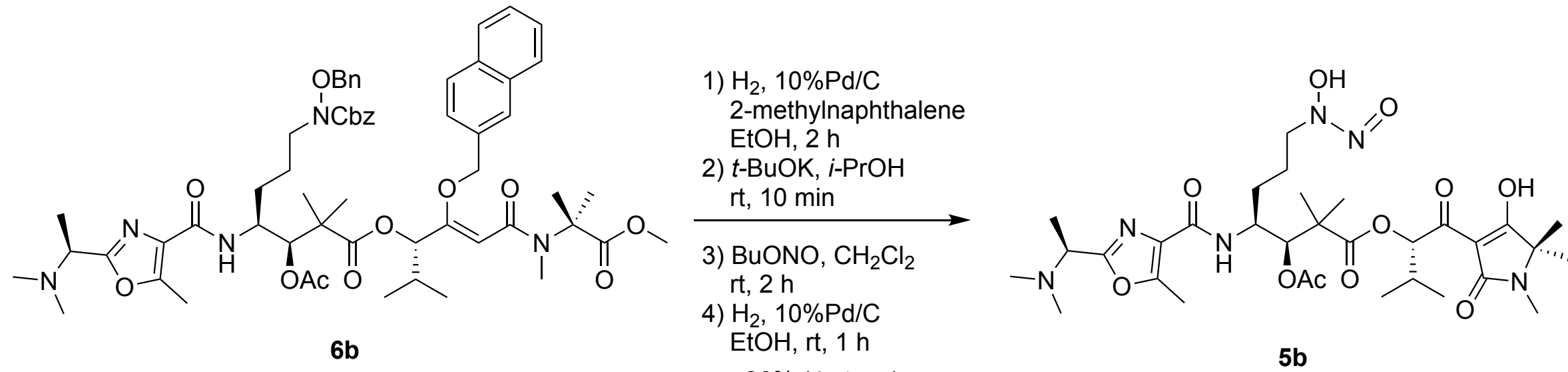

To a solution of **6b** (154 mg, 145 µmol, 1.0 equiv) in dry EtOH (2.9 mL) were added anhydrous 2-methylnaphthalene (24.8 mg, 174 µmol, 1.2 equiv) and 10% Pd/C (50 mg, 32wt%) at room temperature under argon. The reaction vessel was purged with hydrogen gas three times. After being stirred at room temperature for 2 h, the reaction mixture was filtered through a pad of Celite® and the filtrate was concentrated in vacuo. The residue was used for the next reaction without further purification.

To a suspension of the crude residue in dry *i*-PrOH (2.9 mL) was added *t*-BuOK (16.3 mg, 145 µmol, 1.0 equiv) at room temperature under argon. After being stirred at the same temperature for 10 min, the mixture was added phosphate buffer at 0 °C and extracted with ethyl acetate three times. The combined organic layers were washed with brine, dried with Na_2SO_4, filtered, and the filtrate was concentrated in vacuo. The resulting residue was used for the next reaction without further purification.

To a suspension of the crude residue in dry CH_2Cl_2 (1.8 mL) was added butyl nitrite (170 µL, 1.45 mmol, 10 equiv) at room temperature under argon. After being stirred for 2 h at the same temperature in dark, the mixture was concentrated in vacuo. The residue was passed through short-pad silica-gel and the eluent

was concentrated in vacuo. The half of resulting residue was used for the next reaction without further purification.

To a suspension of the crude residue in dry EtOH (700 µL) was added 10% Pd/C (6 mg, 36wt%) at room temperature under argon and the reaction vessel was purged with hydrogen gas three times. After being stirred at room temperature for 1 h, the reaction mixture was filtered through a pad of Celite®, and the filtrate was concentrated in vacuo. The residue was purified by MPLC (Duo 6g, eluted with H_2O/MeCN = 70:30) and afforded **5b** (11.8 mg, 17.0 µmol, 26% over 4 steps) as a white amorphous solid. $[\alpha]_D^{25}$ = −24 (*c* 0.40, CH_3OH); ^{1}H NMR (600 MHz, CD_3OD) δ 7.83 (d, 1H, *J* = 6.0 Hz), 5.80 (s, 1H), 5.59 (d, *J* = 9.0 Hz, 1H), 4.49 (brs, 1H), 4.32 (d, *J* = 9.7 Hz, 0.5H), 4.31 (d, *J* = 9.7 Hz, 0.5H), 4.16 (m, 1H), 4.09 (m, 1H), 2.80 (s, 3H), 2.71 (s, 6H), 2.53 (s, 3H), 2.18 (m, 1H), 2.14 (s, 3H), 1.99 (m, 1H), 1.84 (m, 1H), 1.58 (d, *J* = 7.2 Hz, 3H), 1.50−1.59 (m, 2H), 1.22 (s, 3H), 1.20 (s, 3H), 1.18 (s, 3H), 1.17 (s, 3H), 0.87 (d, *J* = 6.9 Hz, 3H), 0.85 (d, *J* = 6.9 Hz, 3H); ^{13}C{^{1}H} NMR (150 MHz, CD_3OD) δ 199.9, 191.0, 176.8, 174.1, 172.5, 163.6, 159.0, 155.5, 130.7, 98.9, 81.9, 79.0, 64.0, 62.3, 59.0, 49.9, 47.1, 41.5, 31.9, 30.1, 25.7, 24.0, 22.6, 20.8, 20.4, 18.5, 16.6, 13.4, 11.6; IR(neat): 2970, 1737, 1657, 1610, 1524, 1467, 1375, 1229, 1149, 1033 cm^{-1}; HRMS(FAB) *m/z* calcd for $C_{32}H_{51}N_6O_{11}$ $[M+H]^+$ 695.3610, found 695.3597.

Amide **15a**

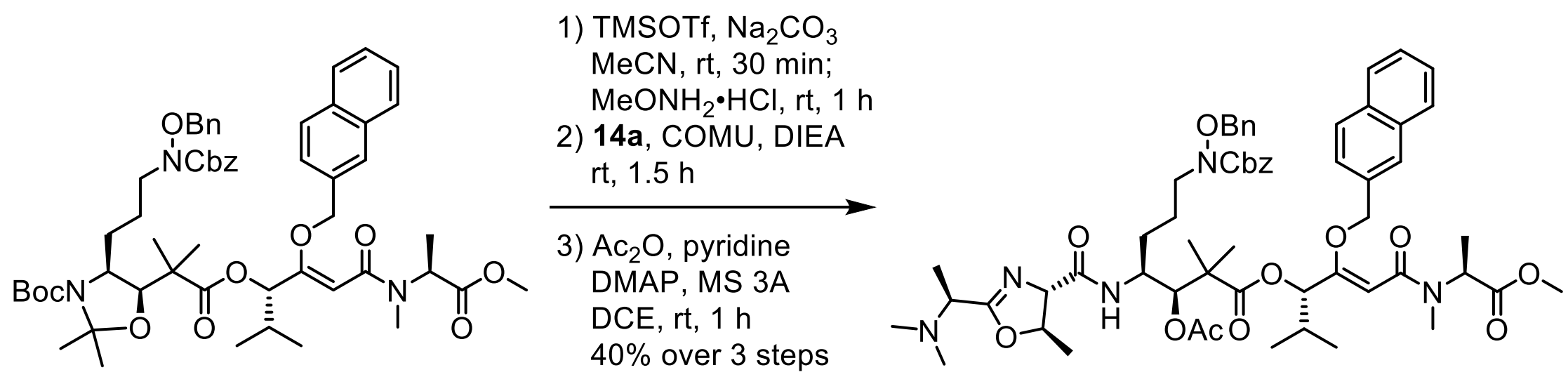

To a solution of **13a** (172 mg, 178 µmol, 1.0 equiv) in dry MeCN (890 µL) were added Na_2CO_3 (85 mg, 800 µmol, 4.5 equiv) and TMSOTf (96 µL, 533 µmol, 3.0 equiv) at room temperature under argon. After stirring at the same temperature for 30 min, *O*-methylhydroxylamine hydrochloride (15 mg, 178 µmol, 1.0 equiv) was added to the reaction mixture at the same temperature. After being stirred at the same temperature for 1 h, the mixture was diluted with MeCN and filtered through a pad of Celite®. The filtrate was concentrated in vacuo and the resulting residue was used for the next reaction without further purification.

To a suspension of the crude residue and **14a** (44.1 mg, 220 µmol, 1.2 equiv) in dry MeCN (890 µL) was added COMU (107 mg, 249 µmol, 1.4 equiv) at room temperature under argon. After stirring at the same temperature for 10 min, DIEA (62 µL, 356 µmol, 2.0 equiv) was added to the reaction mixture. After being stirred at the same temperature for 1 h, the mixture was quenched with saturated aqueous $NaHCO_3$ at 0 °C and the aqueous layer was extracted with ethyl acetate twice. The combined organic layers were washed with brine, dried with Na_2SO_4, and filtered. The filtrate was concentrated in vacuo. The residue was passed

through a short-pad of silica-gel and the eluent was concentrated in vacuo. The resulting residue was used for the next reaction without further purification.

To a suspension of the crude residue in dry $(CH_2Cl)_2$ (890 μL) were added pyridine (58 μL, 711 μmol, 4.0 equiv), activated MS 3A (90 mg), acetic anhydride (34 μL, 356 μmol, 2.0 equiv) and DMAP (7 mg, 53.3 μmol, 0.3 equiv) at room temperature under argon. After being stirred at the same temperature for 1 h, the mixture was diluted with EtOAc and filtered through a pad of Celite®. The filtrate was quenched with saturated aqueous $NaHCO_3$ at room temperature and the aqueous layer was extracted with ethyl acetate twice. The combined organic layers were washed with brine, dried with Na_2SO_4, and filtered. The filtrate was concentrated in vacuo. The residue was purified by flash column chromatography on silica-gel (eluted with $CHCl_3$/MeOH = 19:1) then preparative TLC (3% MeOH in $CHCl_3$) to afford acetate **15a** (74.5 mg, 70.9 μmol, 40% over 3 steps) as a colorless glass. $[\alpha]_D^{25} = -14$ (*c* 1.2, $CHCl_3$); ^{1}H NMR (600 MHz, $CDCl_3$, a mixture of rotamers) *δ* 7.78−7.86 (m, 4H), 7.42−7.49 (m, 3H), 7.30−7.39 (m, 10H), 6.90−6.95 (m, 1H), 4.89−5.36 (m, 7H), 4.74−4.85 (m, 2H), 4.66 (quint, *J* = 6.6 Hz, 1H), 4.23 (brs, 1H), 4.07 (d, *J* = 6.6 Hz, 0.5H), 4.05 (d, *J* = 6.6 Hz, 0.5H), 3.70 (s, 0.4H), 3.69 (s, 0.3H), 3.67 (s, 0.5H), 3.66 (s, 0.8H), 3.65 (s, 1H), 3.37−3.45 (m, 2H), 3.32 (q, *J* = 6.6 Hz, 1H), 3.01 (s, 0.9H), 3.00 (s, 1H), 2.83−2.91 (m, 1.1H), 2.26 (s, 4H), 2.25 (s, 1H), 2.25 (s, 1H), 2.14−2.21 (m, 1H), 1.98 (s, 1.5H), 1.97 (s, 1.5H), 1.47−1.55 (m, 3H), 1.38−1.42 (m, 6H), 1.05−1.32 (m, 10H), 0.93−0.97 (m, 6H); $^{13}C\{^1H\}$ NMR (150 MHz, $CDCl_3$, a mixture of rotamers) *δ* 174.8, 174.7, 172.2, 172.1, 171.6, 171.6, 171.0, 171.0, 169.7, 167.5, 167.4, 167.2, 167.2, 159.4, 159.3, 159.1, 159.1, 156.9, 156.9, 136.0, 135.1, 134.4, 134.3, 134.3, 133.1, 133.0, 133.0, 129.3, 128.5, 128.4, 128.2, 128.1, 128.0, 128.0, 127.9, 127.6, 126.6, 126.4, 126.2, 126.1, 126.1, 126.1, 126.0, 126.0, 125.6, 125.5, 125.5, 98.7, 98.5, 80.1, 80.1, 80.0, 79.8, 79.8, 79.8, 79.7, 79.7, 77.2, 74.6, 74.6, 73.5, 67.6, 57.9, 56.4, 56.3, 52.2, 52.1, 51.8, 48.9, 47.9, 45.9, 45.8, 45.8, 41.8, 41.7, 32.8, 32.6, 30.6, 30.6, 30.5, 30.5, 29.6, 29.1, 29.0, 28.4, 28.3, 23.2, 21.9, 21.9, 21.9, 21.9, 21.6, 20.5, 18.9, 18.0, 17.9, 15.1, 14.9, 14.9, 14.9, 14.9, 14.4, 14.3; IR(neat): 2973, 1739, 1679, 1650, 1513, 1454, 1372, 1225, 1141, 1034, 752, 699 cm^{-1}; HRMS(FAB) *m/z* calcd for $C_{58}H_{76}N_5O_{13}$ $[M+H]^+$ 1050.5434, found 1050.5426.

JBIR-141 (**1a**)

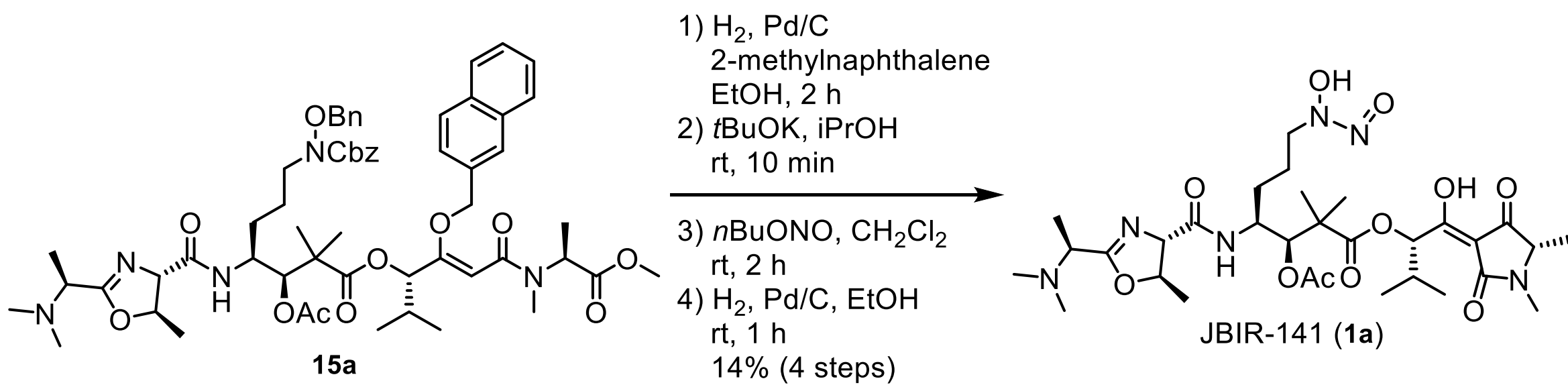

To a solution of **15a** (90.6 mg, 86.3 μmol, 1.0 equiv) in dry EtOH (1.7 mL) were added anhydrous 2-methylnaphthalene (14.7 mg, 104 μmol, 1.2 equiv) and 10% Pd/C (30 mg, 33wt%) at room temperature under argon. The reaction vessel was purged with hydrogen gas three times. After being stirred at the same temperature for 2 h, the reaction mixture was filtered through a pad of Celite® and the filtrate was concentrated in vacuo. The residue was used for the next reaction without further purification.

To a suspension of the crude residue in dry *i*-PrOH (1.7 mL) was added *t*-BuOK (9.7 mg, 86.3 μmol, 1.0 equiv) at room temperature under argon. After being stirred at the same temperature for 10 min, the mixture was quenched with a phosphate buffer at 0 °C and the aqueous layer was extracted with ethyl acetate three times. The combined organic layers were washed with brine, dried with Na_2SO_4, and filtered. The filtrate was concentrated in vacuo. The resulting residue was used for the next reaction without further purification.

To a suspension of the crude residue in dry CH_2Cl_2 (1.1 mL) was added butyl nitrite (101 μL, 863 μmol, 10 equiv) at room temperature under argon. After being stirred at the same temperature for 2 h in dark, the mixture was concentrated in vacuo. The residue was passed through a short-pad of silica-gel and the eluent was concentrated in vacuo. The resulting residue was used for the next reaction without further purification.

To a suspension of the crude residue in dry EtOH (1.0 mL) was added 10% Pd/C (6 mg, 30wt%) at room temperature under argon. The reaction vessel was purged with hydrogen gas three times. After being stirred at the same temperature for 1 h, the reaction mixture was filtered through a pad of Celite® and the filtrate was concentrated in vacuo. The residue was purified by MPLC (Duo 6g, eluted with H_2O/MeCN = 70:30) and lyophilized. Then the residue was passed through a short-pad of silica-gel (eluted with $CHCl_3$/MeOH = 4:1) and the eluent was concentrated in vacuo to afford JBIR-141 (**1a**) (8.3 mg, 12 μmol, 14% over 4 steps) as a white amorphous solid. Before short-pass $[\alpha]_D^{24}$ = +54 (*c* 0.30, CH_3OH); After short-pass $[\alpha]_D^{24}$ = +96 (*c* 0.30, CH_3OH) [lit.[4] $[\alpha]_D^{23}$ = +90 (*c* 0.2, CH_3OH)]; ^1H NMR (600 MHz,

[4] Kawahara, T.; Kagaya, N.; Masuda, Y.; Doi, T.; Izumikawa, M.; Ohta, K.; Hirao, A.; Shin-ya, K. *Org. Lett.* **2015**, *17*, 5476–5479.

CD_3OD) and $^{13}C\{^1H\}$ NMR (150 MHz, CD_3OD): See section 1.3 (page 28); IR(neat): 2972, 1731, 1671, 1620, 1523, 1474, 1227 cm^{-1}; HRMS(ESI) *m/z* calcd for $C_{31}H_{49}N_6O_{11}$ $[M-H]^-$ 681.3465, found 681.3448.

Amide **15b**

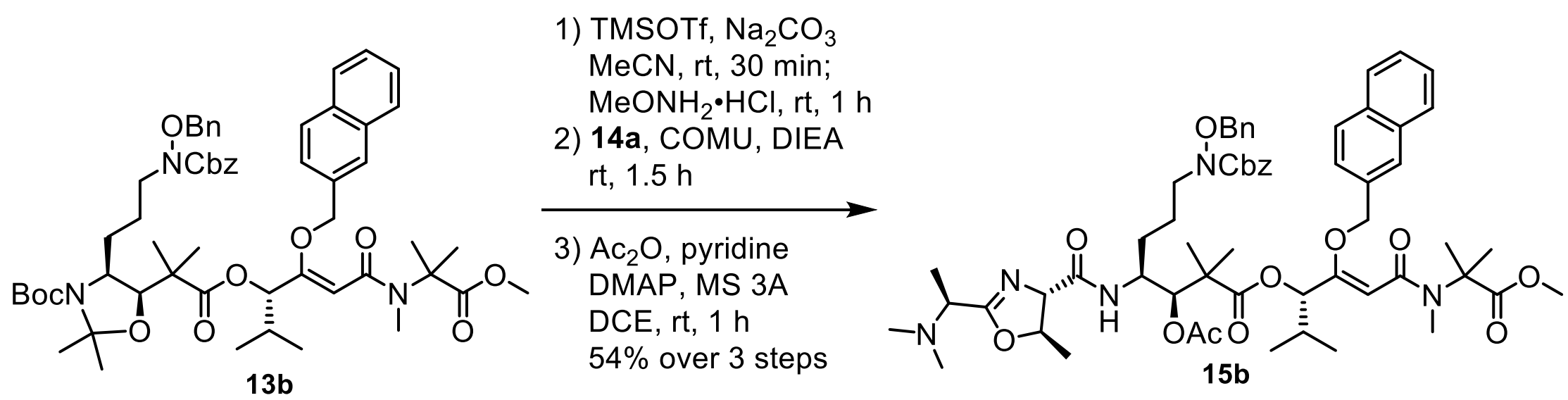

To a solution of **13b** (257 mg, 263 μmol, 1.0 equiv) in dry MeCN (1.3 mL) were added Na_2CO_3 (125 mg, 1.18 mmol, 4.5 equiv) and TMSOTf (142 μL, 788 μmol, 3.0 equiv) at room temperature under argon. After stirring at the same temperature for 30 min, *O*-methylhydroxylamine hydrochloride (21.9 mg, 263 μmol, 1.0 equiv) was added to the reaction mixture at the same temperature. After being stirred at the same temperature for 1 h, the mixture was diluted with MeCN and filtered through a pad of Celite®. The filtrate was concentrated in vacuo and the resulting residue was used for the next reaction without further purification.

To a suspension of the crude residue and **14a** (68.3 mg, 341 μmol, 1.3 equiv) in dry MeCN (1.3 mL) was added COMU (157 mg, 367.5 μmol, 1.4 equiv) at room temperature under argon. After stirring at the same temperature for 10 min, DIEA (92 μL, 525 μmol, 2.0 equiv) was added to the reaction mixture. After being stirred at the same temperature for 1 h, the mixture was quenched with saturated aqueous $NaHCO_3$ at 0 °C and the aqueous layer was extracted with ethyl acetate twice. The combined organic layers were washed with brine, dried with Na_2SO_4, and filtered. The filtrate was concentrated in vacuo. The residue was passed through a short-pad of silica-gel and the eluent was concentrated in vacuo. The resulting residue was used for the next reaction without further purification.

To a suspension of the crude residue in dry $(CH_2Cl)_2$ (1.3 mL) was added pyridine (85 μL, 1.05 mmol, 4.0 equiv), activated MS 3A (130 mg), acetic anhydride (50 μL, 525 μmol, 2.0 equiv) and DMAP (9.6 mg, 78.6 μmol, 0.3 equiv) at room temperature under argon. After being stirred at room temperature for 1 h, the mixture was diluted with EtOAc and filtered through a pad of Celite®. The filtrate was quenched with saturated aqueous $NaHCO_3$ at room temperature and the aqueous layer was extracted with ethyl acetate twice. The combined organic layers were washed with brine, dried with Na_2SO_4, and filtered. The filtrate was concentrated in vacuo. The residue was purified by flash column chromatography on silica-gel (eluted with $CHCl_3$/MeOH = 19:1) to afford acetate **15b** (151.6 mg, 142.4 μmol, 54% over 3 steps) as a colorless oil. $[\alpha]_D^{27} = -9.7$ (*c* 1.2, $CHCl_3$); ^1H NMR (600 MHz, $CDCl_3$, a mixture of rotamers) δ 7.80−7.85 (m, 4H,), 7.43−7.49 (m, 3H), 7.29−7.38 (m, 10H), 6.91 (d, *J* = 10.2 Hz, 1H), 5.26 (d, *J* = 11.7 Hz, 1H), 5.23 (s, 1H),

5.17 (s, 2H), 5.12 (d, J = 5.4 Hz, 0.3H), 5.11 (d, J = 5.4 Hz, 0.7H), 4.97 (d, J = 11.7 Hz, 1H), 4.91 (d, J = 6.6 Hz, 1H), 4.78 (d, J = 3.0 Hz, 1H), 4.63−4.67 (m, 1H), 4.20−4.24 (m, 1H), 4.04−4.07 (m, 1H), 3.67 (m, 0.5H), 3.65 (s, 0.3H), 3.65 (s, 2.2H), 3.41 (t, J = 6.3 Hz, 2H), 3.30 (brs, 1H), 3.05 (s, 2.7H), 2.89 (s, 0.3H), 2.25 (s, 6H), 2.13−2.18 (m, 1H), 1.97 (s, 3H), 1.51−1.55 (m, 3H), 1.45 (s, 6H), 1.41 (d, J = 6.0 Hz, 3H), 1.23−1.31 (m, 3H), 1.20 (s, 3H), 1.14 (s, 3H), 1.06−1.12 (m, 1H), 0.94 (d, J = 7.2 Hz, 3H), 0.93 (d, J = 7.2 Hz, 3H); $^{13}C\{^1H\}$ NMR (150 MHz, $CDCl_3$, a mixture of rotamers) δ 174.9, 174.8, 174.4, 171.0, 171.0, 166.9, 158.3, 156.9, 136.0, 135.1, 134.4, 133.1, 133.0, 129.3, 128.5, 128.4, 128.2, 128.0, 127.9, 127.7, 126.4, 126.2, 126.0, 125.5, 99.5, 80.0, 79.8, 79.7, 77.0,74.6, 74.6, 73.5, 67.6, 60.2, 57.9, 57.9, 52.1, 48.9, 47.9, 45.8, 41.7, 32.0, 30.6, 29.1, 29.0, 23.2, 23.1, 23.1, 21.9, 21.9, 21.7, 21.7, 20.6, 20.5, 20.4, 18.9, 18.0, 14.9, 14.9; IR(neat): 2975, 2938, 1738, 1679, 1649, 1279, 1230, 1157, 1119, 1031 cm^{-1}; HRMS(FAB) *m/z* calcd for $C_{59}H_{78}N_5O_{13}$ $[M+H]^+$ 1064.5591, found 1064.5608.

25-Methyl-JBIR-141 (**1b**)

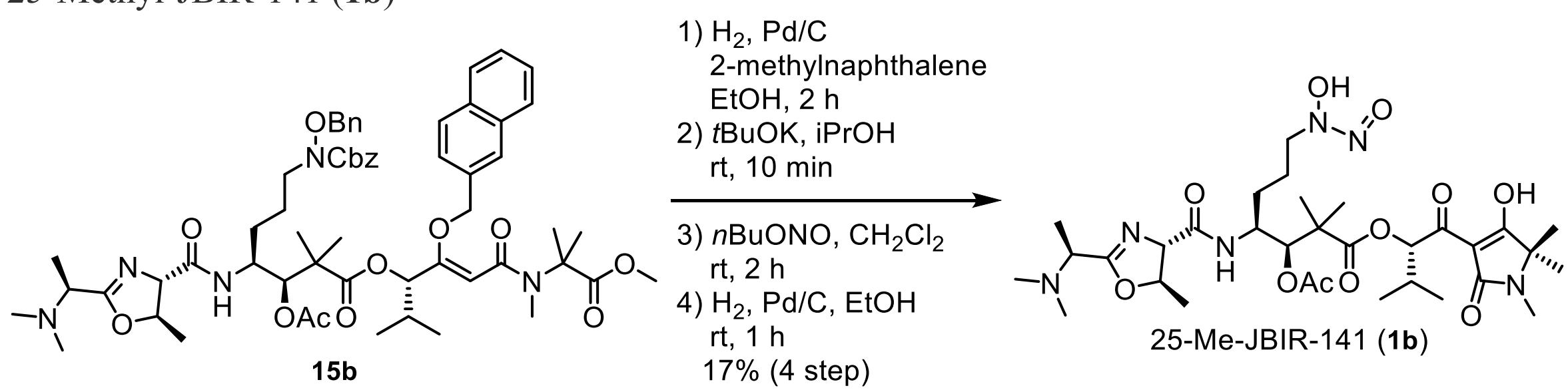

To a solution of **15b** (100.0 mg, 94.0 μmol, 1.0 equiv) in dry EtOH (1.9 mL) were added anhydrous 2-methylnaphthalene (16.0 mg, 112.8 μmol, 1.2 equiv) and 10% Pd/C (35 mg, 30wt%) at room temperature under argon. The reaction vessel was purged with hydrogen gas three times. After being stirred at the same temperature for 2 h, the reaction mixture was filtered through a pad of Celite® and the filtrate was concentrated in vacuo. The residue was used for the next reaction without further purification.

To a suspension of the crude residue in dry *i*-PrOH (1.9 mL) was added *t*-BuOK (10.5 mg, 94.0 μmol, 1.0 equiv) at room temperature under argon. After being stirred at the same temperature for 10 min, the mixture was quenched with a phosphate buffer at 0 °C and the aqueous layer was extracted with ethyl acetate three times. The combined organic layers were washed with brine, dried with Na_2SO_4, and filtered. The filtrate was concentrated in vacuo. The resulting residue was used for the next reaction without further purification.

To a suspension of the crude residue in dry CH_2Cl_2 (1.2 mL) was added butyl nitrite (110 μL, 940 μmol, 10 equiv) at room temperature under argon. After being stirred at the same temperature for 2 h in dark, the mixture was concentrated in vacuo. The residue was passed through a short-pad of silica-gel and the eluent was concentrated in vacuo. Two-thirds of the resulting residue was used for the next reaction without further purification.

To a suspension of the crude residue in dry EtOH (600 μL) was added 10% Pd/C (6 mg, 36wt%) at room temperature under argon. The reaction vessel was purged with hydrogen gas three times. After being stirred at the same temperature for 1 h, the reaction mixture was filtered through a pad of Celite® and the filtrate was concentrated in vacuo. The residue was purified by MPLC (Duo 6g, eluted with H_2O/MeCN = 70:30) and lyophilized. Then the residue passed through a short-pad of silica-gel (eluted with $CHCl_3$/MeOH = 4:1) and the eluent was concentrated in vacuo to afford 25-Me-JBIR-141 (**1b**) (7.4 mg, 11 μmol, 17% over 4 steps) as a white amorphous solid. $[\alpha]_D^{24}$ = +130 (*c* 0.40, CH_3OH); Only major signals are listed. ^{1}H NMR (600 MHz, CD_3OD) δ 7.86 (d, *J* = 10.8 Hz, 1H), 6.17 (d, *J* = 3.0 Hz, 1H), 5.56 (d, *J* = 10.2 Hz, 1H), 4.85 (brs, 1H), 3.88−3.95 (m, 3H), 3.69 (d, *J* = 4.8 Hz, 1H), 3.42−3.46 (m, 1H), 2.85 (s, 3H), 2.35 (s, 6H), 2.24−2.14 (m, 1H), 2.14 (s, 1H), 1.68−1.81 (m, 3H), 1.31−1.38 (m, 3H), 1.27−1.31 (m, 1H), 1.22 (s, 3H), 1.19 (s, 3H), 1.12 (s, 3H), 1.09 (d, *J* = 6.0 Hz, 3H), 1.06 (d, *J* = 6.9 Hz, 3H), 1.03 (d, *J* = 6.9 Hz, 3H), 1.02 (s, 3H); ^{13}C{^{1}H} NMR (150 MHz, CD_3OD) δ 200.5, 192.8, 177.0, 172.8, 172.3, 172.2, 172.1, 100.1, 80.6, 80.4, 78.9, 74.9, 64.9, 59.7, 57.6, 50.8, 47.1, 42.7, 33.1, 30.5, 26.2, 23.7, 22.7, 22.5, 22.3, 20.8, 20.7, 20.6, 17.1, 16.7, 15.4; IR(neat): 2968, 1734, 1672, 1630, 1615, 1478, 1224 cm^{-1}; HRMS(ESI) *m/z* calcd for $C_{32}H_{52}KN_6O_{11}$ $[M+K]^+$ 735.3326, found 735.3328.

Amide **15c**

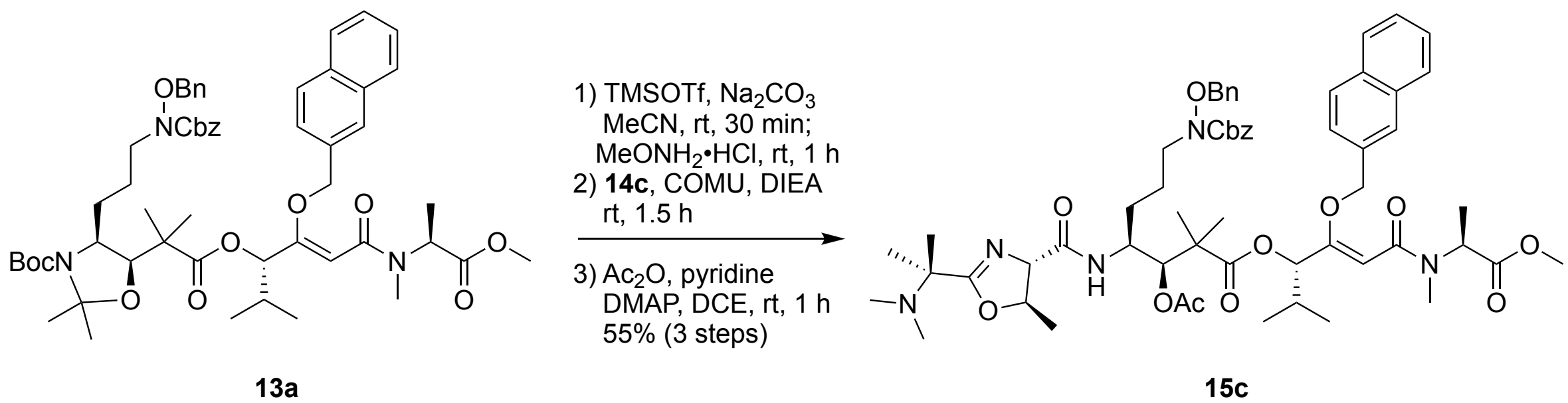

To a solution of **13a** (298 mg, 308 μmol, 1.0 equiv) in dry MeCN (1.5 mL) were added Na_2CO_3 (147 mg, 1.39 mmol, 4.5 equiv) and TMSOTf (167 μL, 925 μmol, 3.0 equiv) at room temperature under argon. After stirring at the same temperature for 30 min, *O*-methylhydroxylamine hydrochloride (25.8 mg, 308 μmol, 1.0 equiv) was added to the reaction mixture at the same temperature. After being stirred at the same temperature for 1 h, the mixture was diluted with MeCN and filtered through a pad of Celite®. The filtrate was concentrated in vacuo and the resulting residue was used for the next reaction without further purification.

To a suspension of the crude residue and **14b** (78.2 mg, 365 μmol, 1.2 equiv) in dry MeCN (1.5 mL) was added COMU (172 mg, 401 μmol, 1.3 equiv) at room temperature under argon. After stirring at the same temperature for 10 min, DIEA (118 μL, 679 μmol, 2.2 equiv) was added to the reaction mixture. After being stirred at the same temperature for 1 h, the mixture was quenched with saturated aqueous $NaHCO_3$ at 0 °C and the aqueous layer was extracted with ethyl acetate twice. The combined organic layers were

washed with brine, dried with Na_2SO_4, and filtered. The filtrate was concentrated in vacuo. The residue was passed through a short-pad of silica-gel and the eluent was concentrated in vacuo. The resulting residue was used for the next reaction without further purification.

To a suspension of the crude residue in dry $(CH_2Cl)_2$ (1.6 mL) were added pyridine (100 μL, 1.23 mmol, 4.0 equiv), acetic anhydride (58 μL, 617 μmol, 2.0 equiv) and DMAP (11 mg, 92.5 μmol, 0.3 equiv) at room temperature under argon. After being stirred at the same temperature for 1 h, the mixture was diluted with EtOAc and filtered through a pad of Celite®. The filtrate was quenched with saturated aqueous $NaHCO_3$ at room temperature and the aqueous layer was extracted with ethyl acetate twice. The combined organic layers were washed with brine, dried with Na_2SO_4, and filtered. The filtrate was concentrated in vacuo. The residue was purified by flash column chromatography on silica-gel (eluted with $CHCl_3$/MeOH = 19:1) then preparative TLC (3% MeOH in EtOAc) to afford acetate **15c** (180 mg, 169 μmol, 55% over 3 steps) as a colorless glass. $[\alpha]_D^{12} = -13$ (*c* 0.80, $CHCl_3$, a mixture of rotamers); ^{1}H NMR (600 MHz, $CDCl_3$) δ 7.72−7.86 (m, 4H), 7.40−7.49 (m, 3H), 7.30−7.37 (m, 10H), 6.92−6.97 (m, 1H), 4.84−5.35 (m, 7H), 4.90 (d, J = 9.6 Hz, 1H), 4.77 (d, J = 9.6 Hz, 1H), 4.66 (quint, J = 6.3 Hz, 1H), 4.20−4.22 (m, 1H), 4.04−4.07 (m, 1H), 3.62−3.70 (m, 3H), 3.37−3.43 (m, 2H), 3.00 (s, 0.9H), 2.99 (s, 1H), 2.86 (s, 1.1H), 2.24 (s, 2H), 2.23 (s, 2H), 2.21 (s, 2H), 2.15−2.20 (m, 1H), 1.92−2.06 (m, 1H), 1.49−1.54 (m, 3H), 1.37−1.46 (m, 6H), 1.29−1.33 (m, 6H), 1.02−1.27 (m, 6H), 0.91−1.01 (m, 6H); ^{13}C NMR (150 MHz, $CDCl_3$, a mixture of rotamers) δ 175.1, 174.9, 173.1, 172.2, 172.1, 171.6, 171.1, 171.0, 167.5, 167.5, 167.2, 159.1, 159.0, 156.9, 136.1, 135.2, 134.5, 134.4, 134.3, 133.2, 133.0, 133.0, 129.4, 128.6, 128.5, 128.4, 128.2, 128.2, 128.1, 128.0, 128.0, 127.8, 127.7, 126.5, 126.4, 126.4, 126.3, 126.2, 126.1, 126.1, 126.0, 126.0, 125.9, 125.6, 125.5, 98.9, 98.5, 80.3, 80.2, 80.2, 80.0, 79.9, 79.7, 74.6, 73.5, 73.2, 67.6, 67.5, 58.2, 56.4, 52.4, 52.2, 52.1, 51.8, 48.9, 47.8, 47.8, 45.8, 45.8, 45.7, 43.9, 39.1, 39.1, 32.8, 32.6, 30.6, 30.5, 30.4, 29.7, 29.4, 29.3, 28.4, 28.3, 23.3, 23.2, 22.6, 22.6, 22.5, 22.0, 21.9, 21.8, 21.8, 21.7, 21.6, 21.5, 20.6, 20.5, 20.5, 20.1, 18.9, 18.9, 18.1, 18.1, 18.0, 17.9, 15.2, 15.1, 14.4, 14.3; IR(neat): 2975, 2940, 1739, 1679, 1649, 1510, 1455, 1372, 1226, 1136, 1032, 752 cm^{-1}; HRMS[FAB] m/z calcd for $C_{59}H_{78}N_5O_{13}$ $[M+H]^+$ for 1064.5591, found 1064.5604.

17-Methyl-JBIR-141 (**1c**)

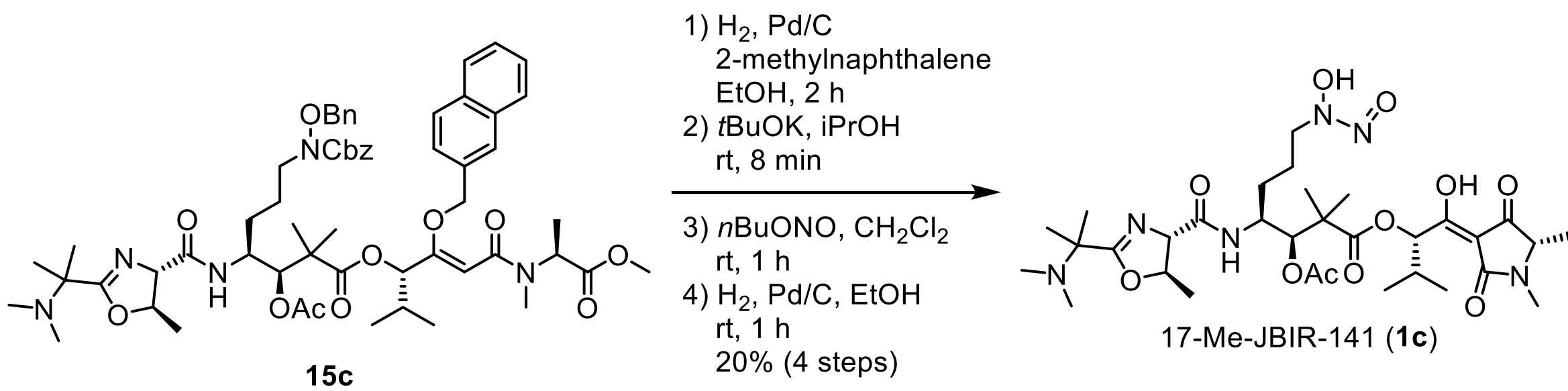

To a solution of **15c** (176 mg, 165 μmol, 1.0 equiv) in dry EtOH (3.3 mL) was added anhydrous 2-methylnaphthalene (23.5 mg, 165 μmol, 1.0 equiv) and 10% Pd/C (60 mg, 34wt%) at room temperature under argon. The reaction vessel was purged with hydrogen gas three times. After being stirred at the same temperature for 2 h, the reaction mixture was filtered through a pad of Celite® and the filtrate was concentrated in vacuo. The residue was used for the next reaction without further purification.

To a suspension of the crude residue in dry *i*-PrOH (3.8 mL) was added *t*-BuOK (18.6 mg, 165 μmol, 1.0 equiv) at room temperature under argon. After being stirred at the same temperature for 10 min, the mixture was quenched with a phosphate buffer at 0 °C and the aqueous layer was extracted with ethyl acetate three times. The combined organic layers were washed with brine, dried with Na_2SO_4, and filtered. The filtrate was concentrated in vacuo. The resulting residue was used for the next reaction without further purification.

To a suspension of the crude residue in dry CH_2Cl_2 (2.1 mL) was added butyl nitrite (193 μL, 1.65 mmol, 10 equiv) at room temperature under argon. After being stirred at the same temperature for 2 h in dark, the mixture was concentrated in vacuo. The residue was passed through a short-pad of silica-gel and the eluent was concentrated in vacuo. Half of the resulting residue was used for the next reaction without further purification.

To a suspension of the crude residue in dry EtOH (700 μL) was added 10% Pd/C (7 mg, 38wt%) at room temperature under argon. The reaction vessel was purged with hydrogen gas three times. After being stirred at the same temperature for 1 h, the reaction mixture was filtered through a pad of Celite® and the filtrate was concentrated in vacuo. The residue was purified by MPLC (Duo 6g, eluted with H_2O/MeCN = 80:20) and lyophilized. Then the residue was passed through a short-pad of silica-gel (eluted with $CHCl_3$/MeOH = 4:1) and the eluent was concentrated in vacuo to afford 17-Me-JBIR-141 (**1c**) (11.1 mg, 15.9 μmol, 67%) as a white amorphous solid. $[\alpha]_D^{12} = +95$ (*c* 0.29, CH_3OH); ^1H NMR (600 MHz, CD_3OD, only major peaks are listed.) δ 7.70 (d, $J = 11.4$ Hz, 1H), 6.20 (d, $J = 1.8$ Hz, 1H), 5.55 (d, $J = 9.9$ Hz, 1H), 4.72−4.75 (m, 1H), 4.06−4.07 (m, 1H), 3.93 (q, $J = 9.9$ Hz, 1H), 3.80−3.84 (m, 1H), 3.77 (d, $J = 4.2$ Hz, 1H), 3.54−3.59 (m, 1H), 2.90 (s, 3H), 2.33 (s, 6H), 2.11−2.20 (m, 1H), 2.13 (s, 3H), 1.91−1.97 (m, 1H), 1.70−1.75 (m, 1H), 1.60−1.67 (m, 1H), 1.37 (s, 3H), 1.35 (s, 3H), 1.26−1.34 (m, 1H), 1.24 (d, $J = 6.6$ Hz, 3H), 1.12 (s, 3H),1.09 (d, $J = 6.6$ Hz, 3H), 1.05 (d, $J = 7.2$ Hz, 3H), 1.04 (s, 3H), 1.01 (d, $J = 7.2$ Hz, 3H);

^{13}C-NMR (150 MHz, CD_3OD, only major peaks are listed.) δ 197.6, 193.0, 176.9, 175.2, 174.0, 172.8, 172.2, 101.3, 80.9, 80.5, 79.0, 75.2, 62.4, 60.1, 57.4, 50.9, 47.2, 39.7, 33.5, 29.9, 26.7, 26.4, 22.1, 22.0, 21.3, 20.9, 20.9, 20.8, 17.2, 16.4, 16.1; IR(neat): 2971, 1735, 1671, 1632, 1521, 1486, 1227 cm^{-1}; HRMS[ESI] *m/z* calcd for $C_{32}H_{53}N_6O_{11}$ $[M+H]^+$ for 697.3767, found 697.3757.

1.3 Comparison of NMR spectral data of the natural product and synthetic JBIR-141 (**1a**)

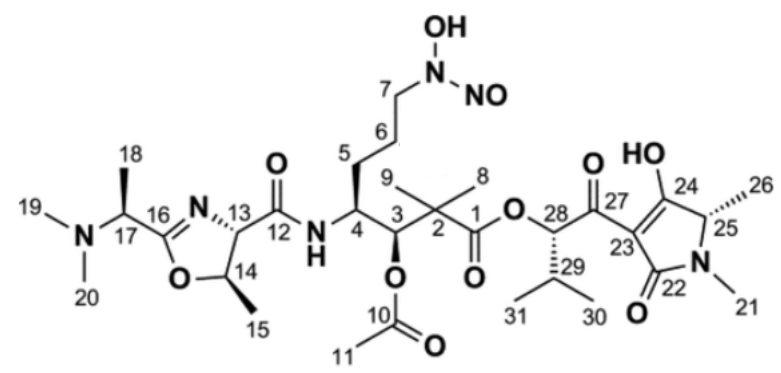

	Natural product		Synthetic **1a**		chemical shift Δ	
	δ ^{1}H^{a}	δ ^{13}C^{b}	δ ^{1}H^{c}	δ ^{13}C^{d}	δ ^{1}H	δ ^{13}C
1	-	177.0*	-	177.0	-	0.0
2	-	47.2	-	47.2	-	0.0
3	5.56	79.0	5.57	79.0	0.01	0.0
4	3.93	50.9	3.93	50.8	0.00	0.1
5	1.77, 1.26	30.5	1.76, 1.28	30.5	-0.01, 0.02	0.0
6	1.75, 1.75	22.6	1.76, 1.76	22.6	0.01, 0.01	0.0
7	3.95, 3.90	57.5	3.93, 3.90	57.5	-0.02, 0.00	0.0
8	1.13	26.2	1.12	26.2	-0.01	0.0
9	1.03	17.1	1.02	17.1	-0.01	0.0
10	-	172.2	-	172.2	-	0.0
11	2.14	20.8	2.14	20.8	0.00	0.0
12	-	172.2	-	172.2	-	0.0
13	3.68	75.0	3.68	75.0	0.00	0.0
14	4.90	80.5	4.95	80.5	0.05	0.0
15	1.11	20.9	1.10	20.9	-0.01	0.0
16	-	172.4	-	172.4	-	0.0
17	3.41	59.8	3.43	59.8	0.02	0.0
18	1.34	15.6	1.33	15.6	-0.01	0.0
19/20	2.34	42.8	2.33	42.8	-0.01	0.0
21	2.91	26.6	2.90	26.7	-0.01	0.01
22	-	173.9	-	174.0	-	0.1
23	-	101.4	-	101.4	-	0.0
24	-	197.8	-	197.8	-	0.0
25	3.55	62.4	3.57	62.4	0.02	0.0
26	1.25	16.0	1.25	15.9	0.00	0.1
27	-	192.6	-	192.6	-	0.0
28	6.15	80.5	6.14	80.5	0.01	0.0
29	2.18	33.1	2.17	33.1	-0.01	0.0
30	1.05	16.7	1.04	16.7	-0.01	0.0
31	1.06	20.6	1.06	20.6	0.00	0.0

[a]Recorded at 500 MHz in CD_3OD. [b]Recorded at 125 MHz in CD_3OD. [c]Recorded at 600 MHz in CD_3OD. [d]Recorded at 150 MHz in CD_3OD.

1.4 Comparison of acid stability of **1a** and **1c**

JBIR-141 (**1a**) and **1c** were separately dissolved in 50% aqueous MeCN containing 1% formic acid, respectively. After stirring for 1 h at room temperature, each reaction mixture was analyzed by UPLC–MS (Figure S1). The oxazoline **1a** underwent hydrolysis,[1] whereas the 17-methyl derivative **1c** remained.

(a) Acid treatment of **1a**

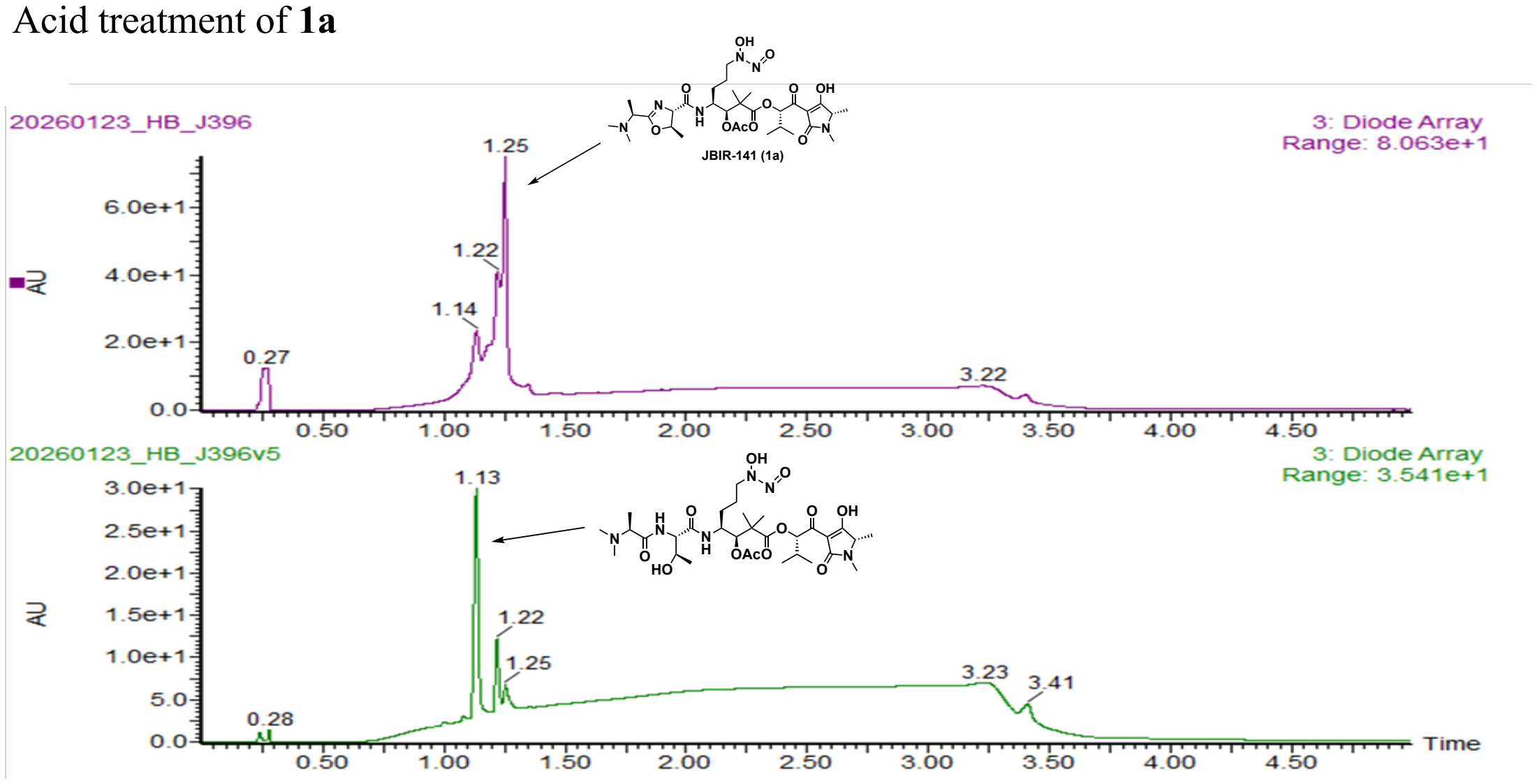

(b) Acid treatment of **1c**

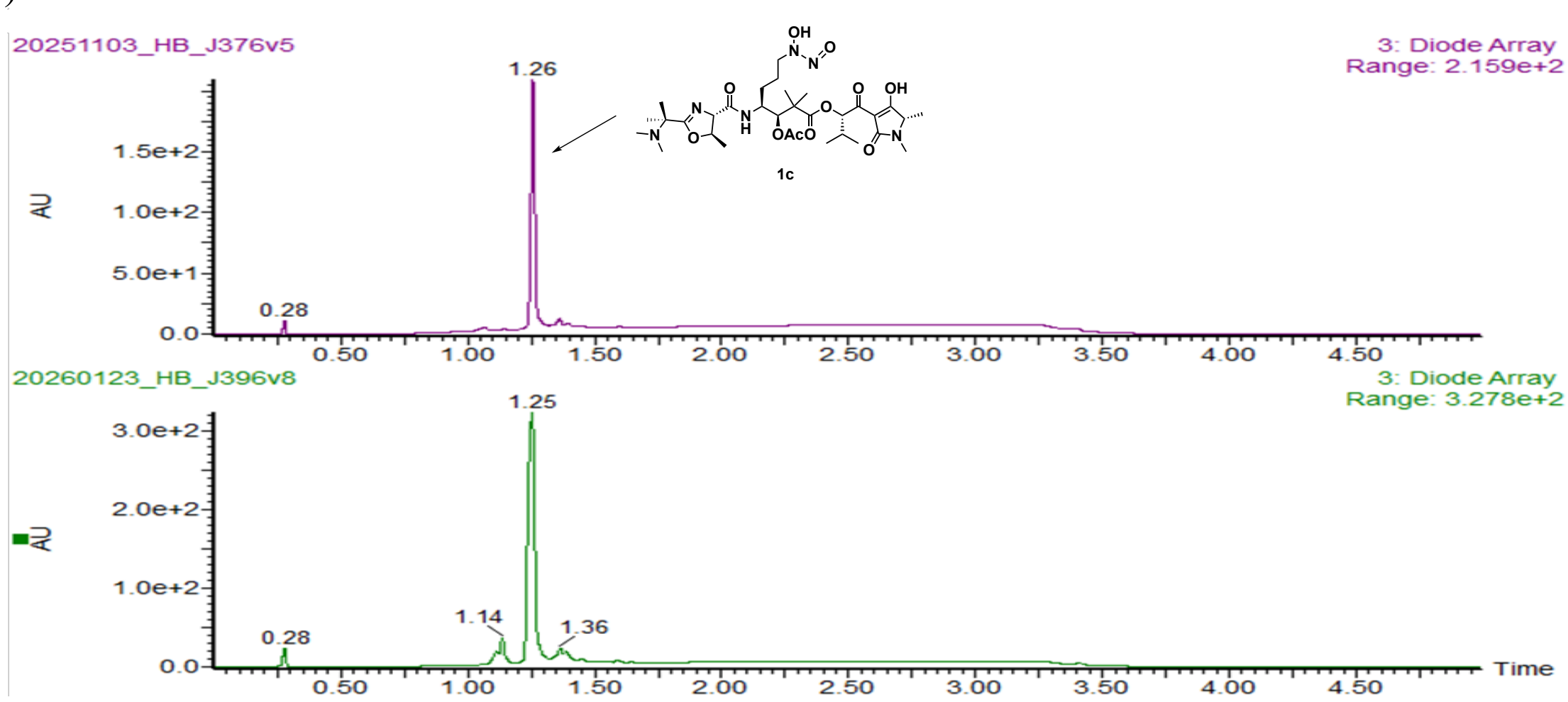

Figure S1. UPLC analysis of **1a** (a) and **1c** (b) before (top) and after (bottom) acid treatment for 1 h. Each reaction mixture was analyzed by reversed-phase UPLC using a Waters ACQUITY UPLC H-Class PLUS System equipped with a photodiode array detector. Analysis was performed on an ACQUITY UPLC BEH C18 column (1.7 µm, 2.1 x 50 mm, Waters) with a flow rate of 0.5 mL/min using a gradient method as follows: 10–90% B (0.00–1.11 min), 90% B (1.11–2.50 min), 90–10% B (2.50–3.53 min), and 10% B (3.53-5.00 min), A: 0.1% HCO_2H/H_2O and B: 0.1% HCO_2H/MeCN.

1.5 Biological evaluation

1.5.1 FOXO3 reporter assay and cell viability assay in HEK293 cells

HEK293 cells were maintained in DMEM medium (FUJIFILM Wako, Japan) with 10% fetal bovine serum (Gibco, USA) and penicillin (50 U/mL)-streptomycin (50 µg/mL) (Gibco, USA) at 37 °C in a humidified incubator with 5% CO_2. To assess the FOXO3 inhibitory activities of compounds, cells were transfected with FOXO3 plasmid (pcDNA3-Flag-FOXO3, pGL3-Basic-6 × DBE) using Lipofectamine 2000 (Invitrogen, USA). After overnight incubation, transfected cells were harvested and seeded in 384-well plate at a density of 4,000 cells/30 µL/well. After 4 h, cells were treated with different concentrations of compounds for 24 h. The vehicle solvent (DMSO) was used as a control. Luciferase reporter activities were measured using PicaGene BrillianStar-LT Luciferase Assay System (TOYO B-Net, Japan) and Envision Multilabel Plate Reader (Revvity, USA) (Figure S2, top). Cell viabilities were measured using CellTiter-Glo Luminescent Cell Viability Assay (Promega, USA) and Envision Multilabel Plate Reader (Figure S2, bottom).

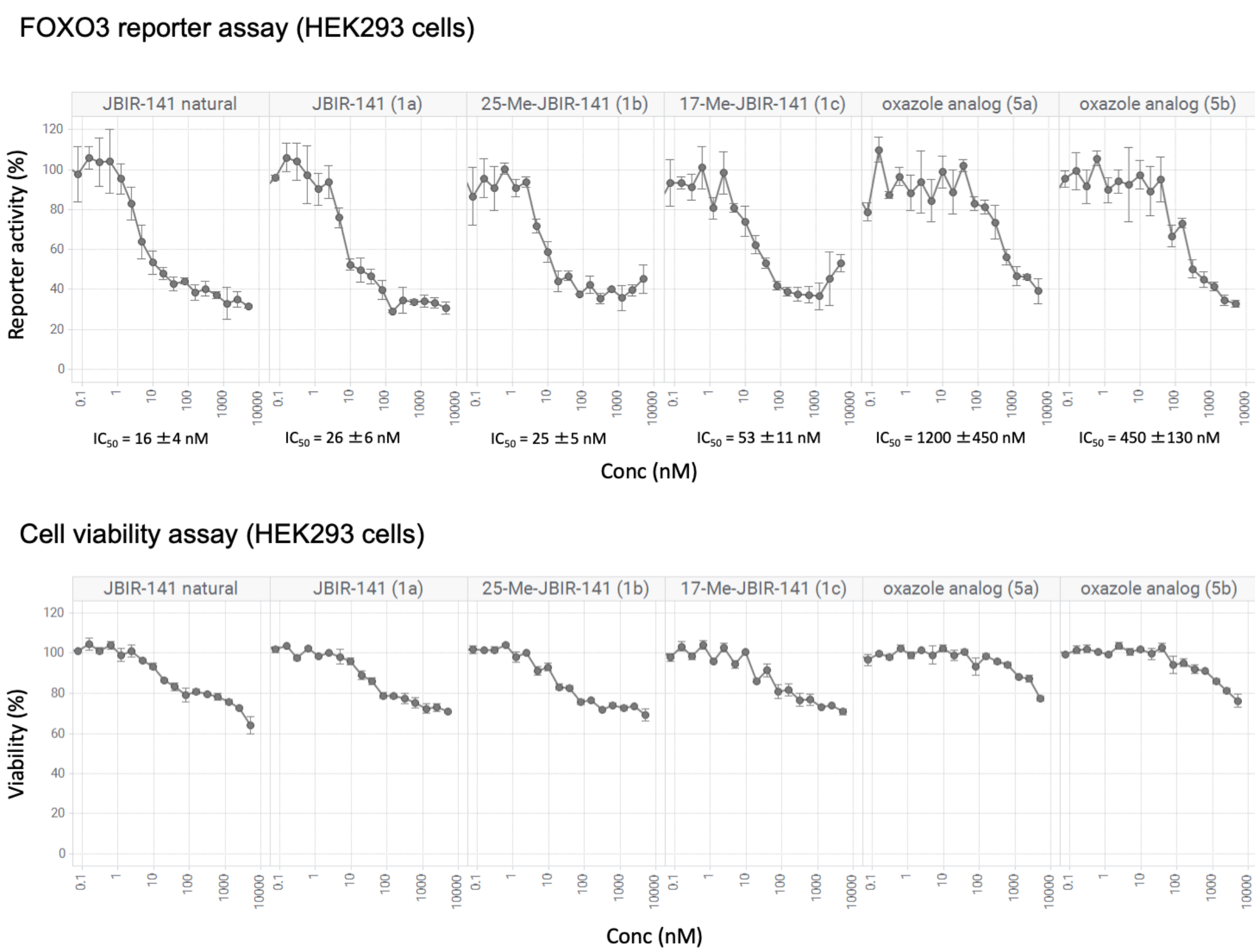

Figure S2. Dose-response curves for FOXO3 inhibition (top) and cell viability (bottom) after 24 h incubation with natural JBIR-141, synthetic JBIR-141 (**1a**), 25-Me-JBIR-141 (**1b**), 17-Me-JBIR-141 (**1c**), and oxazole analog **5a** and **5b**.

1.5.2 Cytotoxicity assay against Jurkat cells

Jurkat cells (obtained from Cell Resource Center for Biomedical Research, Institute of Development, Aging and Cancer, Tohoku University) were maintained in Dulbecco's Modified Eagle Medium (nacalai tesque) containing 10% fetal bovine serum (gibco) and 1% antibiotic (penicillin-streptomycin mixed solution, nacalai tesque) at 37 °C under 5% CO_2. For the purpose of the experiment, 2 x 10^4 cells suspended in 100 μL of medium per well were plated in 96-well plate and incubated at 37 °C under 5% CO_2. After incubation for 24 h, a solution of compound in DMSO (1 μL, concentration: 0.001, 0.01, 0.1, 1, 10 μM, respectively) was added to the above-mentioned well, resulting in various concentrations of the compound (0.01, 0.1, 1, 10, 100 nM) or solvent control (1% DMSO). After additional incubation for 72 h under the same conditions, 5 μL of WST-8 (nacalai tesque) was added to the cell culture. After the cell culture was further incubated for 4 h, optical density at 450 nm (with an optical reference wavelength at 650 nm) was measured with a microplate reader. All assays were performed in triplicate to confirm reproducibility (Figure S3).

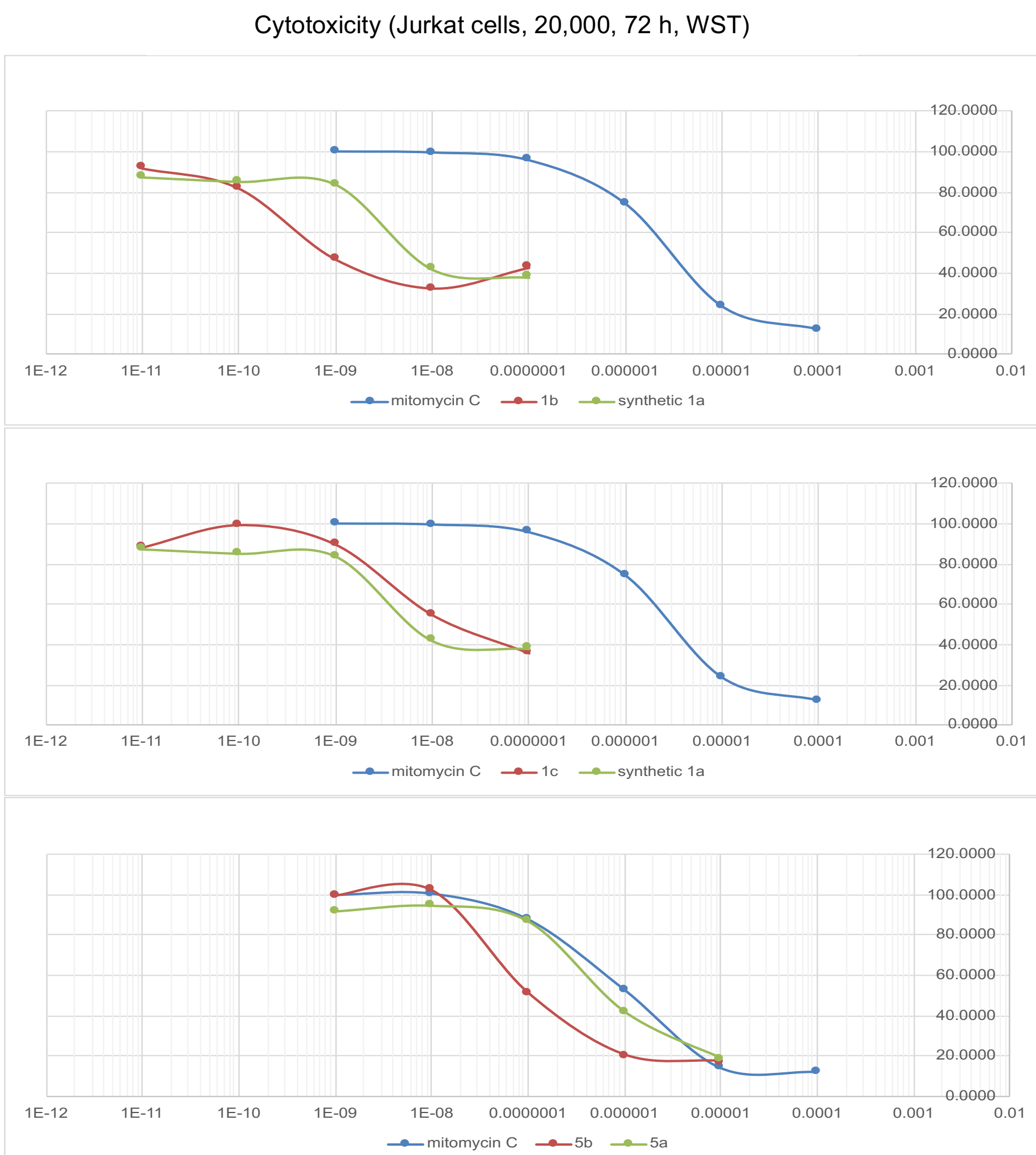

Figure S3. Dose-response curves for cytotoxicity of synthetic compounds **1a**, **1b**, **1c**, **5a**, and **5b** against Jurkat cells. Mitomycin C was used as a standard.

^{1}H NMR spectrum of compound **S1** (400 MHz, $CDCl_3$)

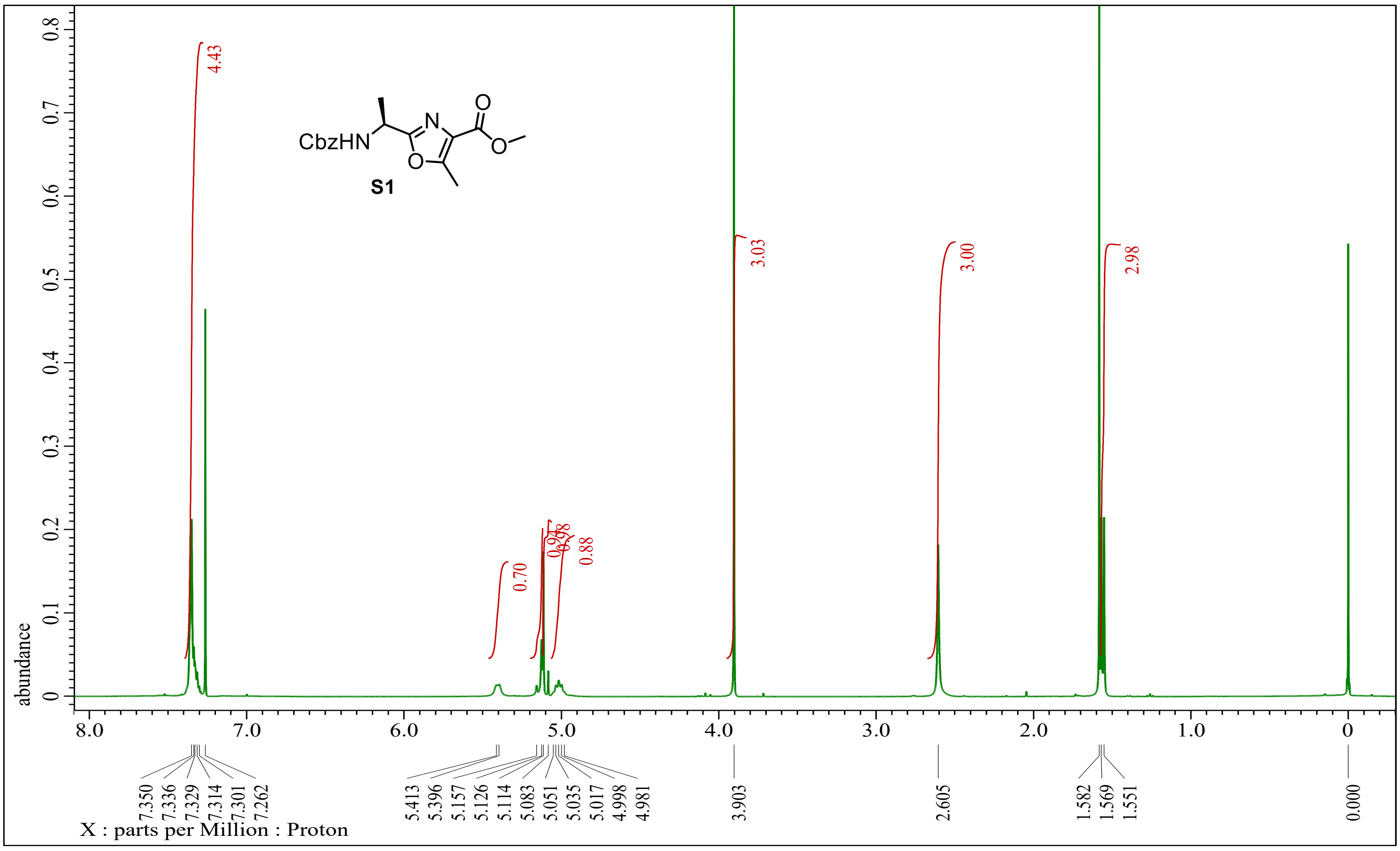

^{13}C NMR spectrum of compound **S1** (100 MHz, $CDCl_3$)

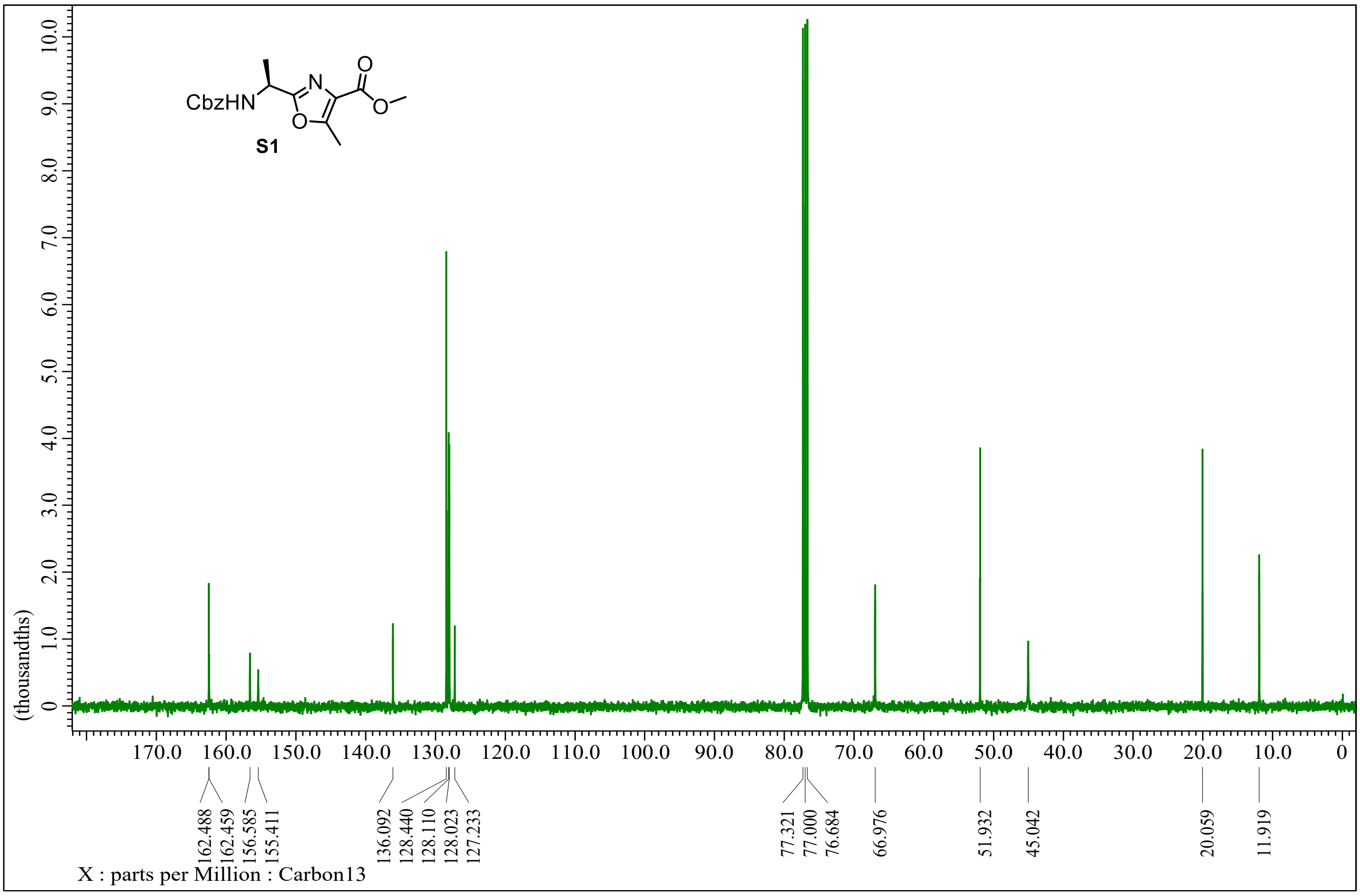

^{1}H NMR spectrum of compound **S2** (400 MHz, $CDCl_3$)

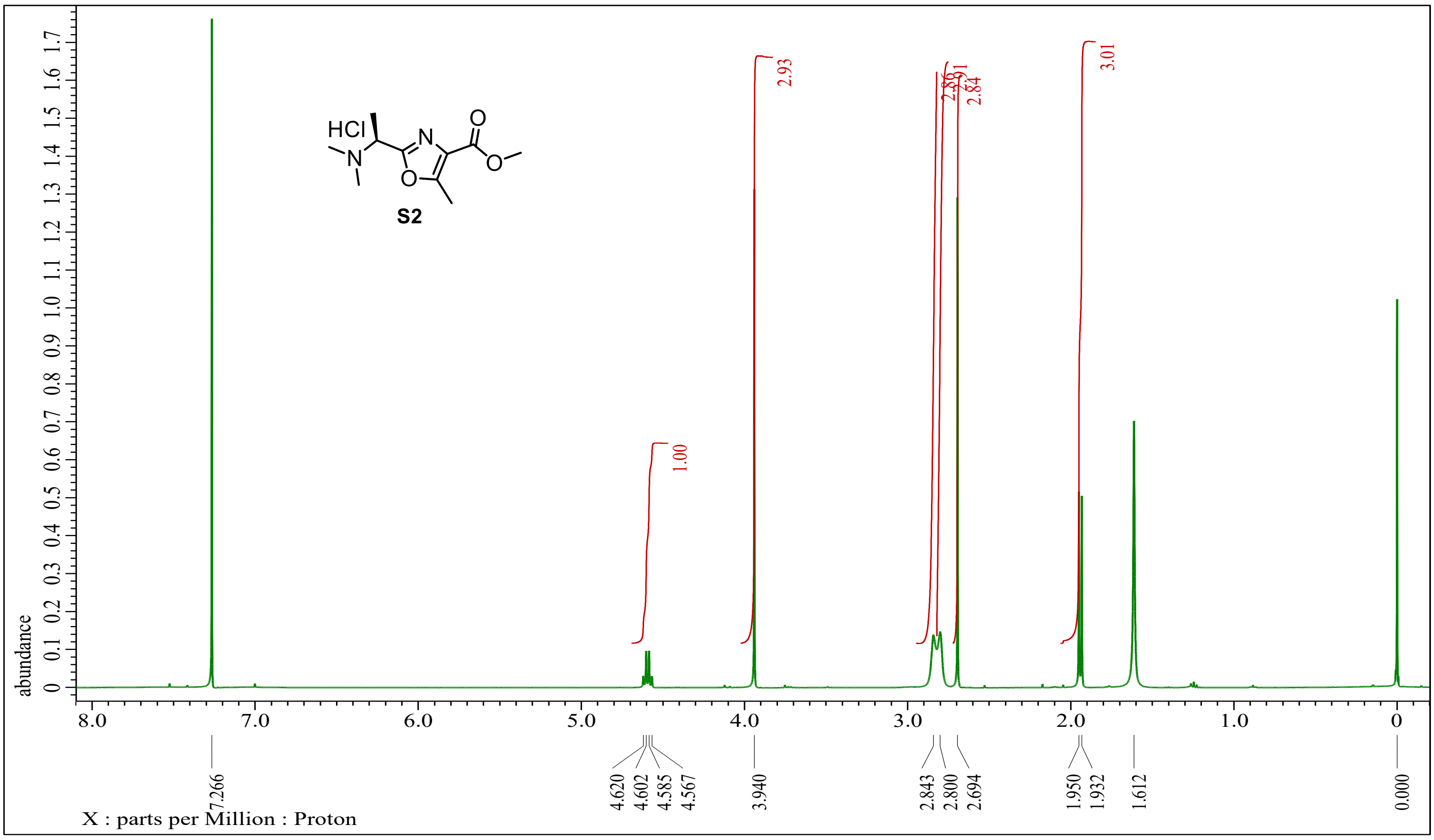

^{13}C NMR spectrum of compound **S2** (100 MHz, $CDCl_3$)

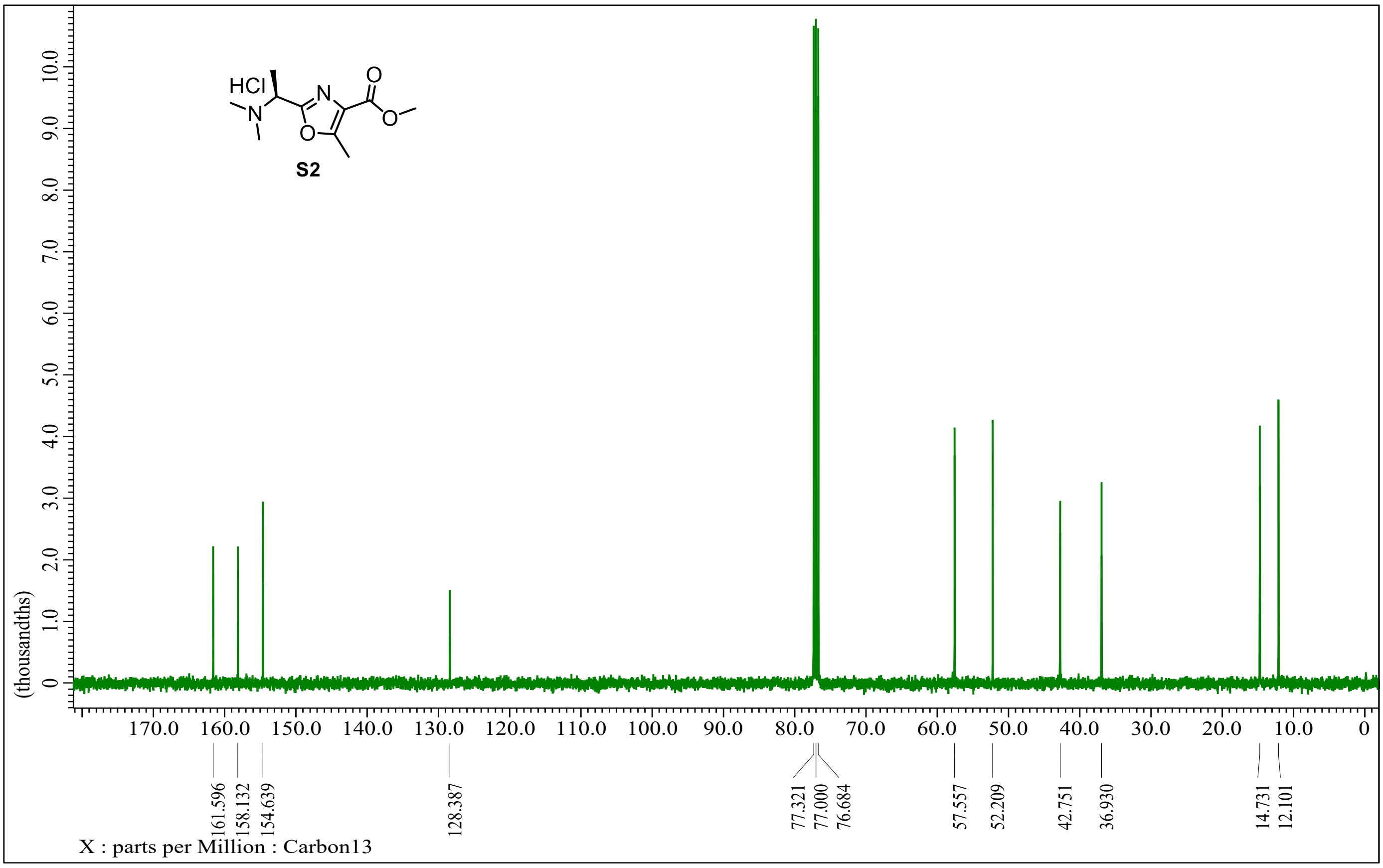

^{1}H NMR spectrum of compound **S4** (400 MHz, $CDCl_3$)

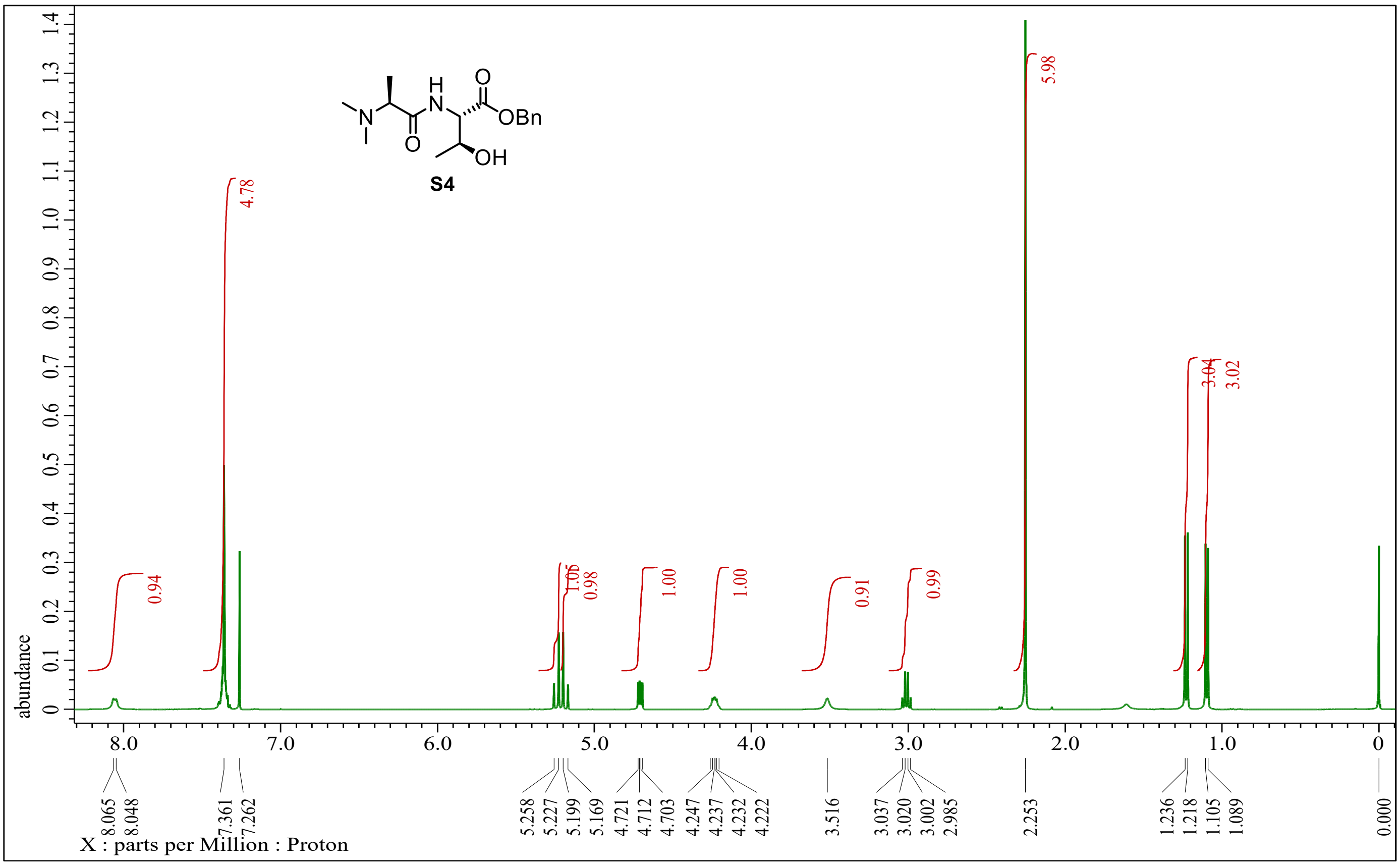

^{13}C NMR spectrum of compound **S4** (100 MHz, $CDCl_3$)

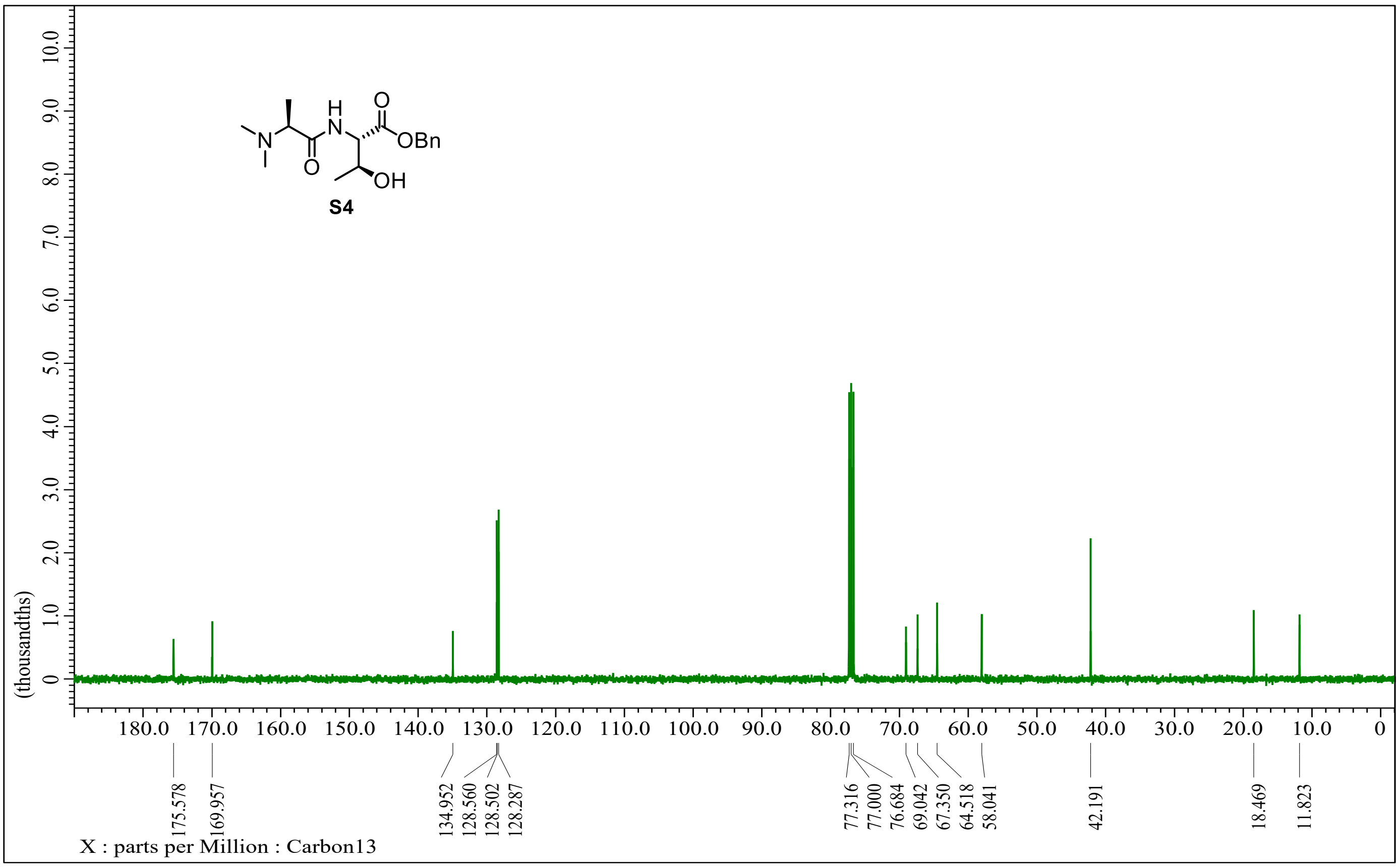

^{1}H NMR spectrum of compound **S5** (400 MHz, $CDCl_3$)

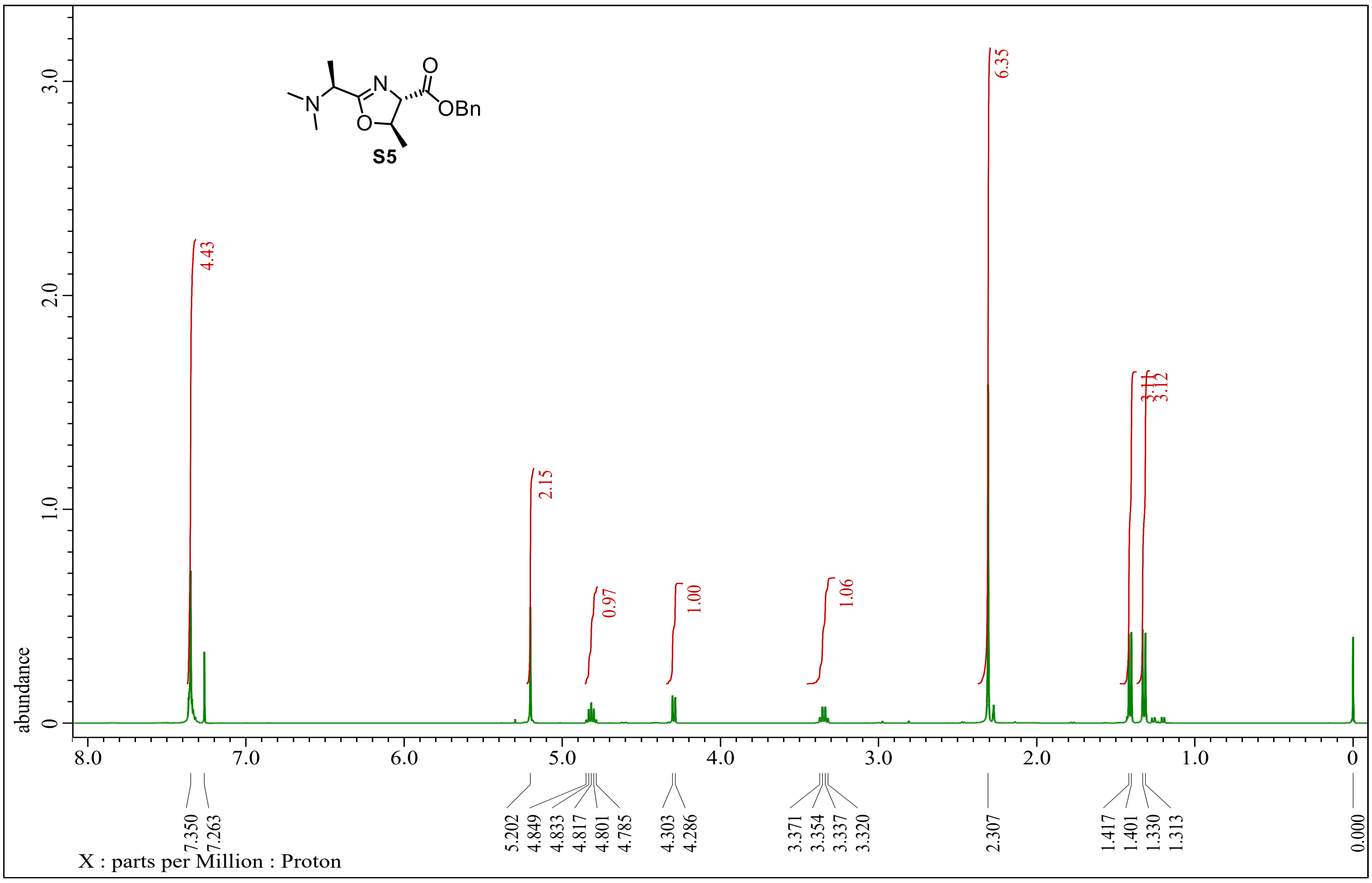

^{13}C NMR spectrum of compound **S5** (100 MHz, $CDCl_3$)

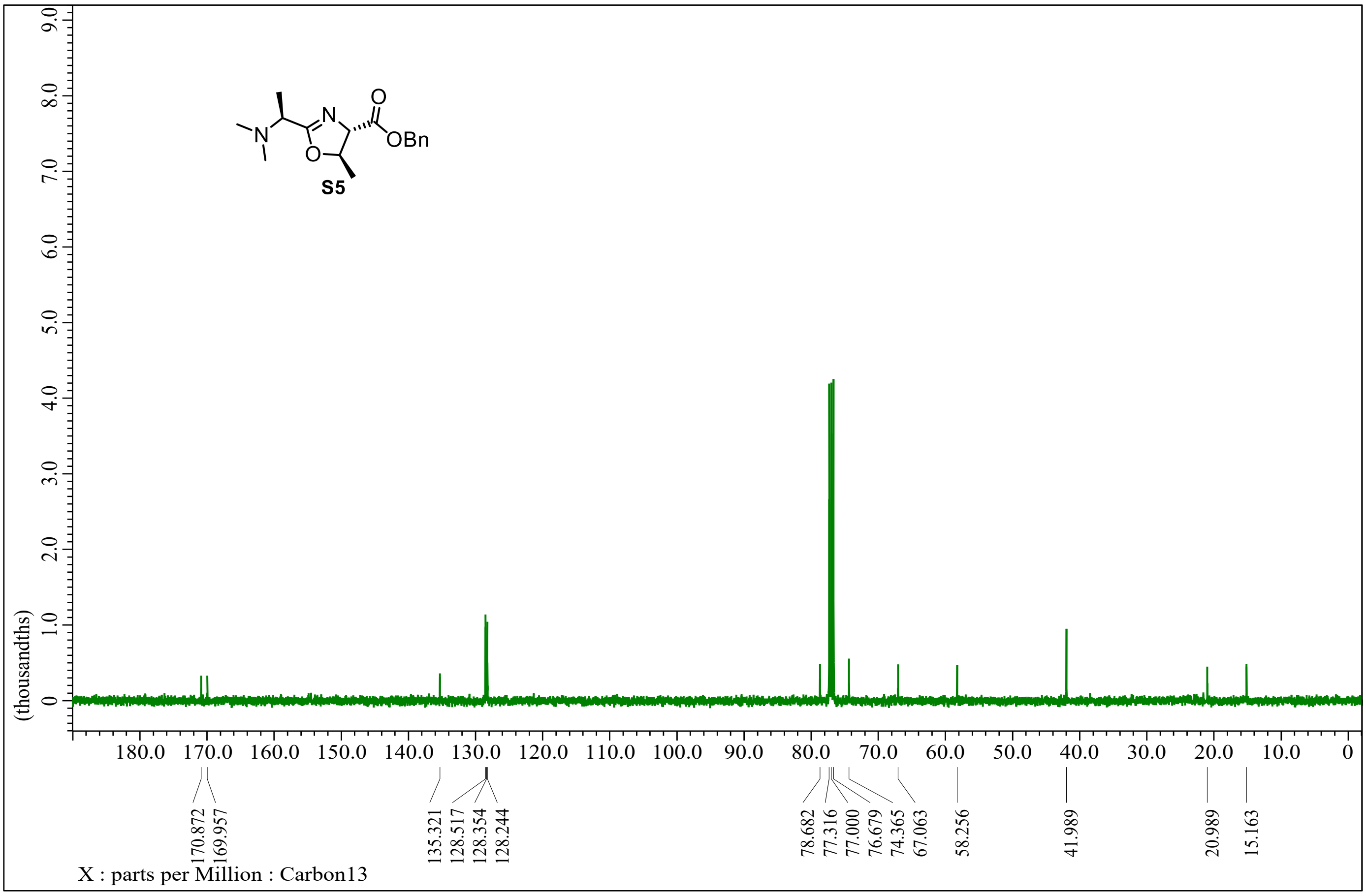

^{1}H NMR spectrum of compound **S7** (400 MHz, $CDCl_3$)

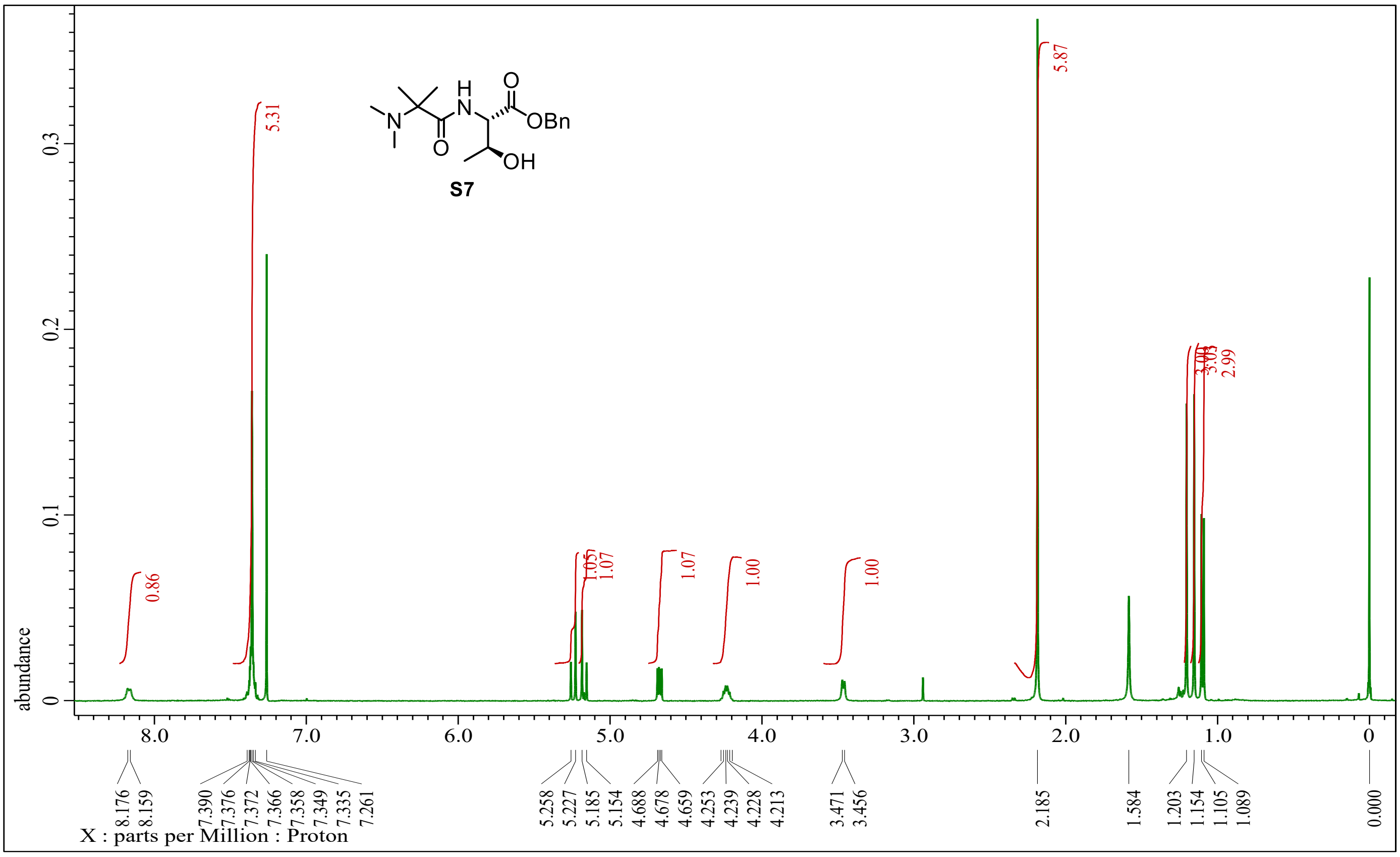

^{13}C NMR spectrum of compound **S7** (100 MHz, $CDCl_3$)

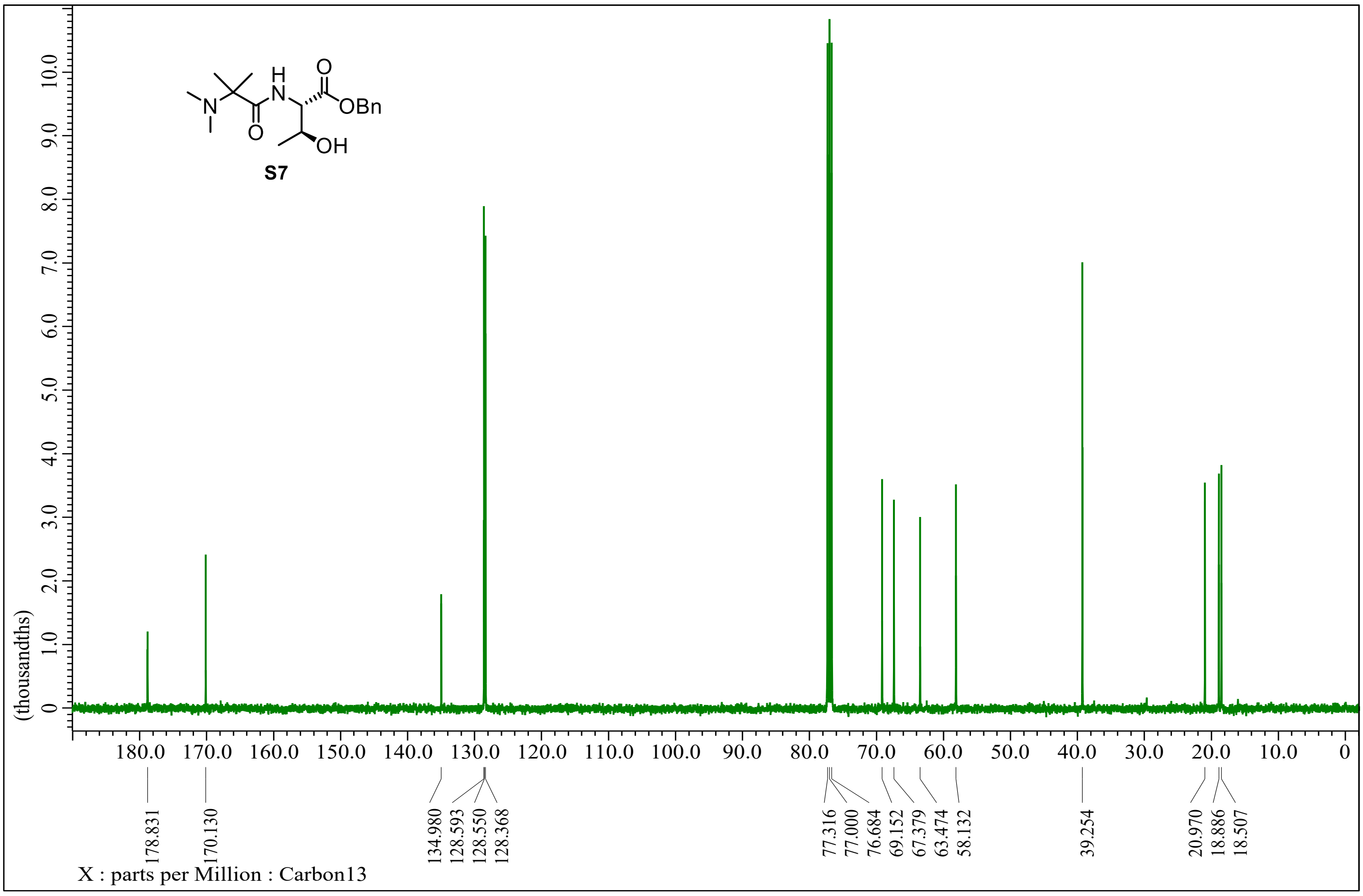

^{1}H NMR spectrum of compound **S8** (400 MHz, $CDCl_3$)

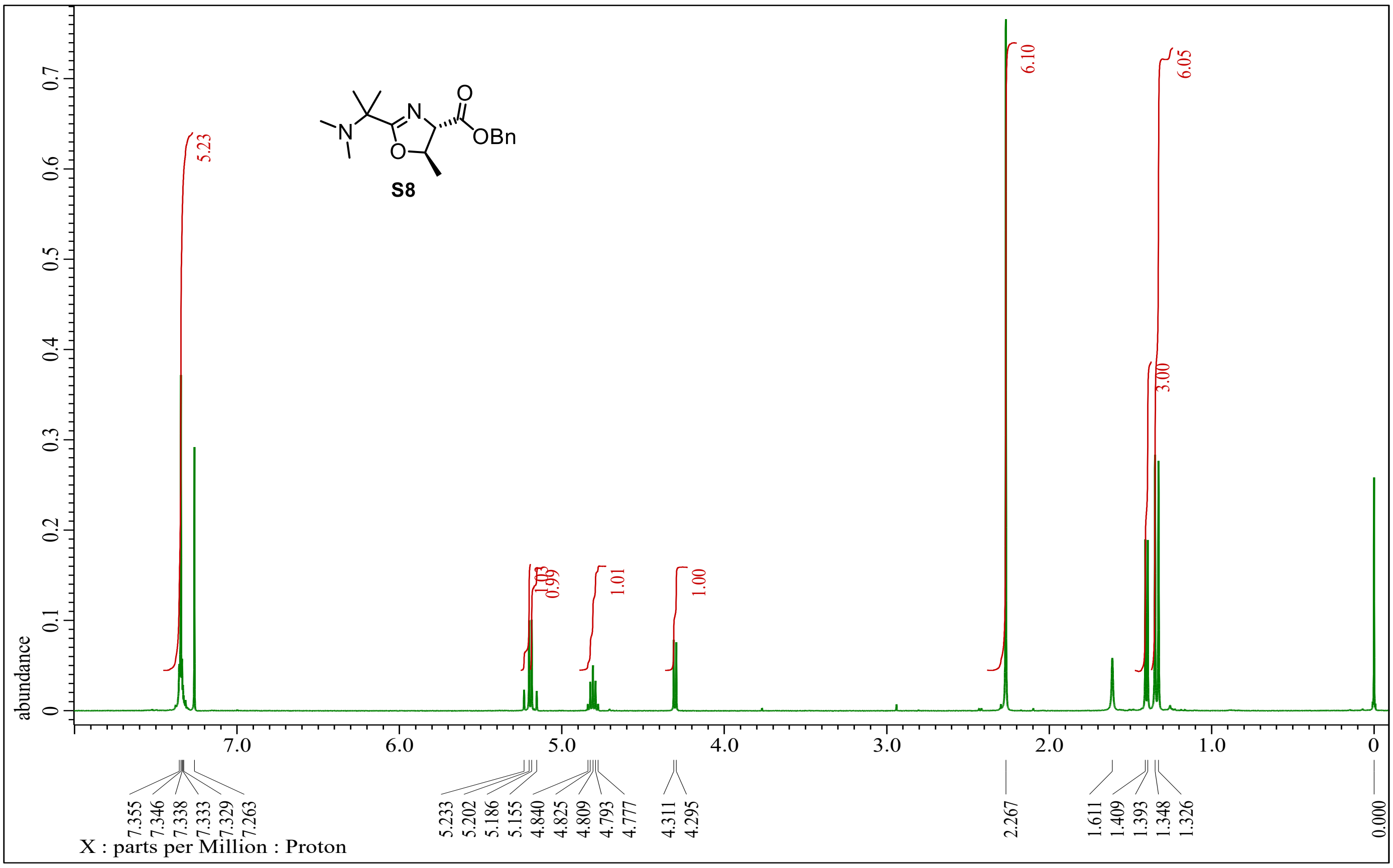

^{13}C NMR spectrum of compound **S8** (100 MHz, $CDCl_3$)

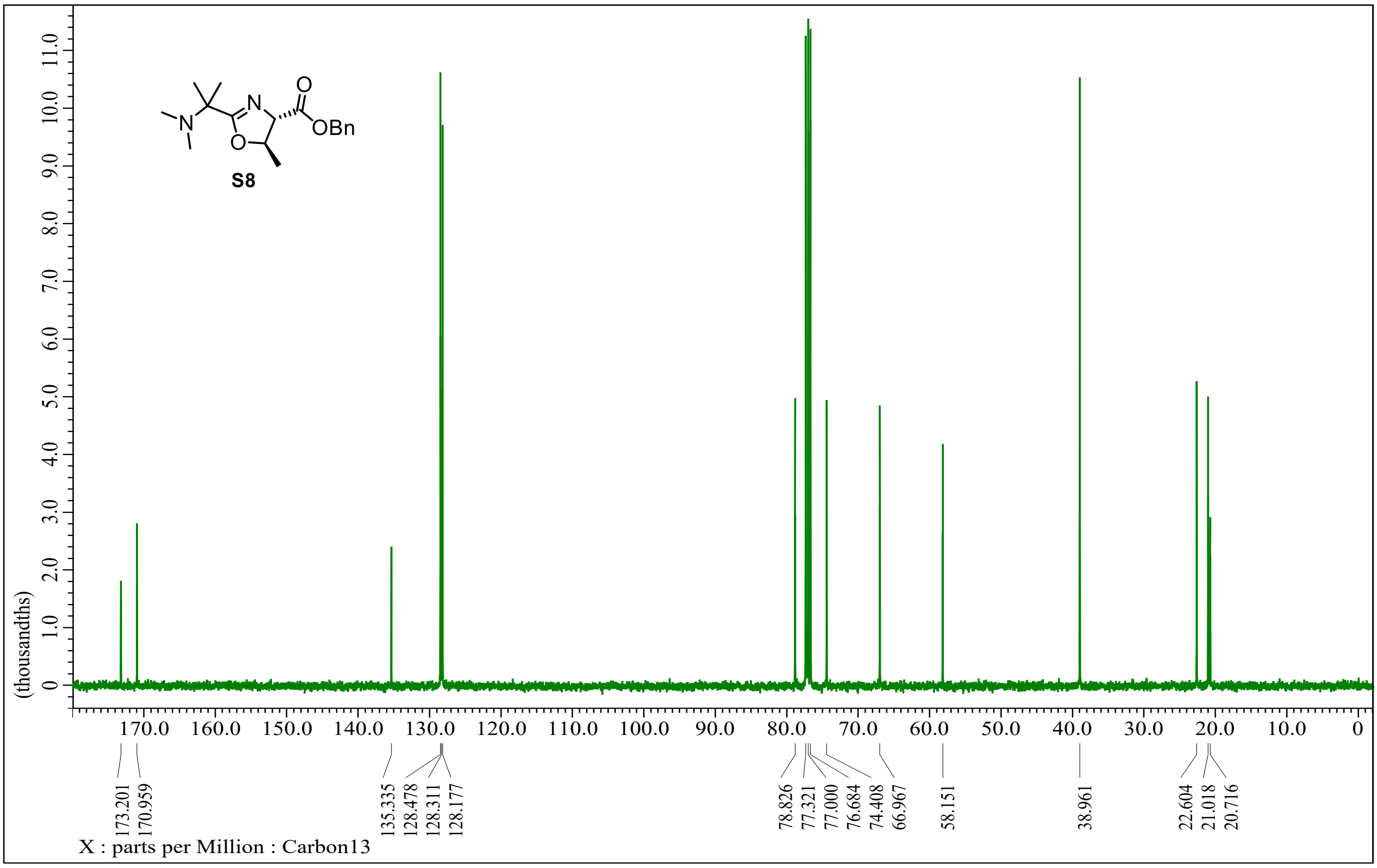

^{1}H NMR spectrum of compound **10** (600 MHz, $CDCl_3$)

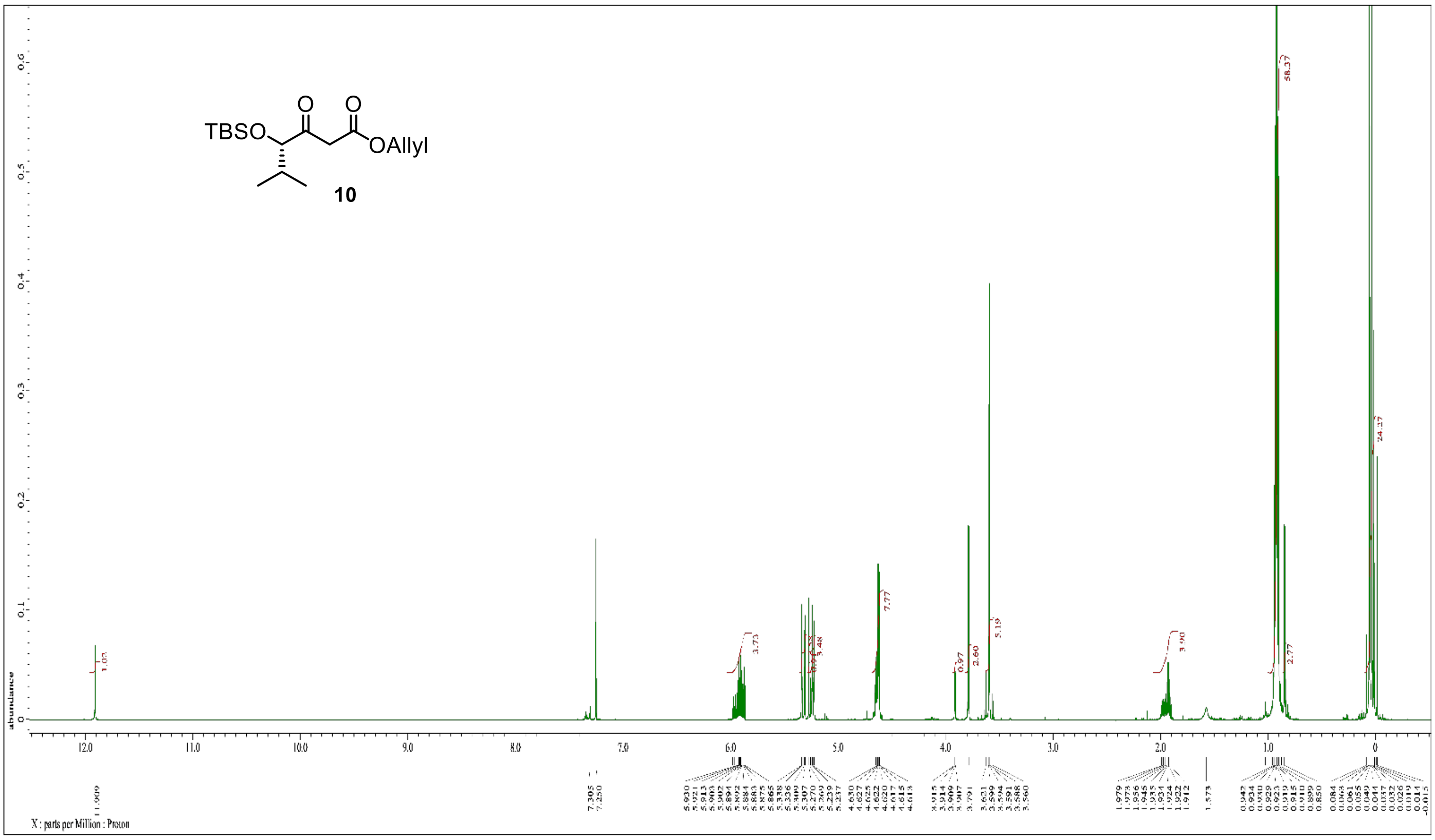

^{13}C NMR spectrum of compound **10** (125 MHz, $CDCl_3$)

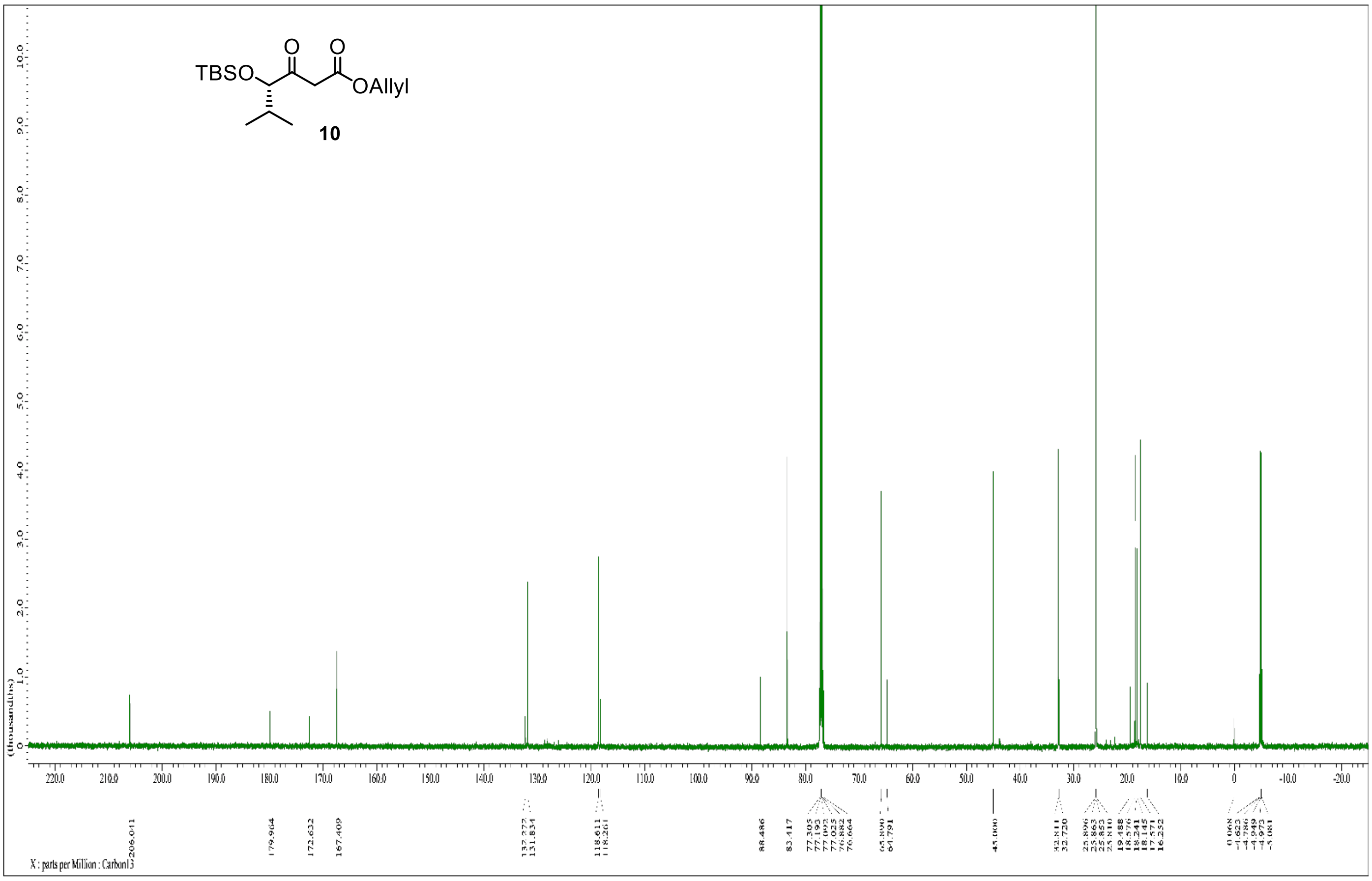

^{1}H NMR spectrum of compound **11** (400 MHz, $CDCl_3$)

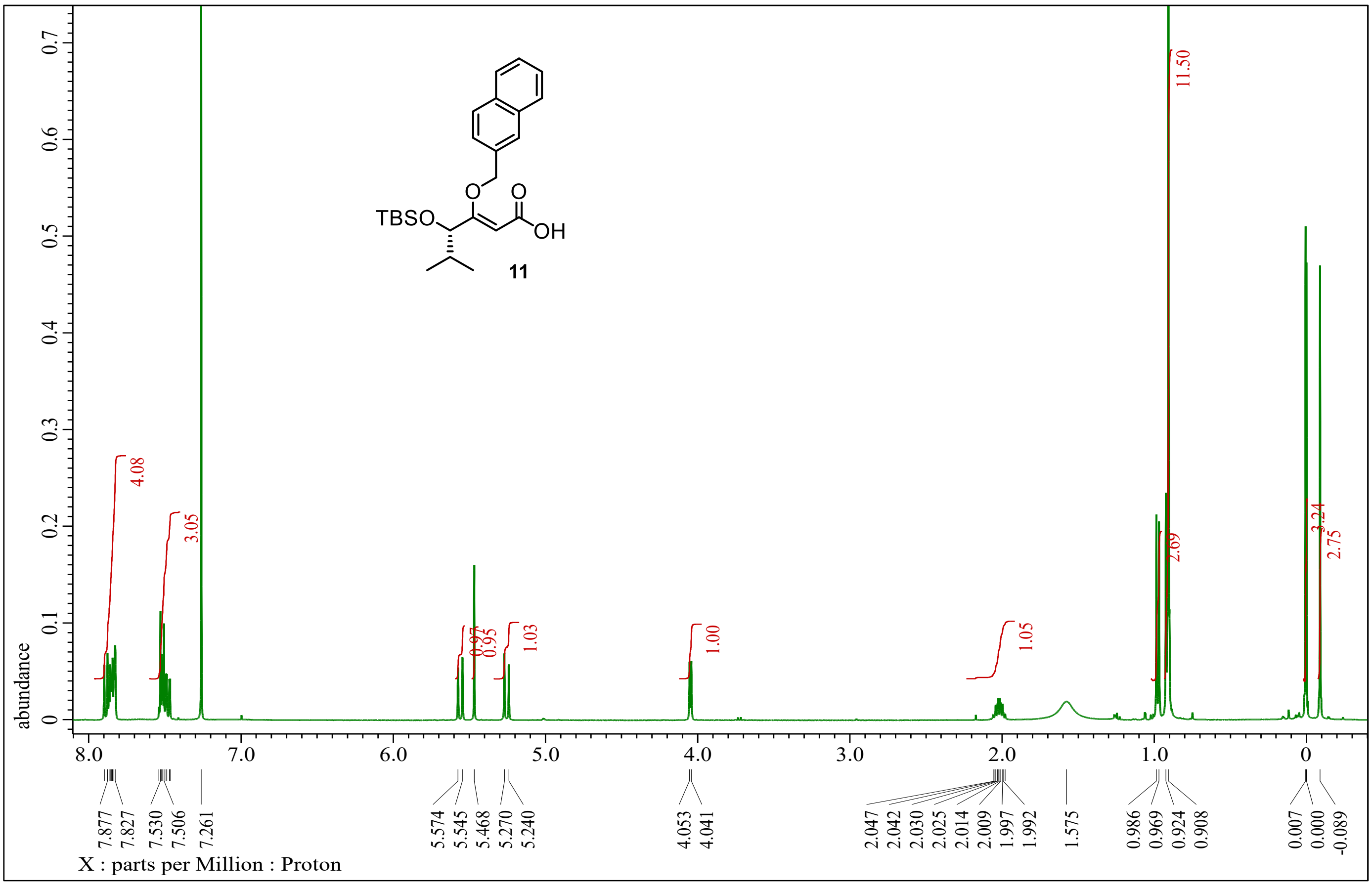

^{13}C NMR spectrum of compound **11** (100 MHz, $CDCl_3$)

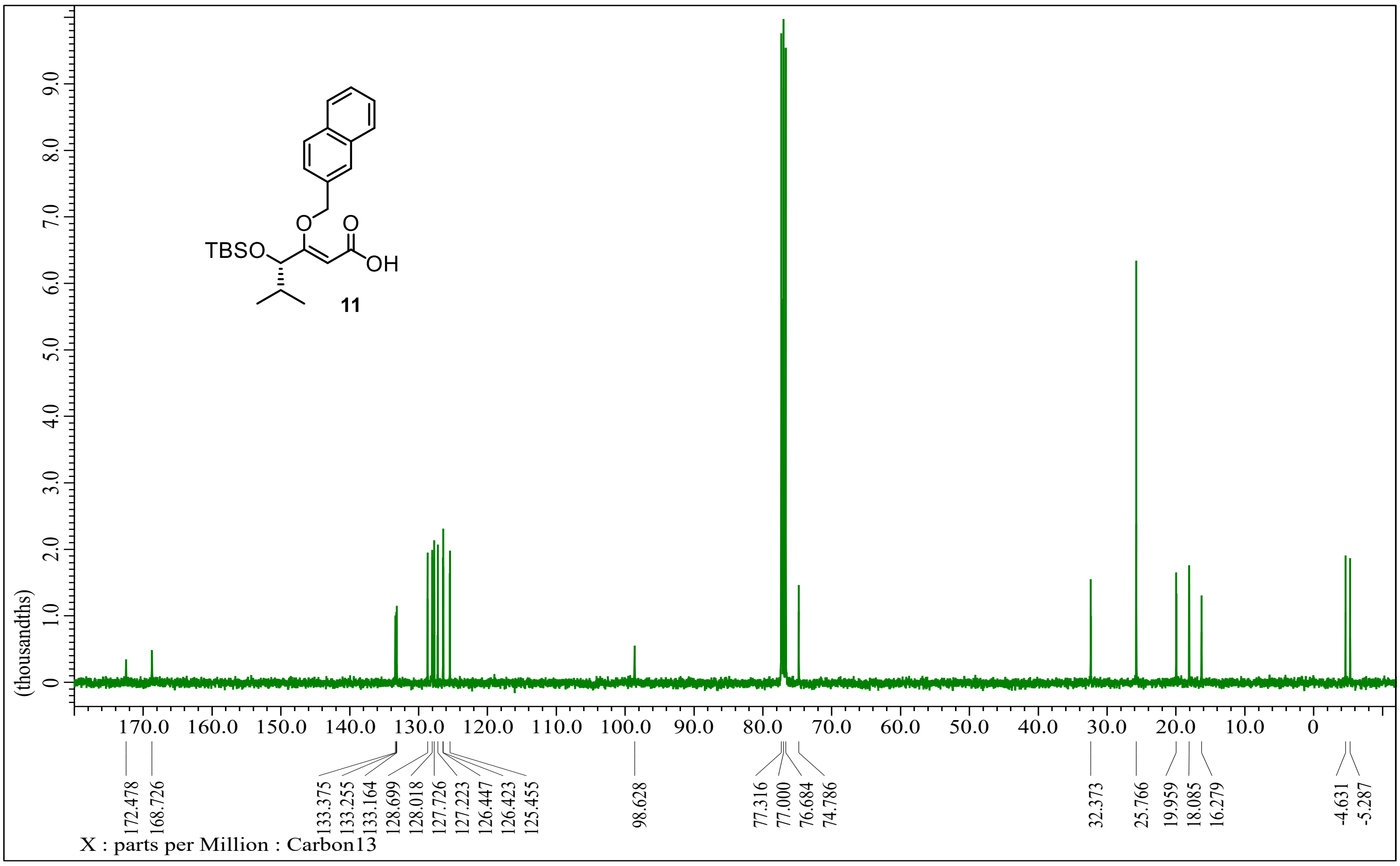

^1H NMR spectrum of compound **9a** (400 MHz, $CDCl_3$)

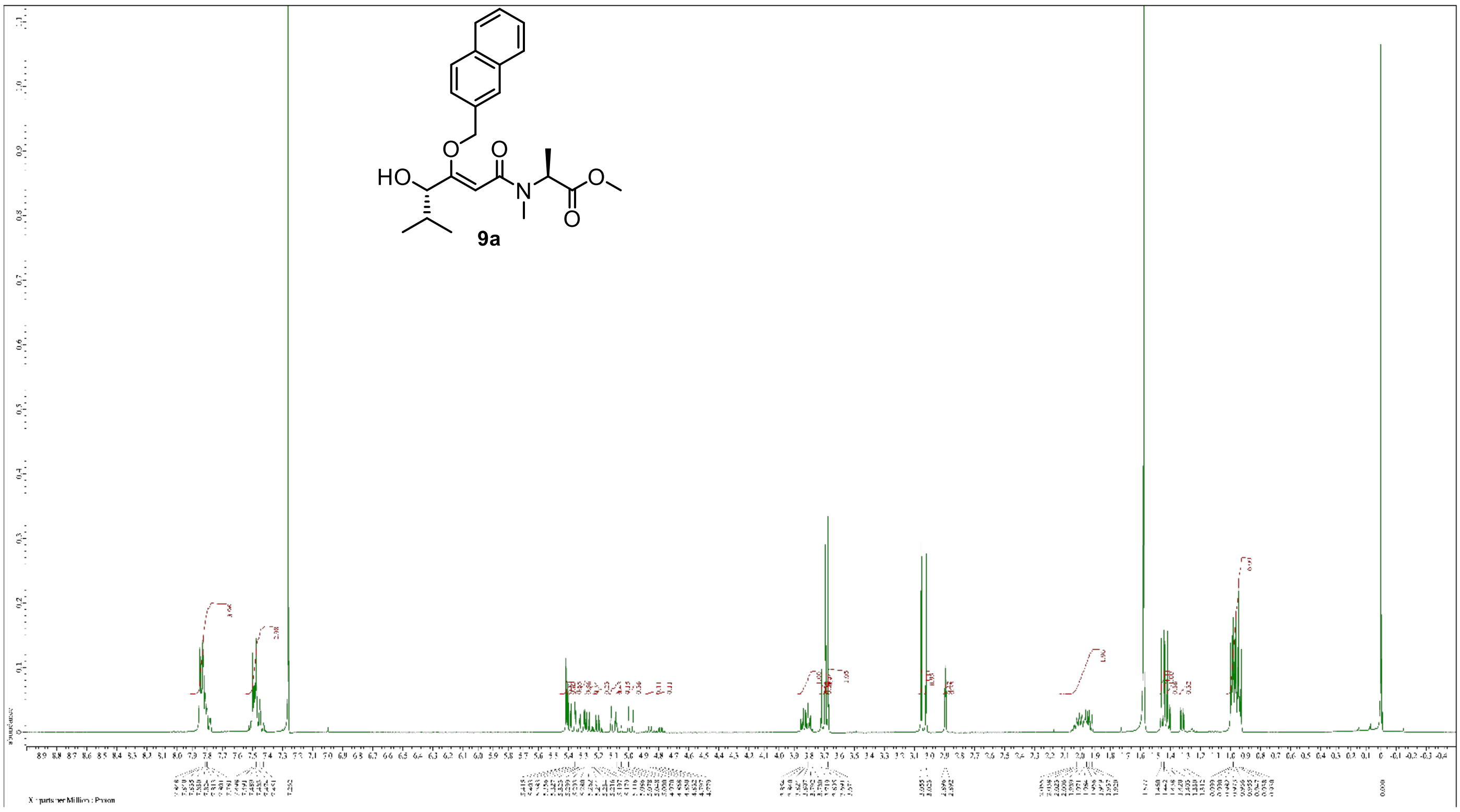

^{13}C NMR spectrum of compound **9a** (100 MHz, $CDCl_3$)

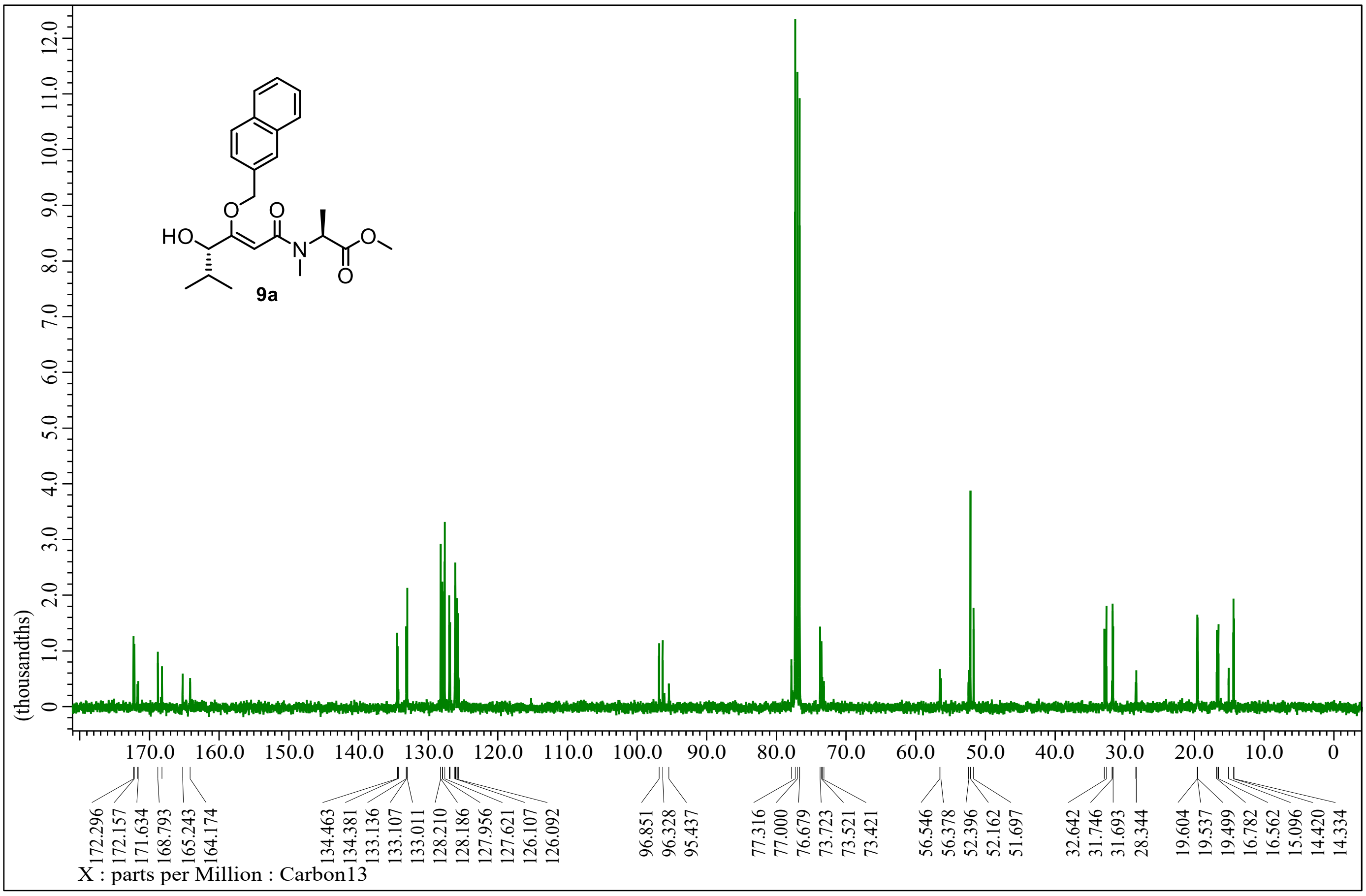

^{1}H NMR spectrum of compound **9b** (400 MHz, $CDCl_3$)

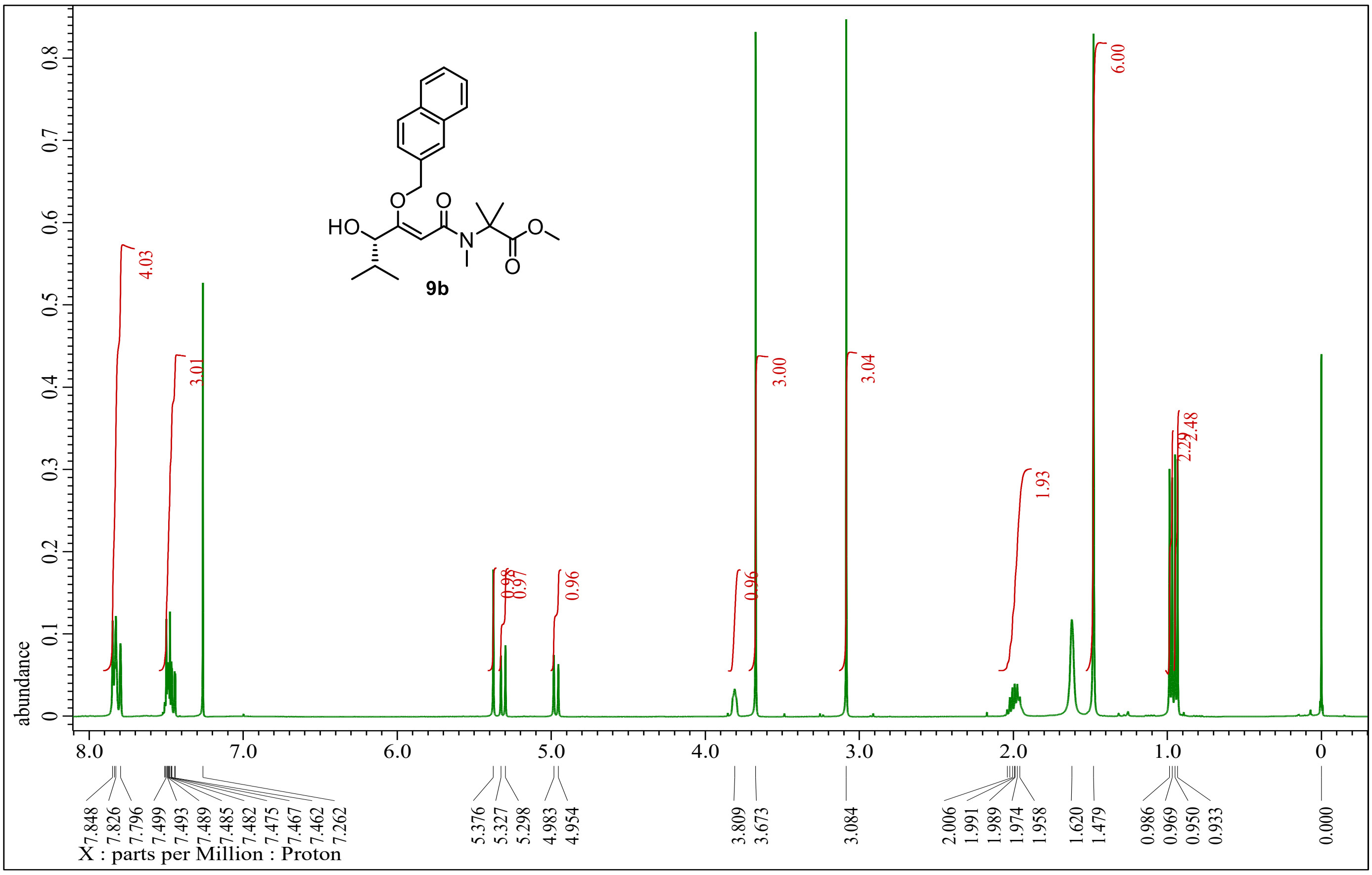

^{13}C NMR spectrum of compound **9b** (100 MHz, $CDCl_3$)

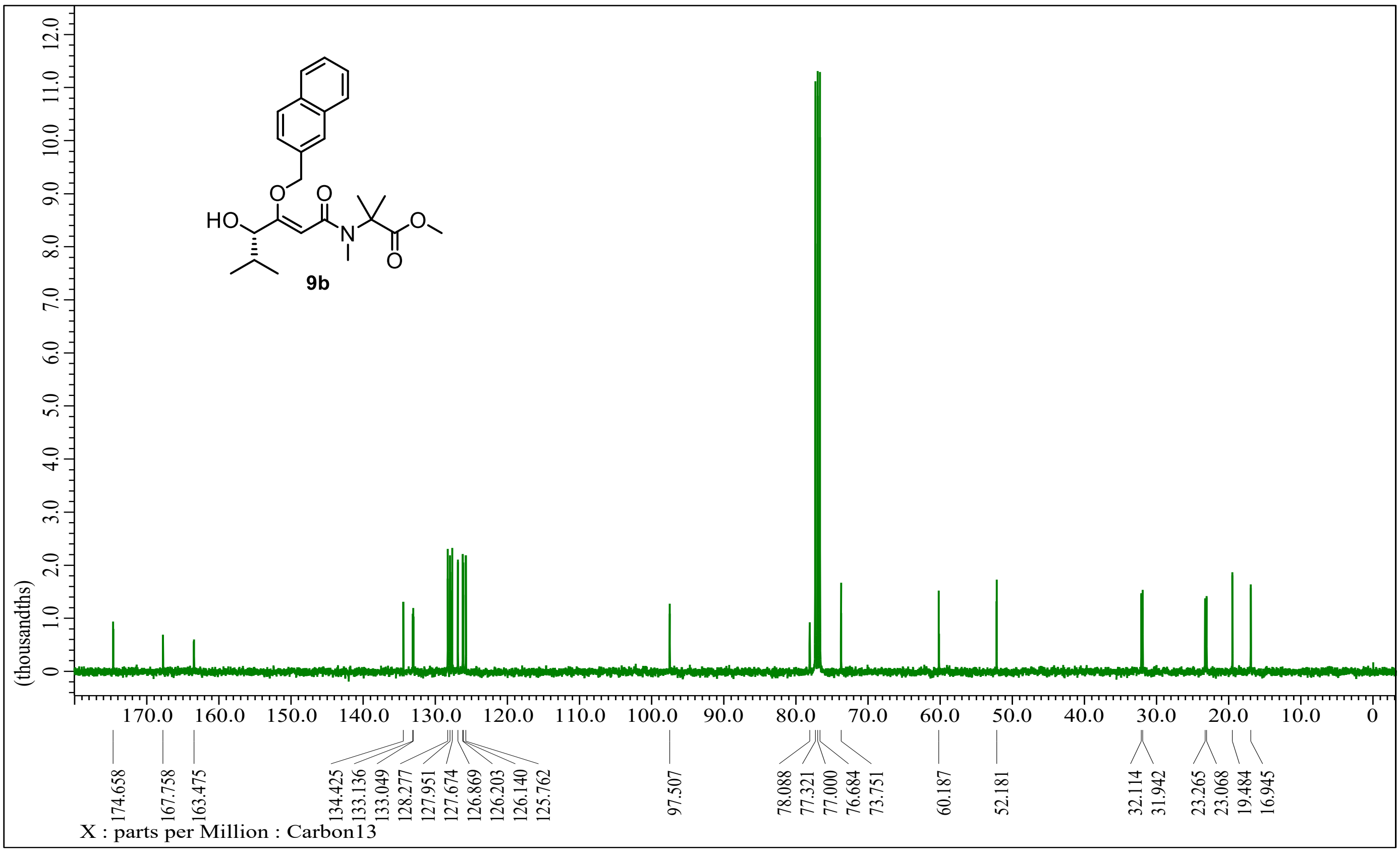

^{1}H NMR spectrum of compound **8** (600 MHz, DMSO-d_6, 60 ℃)

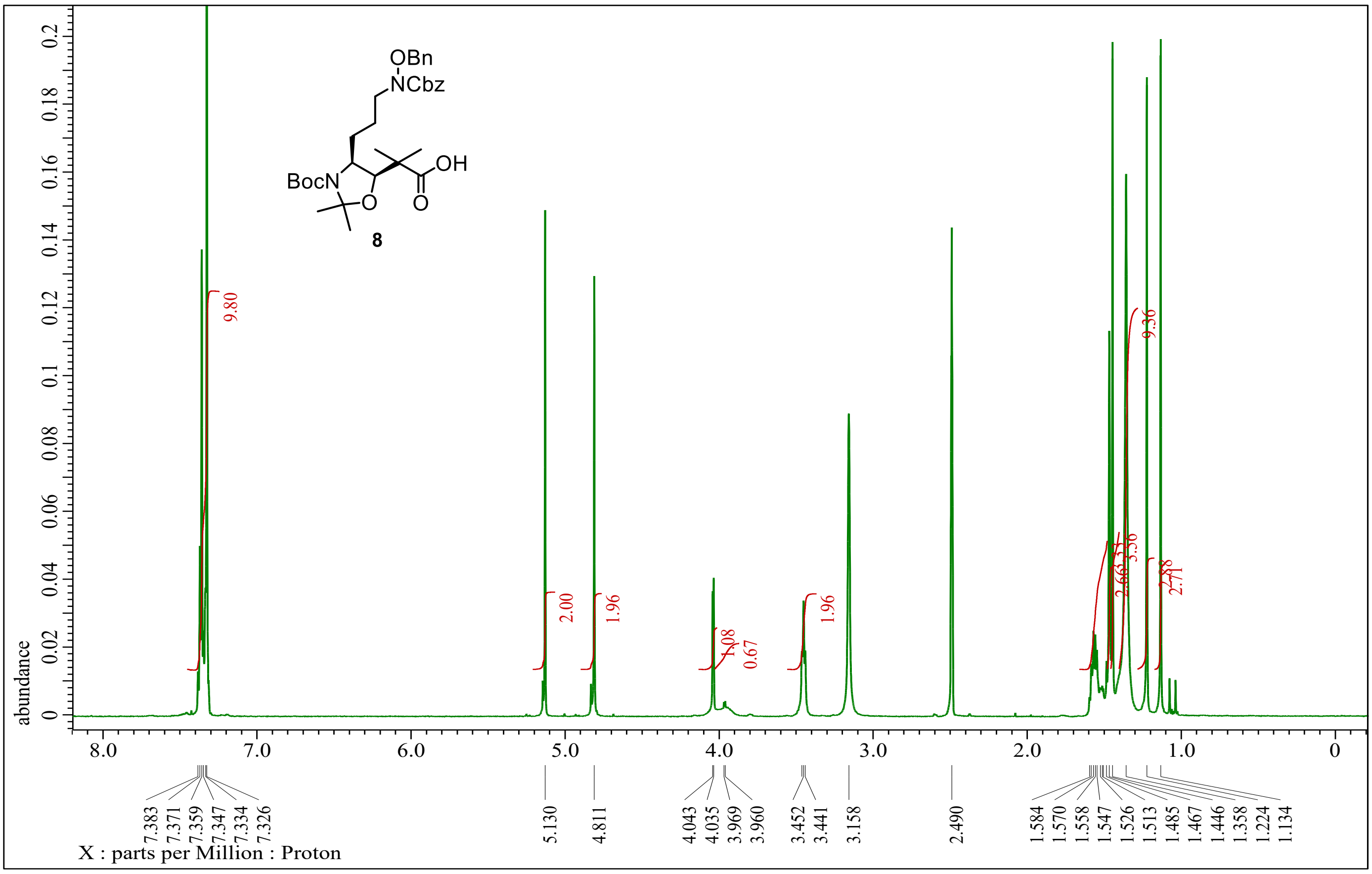

^{13}C NMR spectrum of compound **8** (125 MHz, DMSO-d_6, 60 ℃)

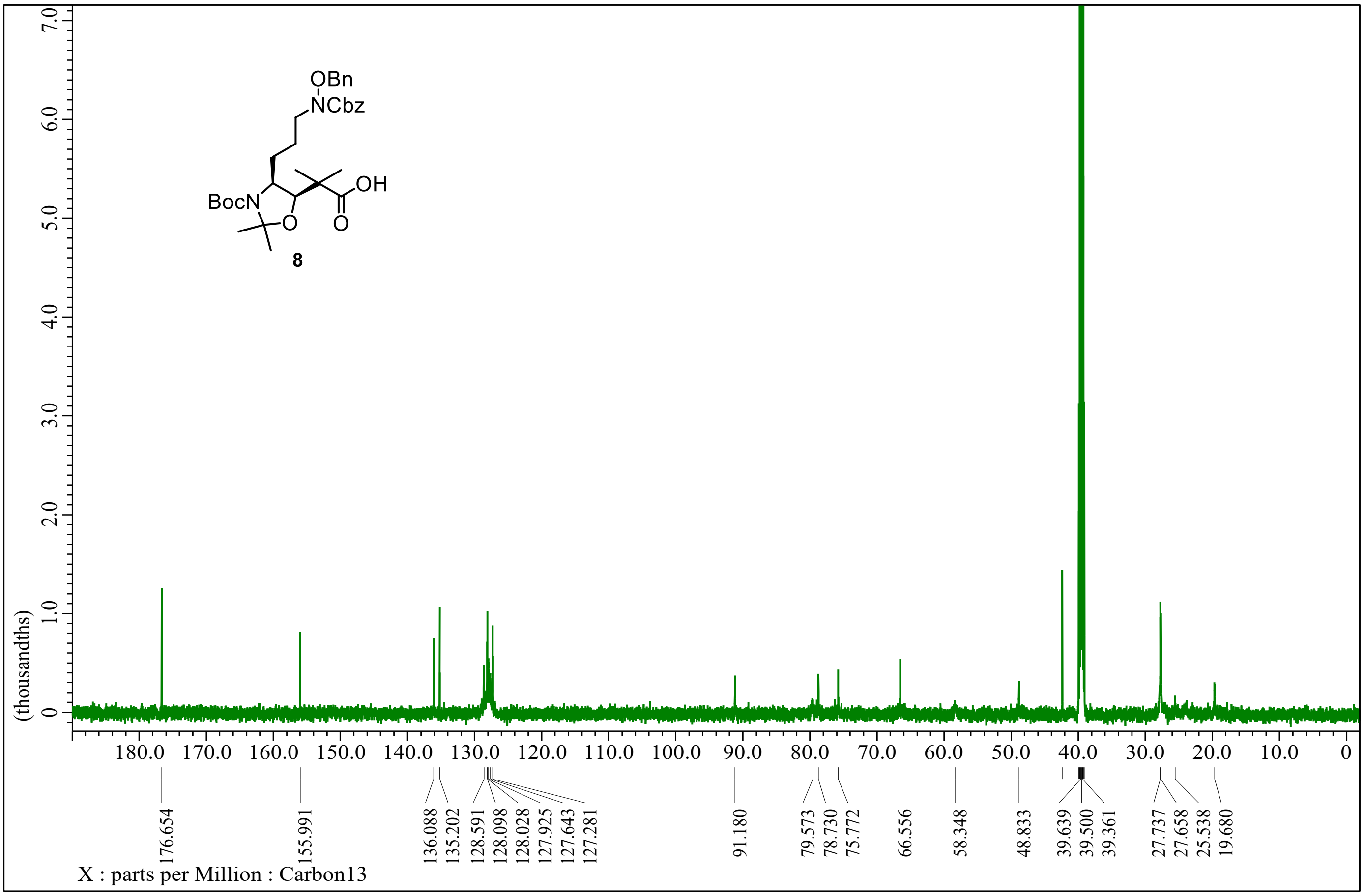

^{1}H NMR spectrum of compound **13a** (600 MHz, $CDCl_3$)

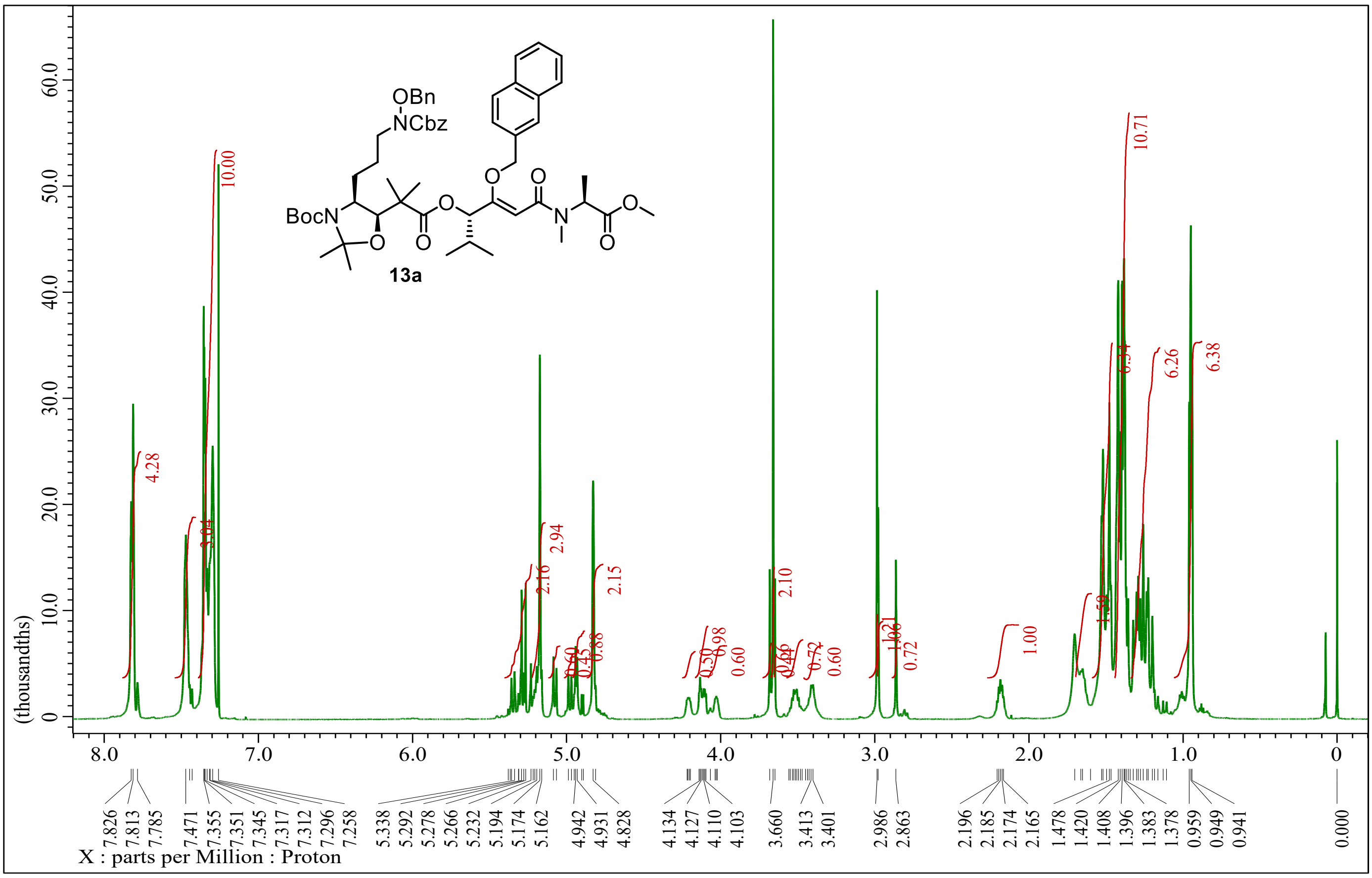

^{13}C NMR spectrum of compound **13a** (125 MHz, $CDCl_3$)

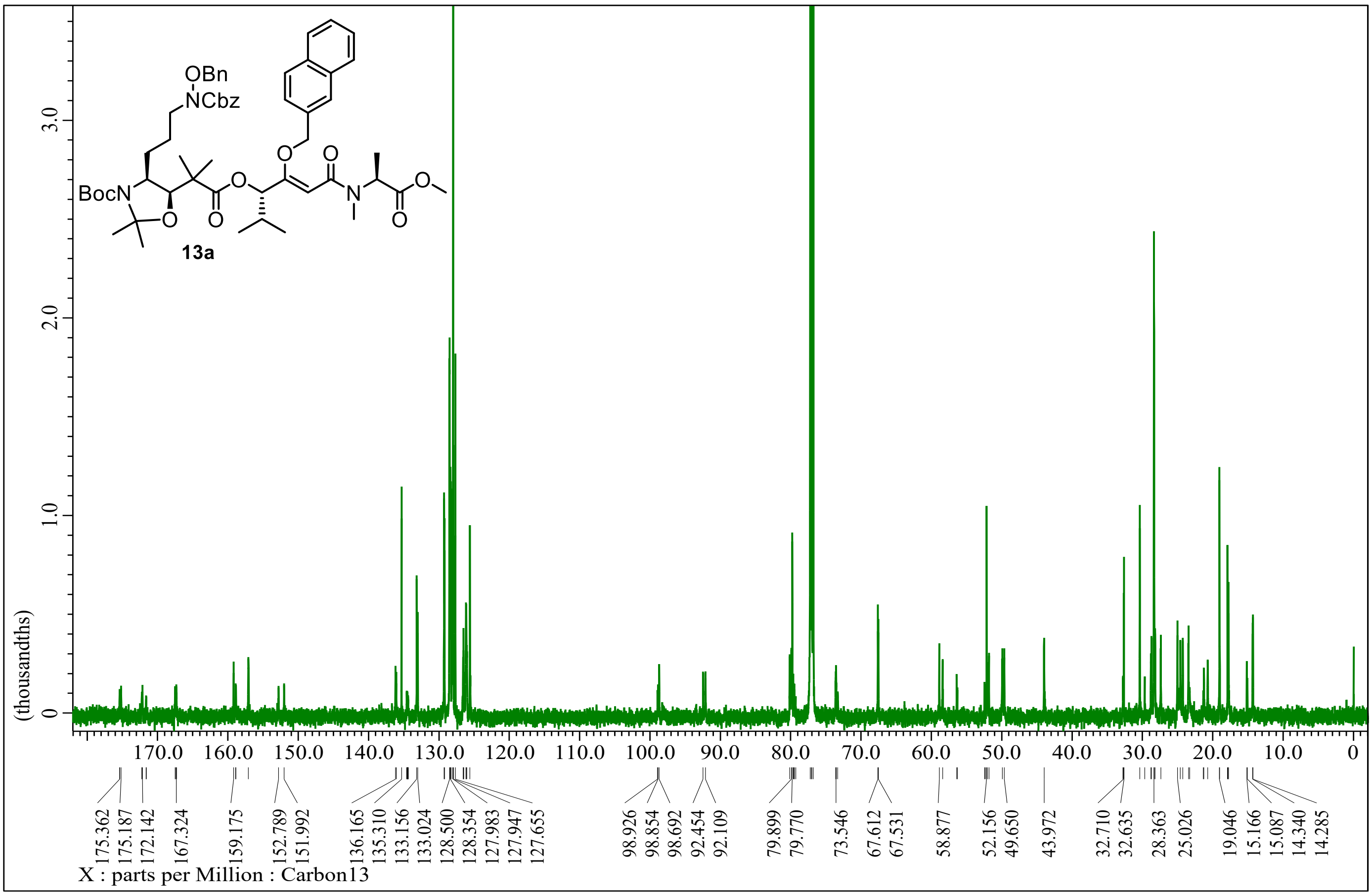

^{1}H NMR spectrum of compound **6a** (600 MHz, $CDCl_3$)

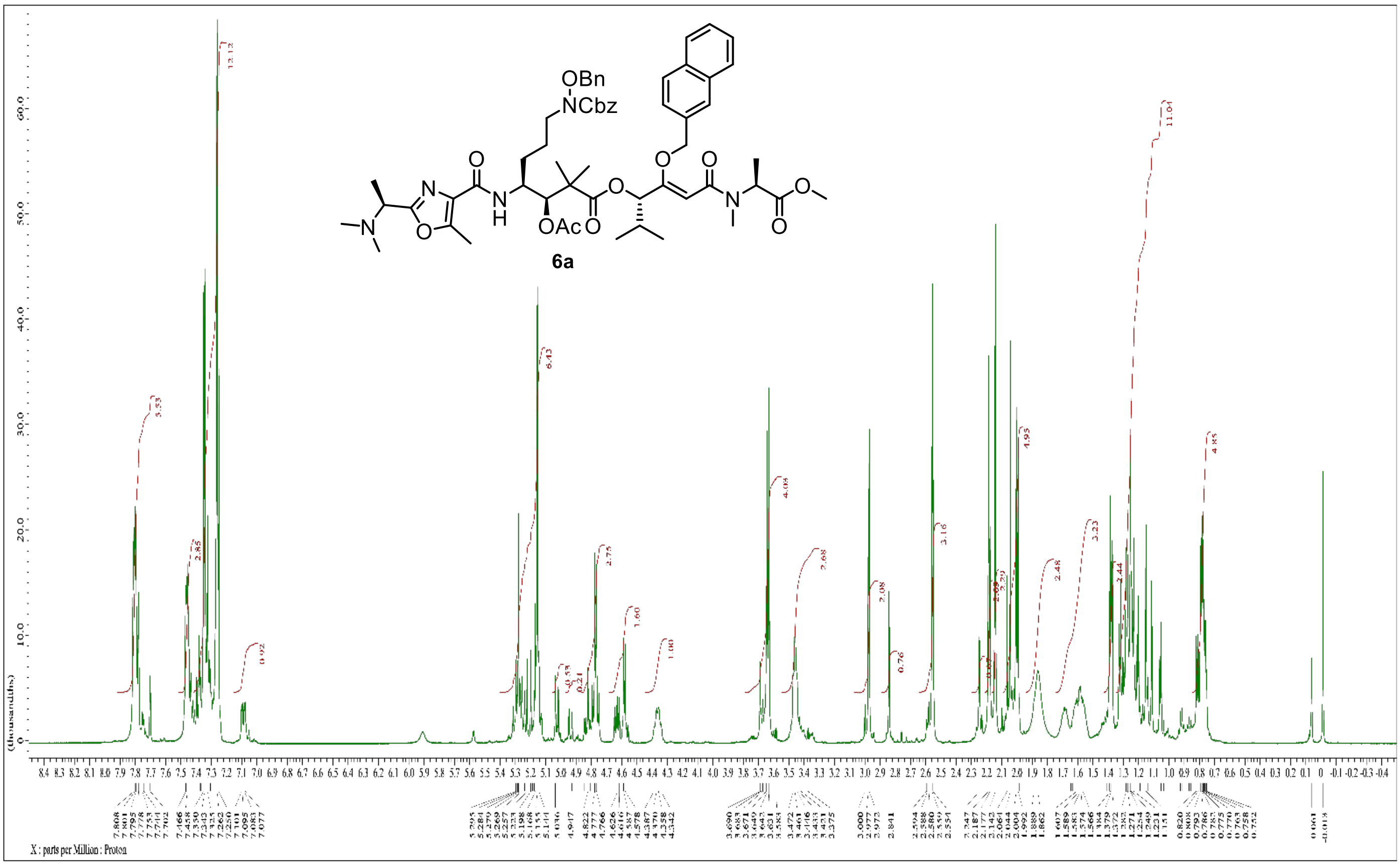

^{13}C NMR spectrum of compound **6a** (125 MHz, $CDCl_3$)

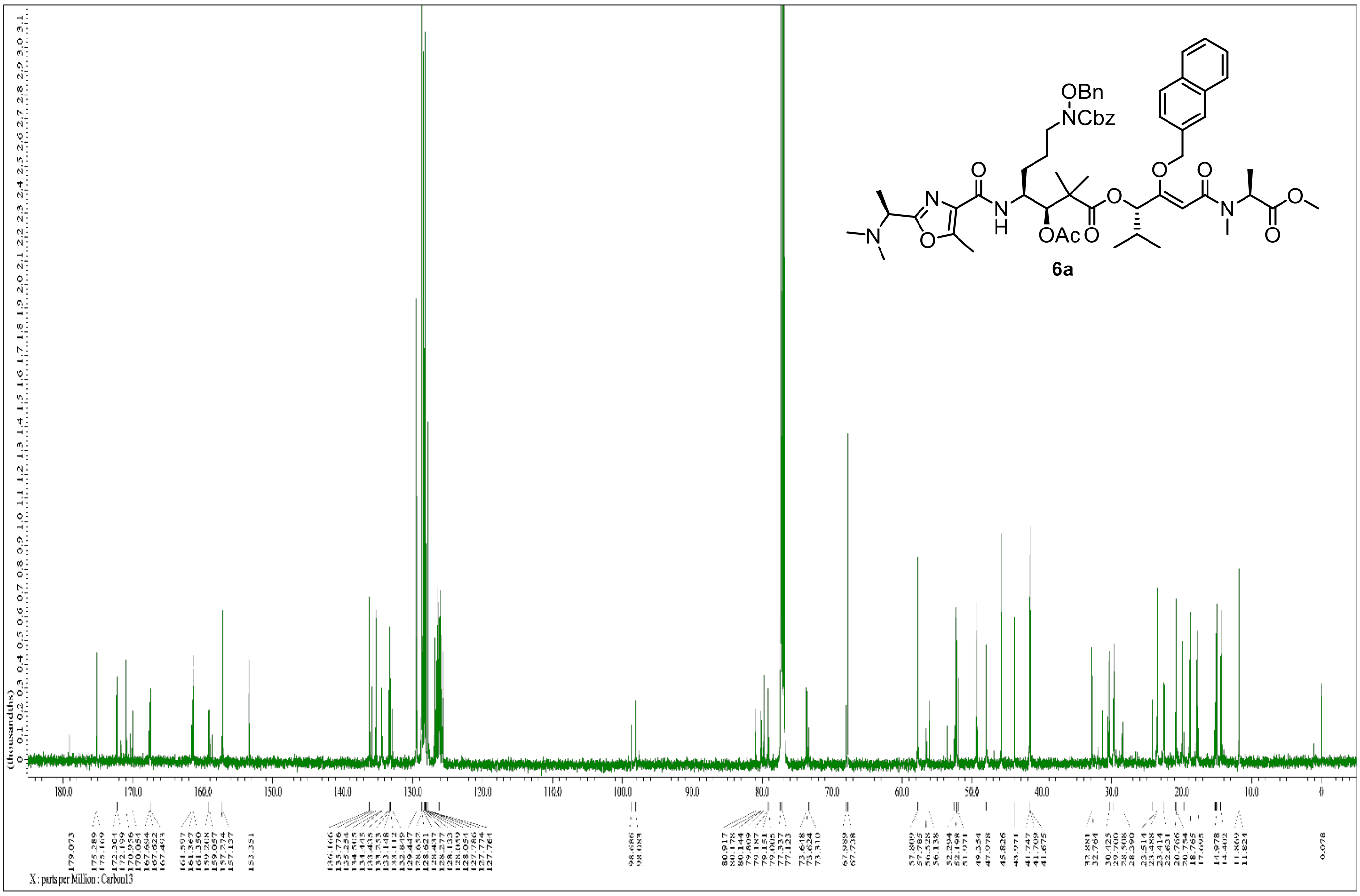

^{1}H NMR spectrum of compound **5a** (600 MHz, CD_3OD)

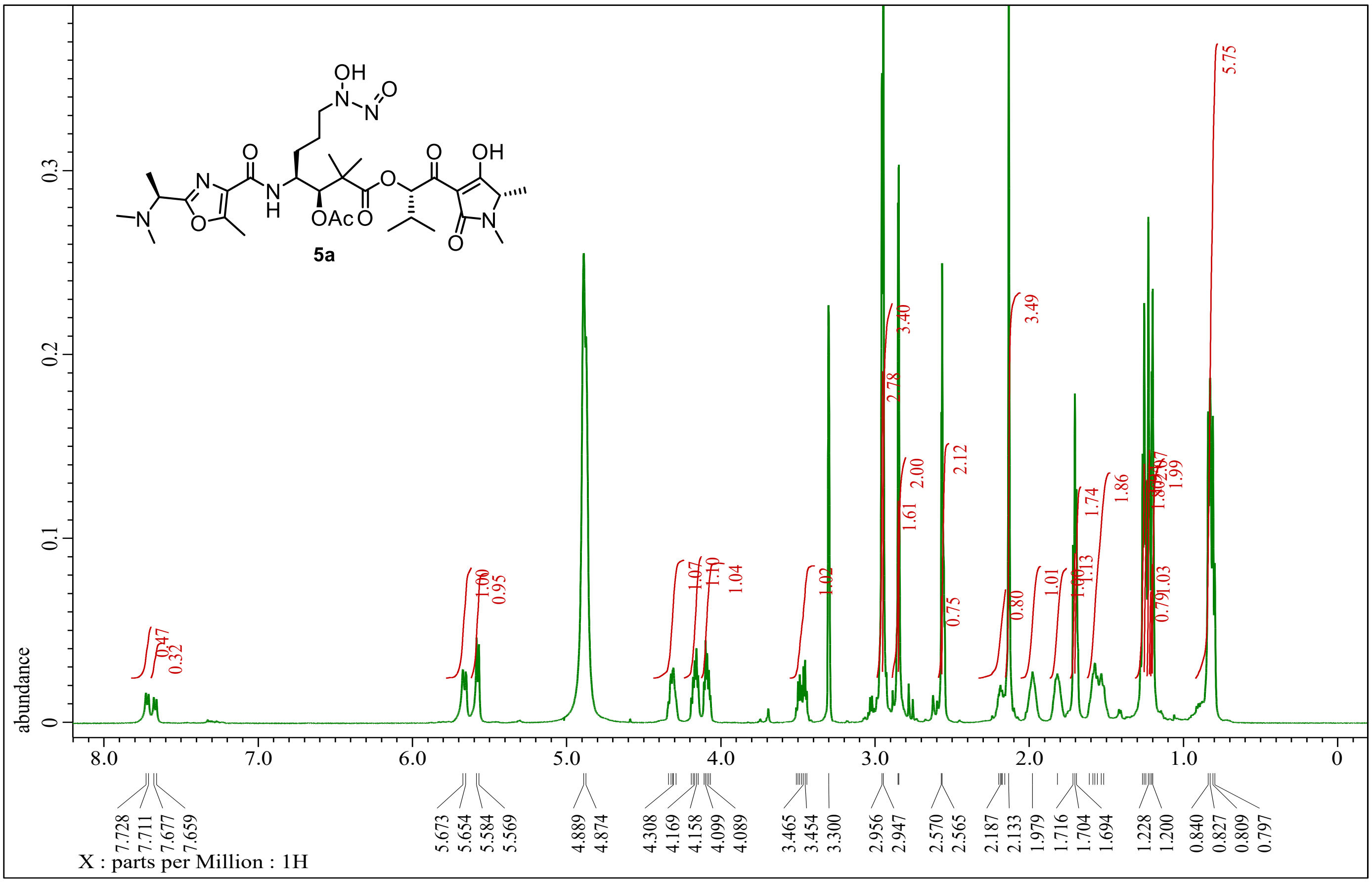

^{13}C NMR spectrum of compound **5a** (125 MHz, CD_3OD)

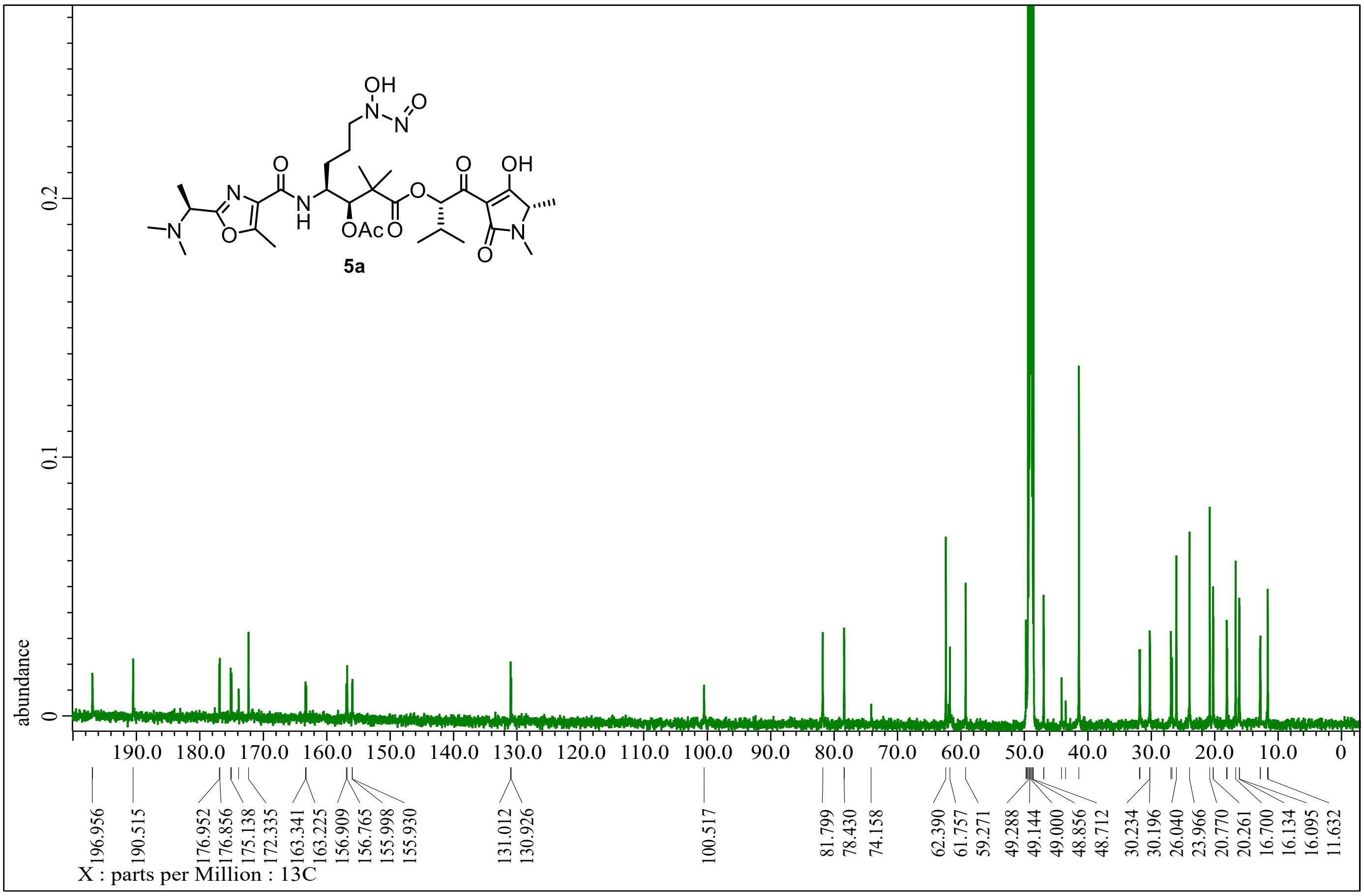

^{1}H NMR spectrum of compound **13b** (600 MHz, $CDCl_3$)

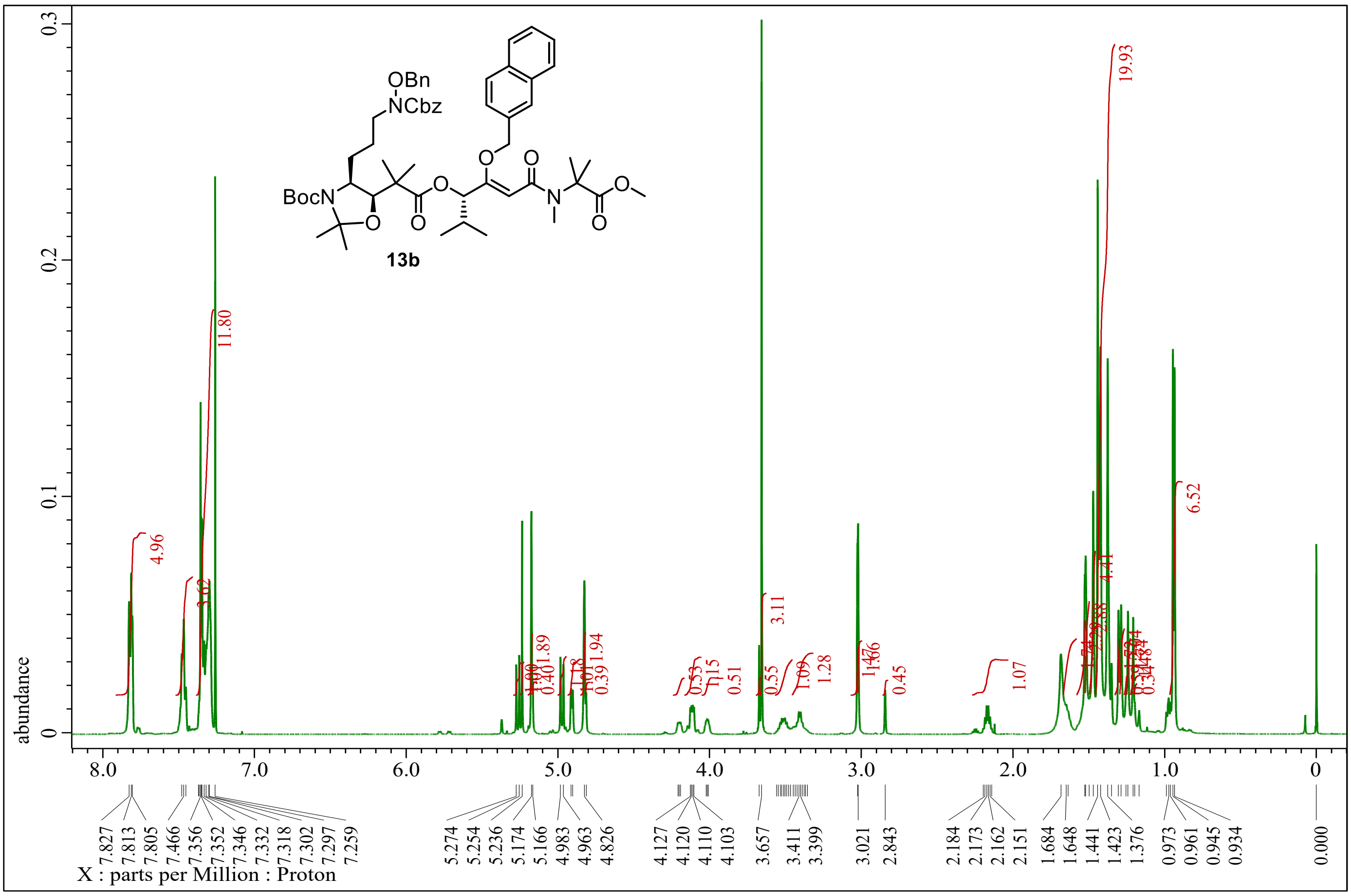

^{13}C NMR spectrum of compound **13b** (125 MHz, $CDCl_3$)

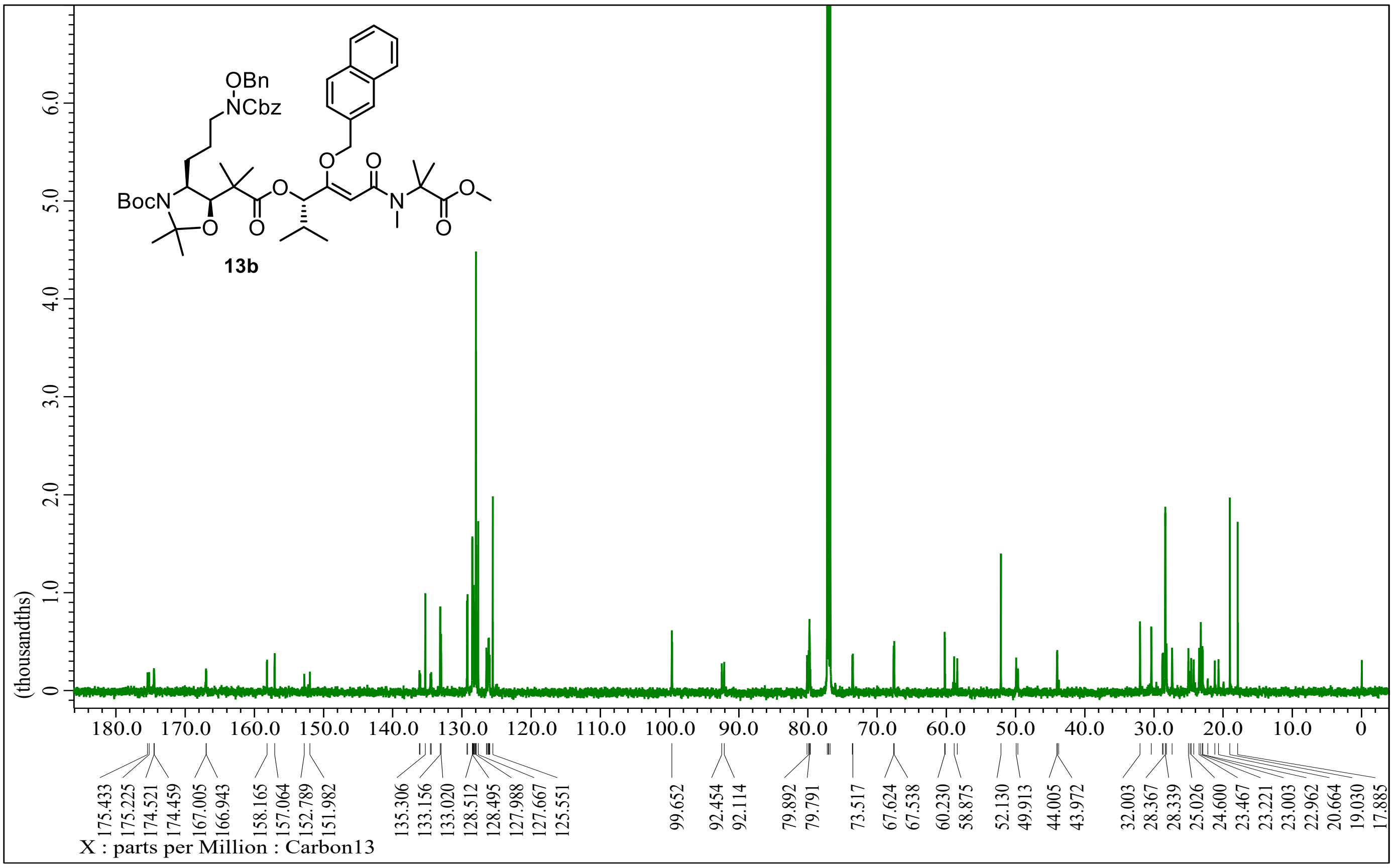

^{1}H NMR spectrum of compound **6b** (600 MHz, $CDCl_3$)

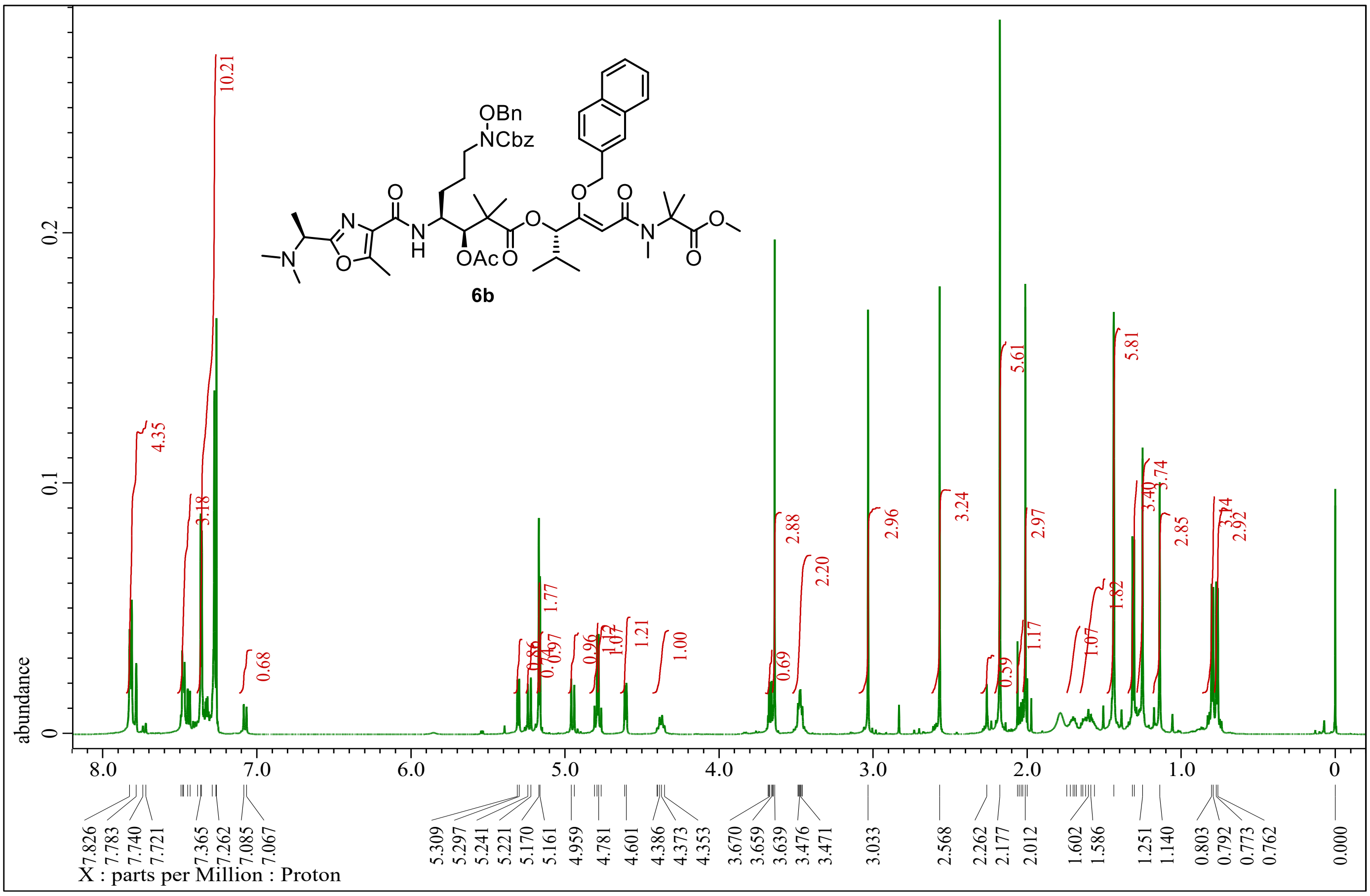

^{13}C NMR spectrum of compound **6b** (125 MHz, $CDCl_3$)

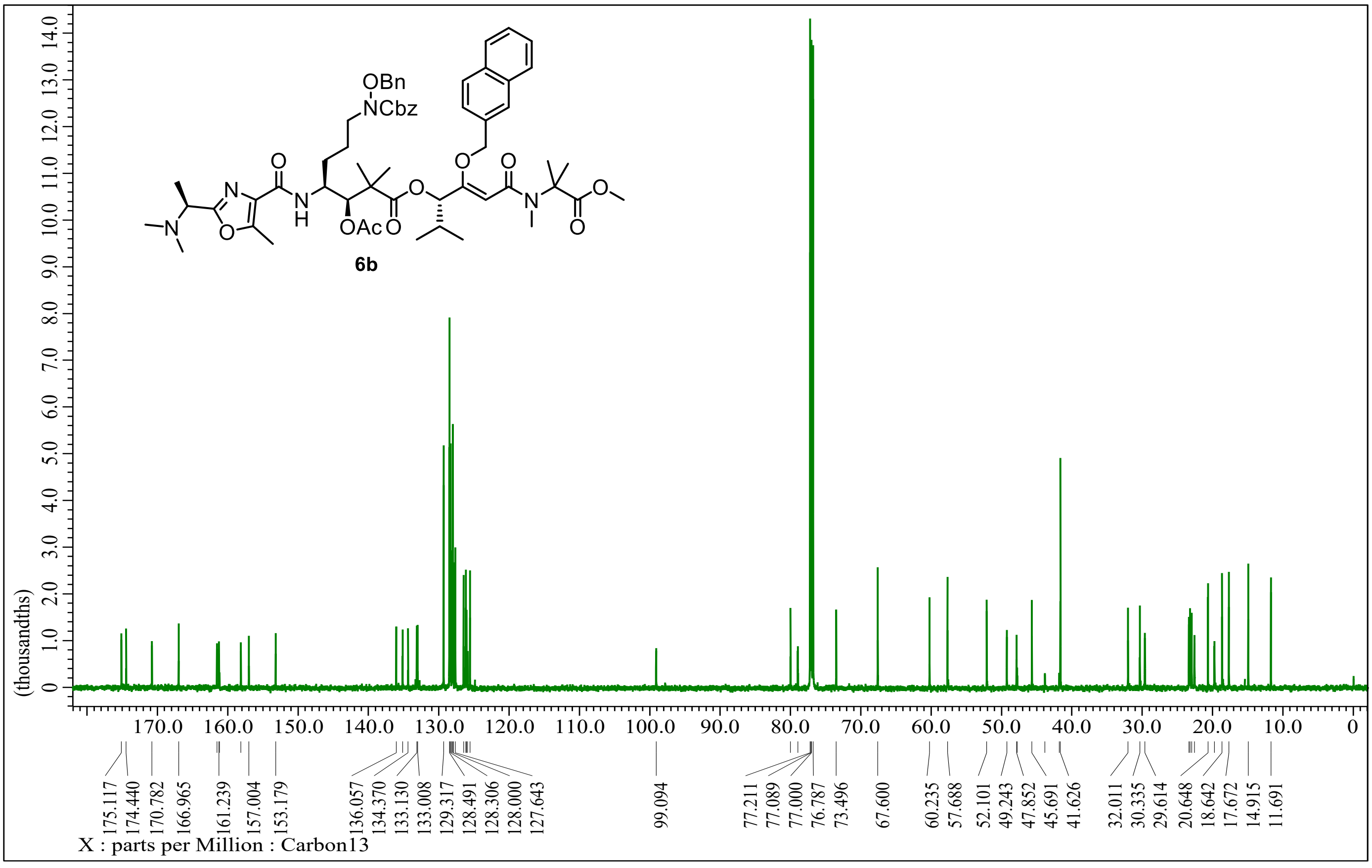

^{1}H NMR spectrum of compound **5b** (600 MHz, CD_3OD)

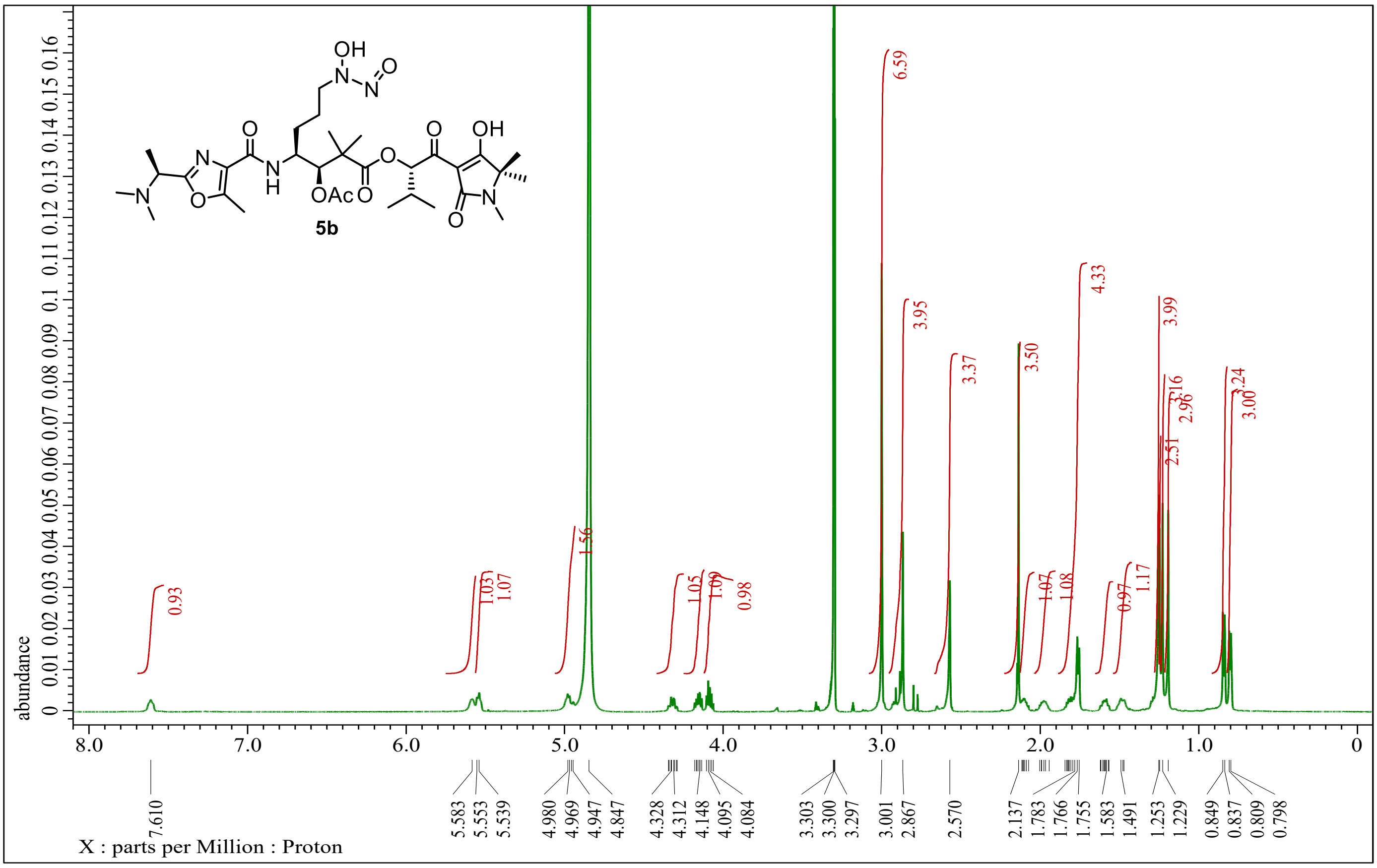

^{13}C NMR spectrum of compound **5b** (125 MHz, CD_3OD)

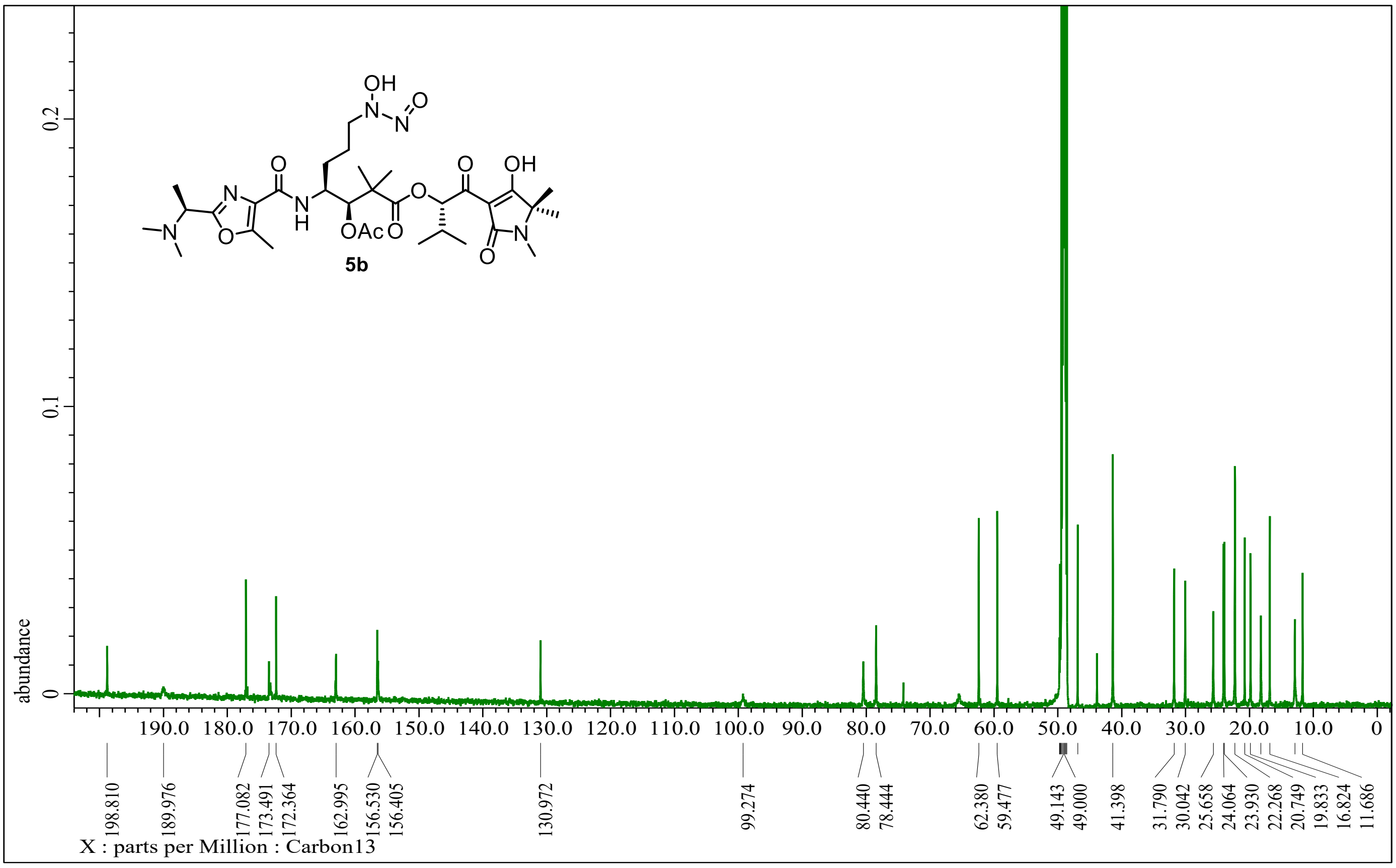

^{1}H NMR spectrum of compound **15a** (600 MHz, $CDCl_3$)

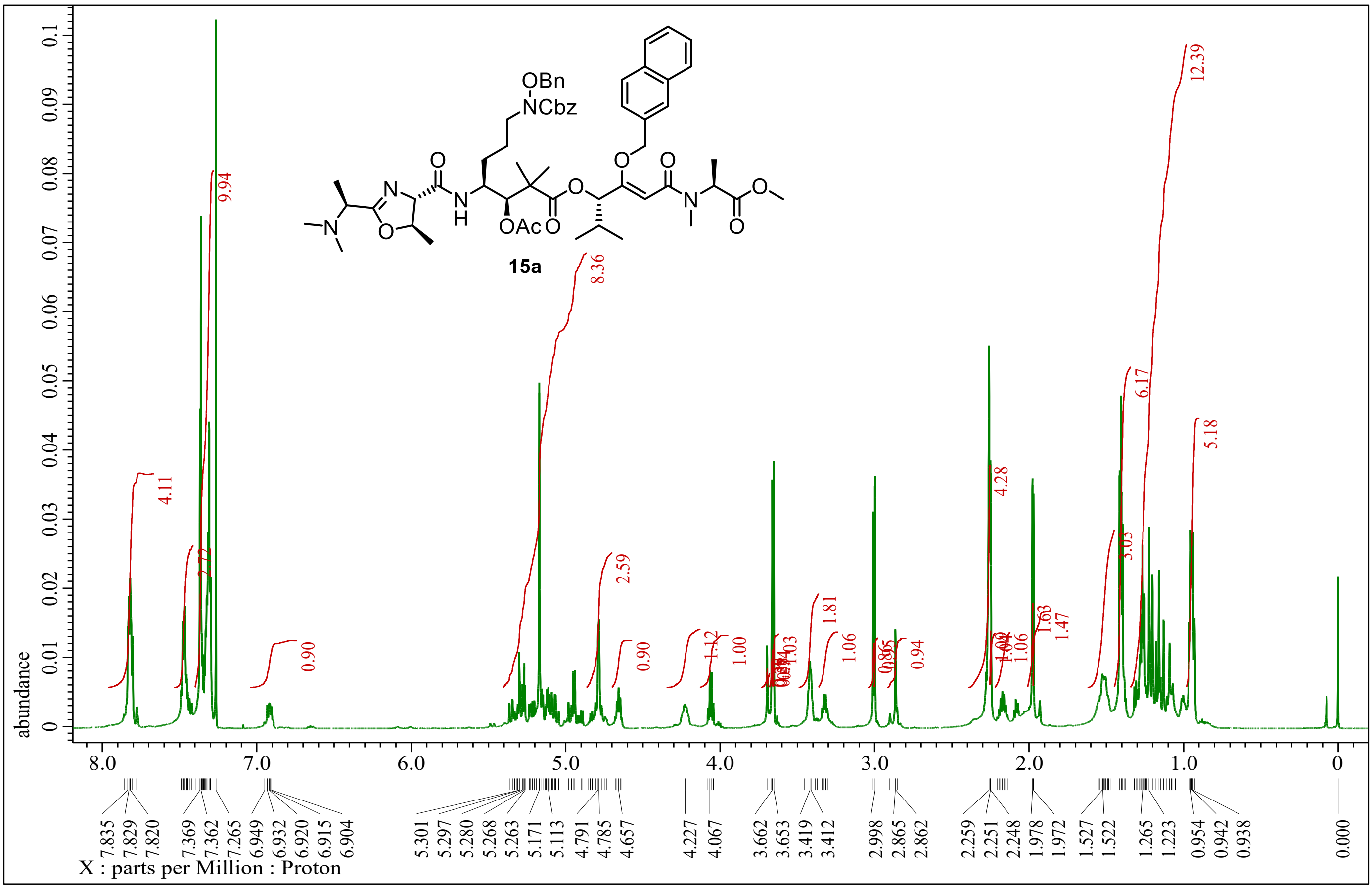

^{13}C NMR spectrum of compound **15a** (125 MHz, $CDCl_3$)

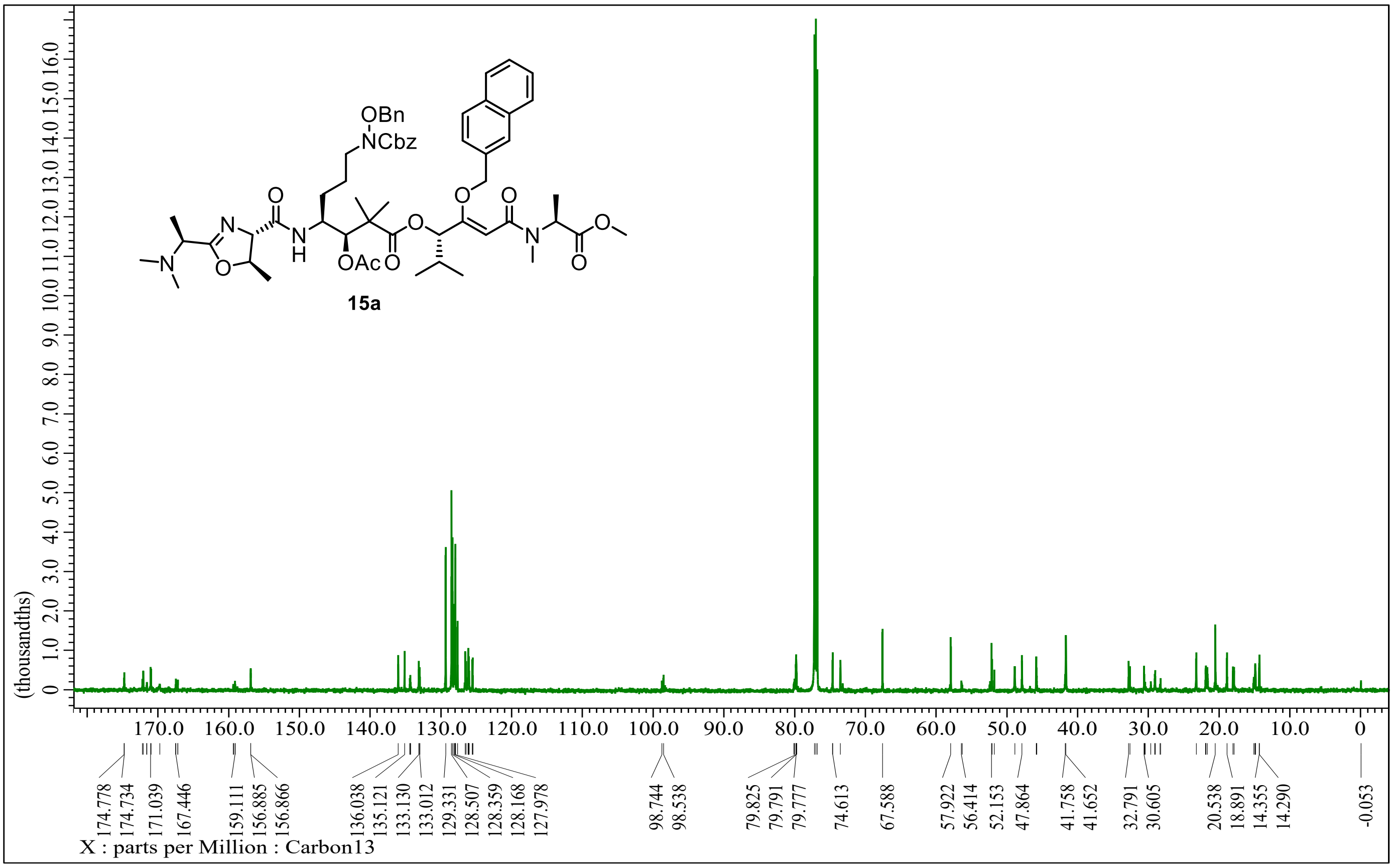

^{1}H NMR spectrum of compound **1a** (600 MHz, CD_3OD)

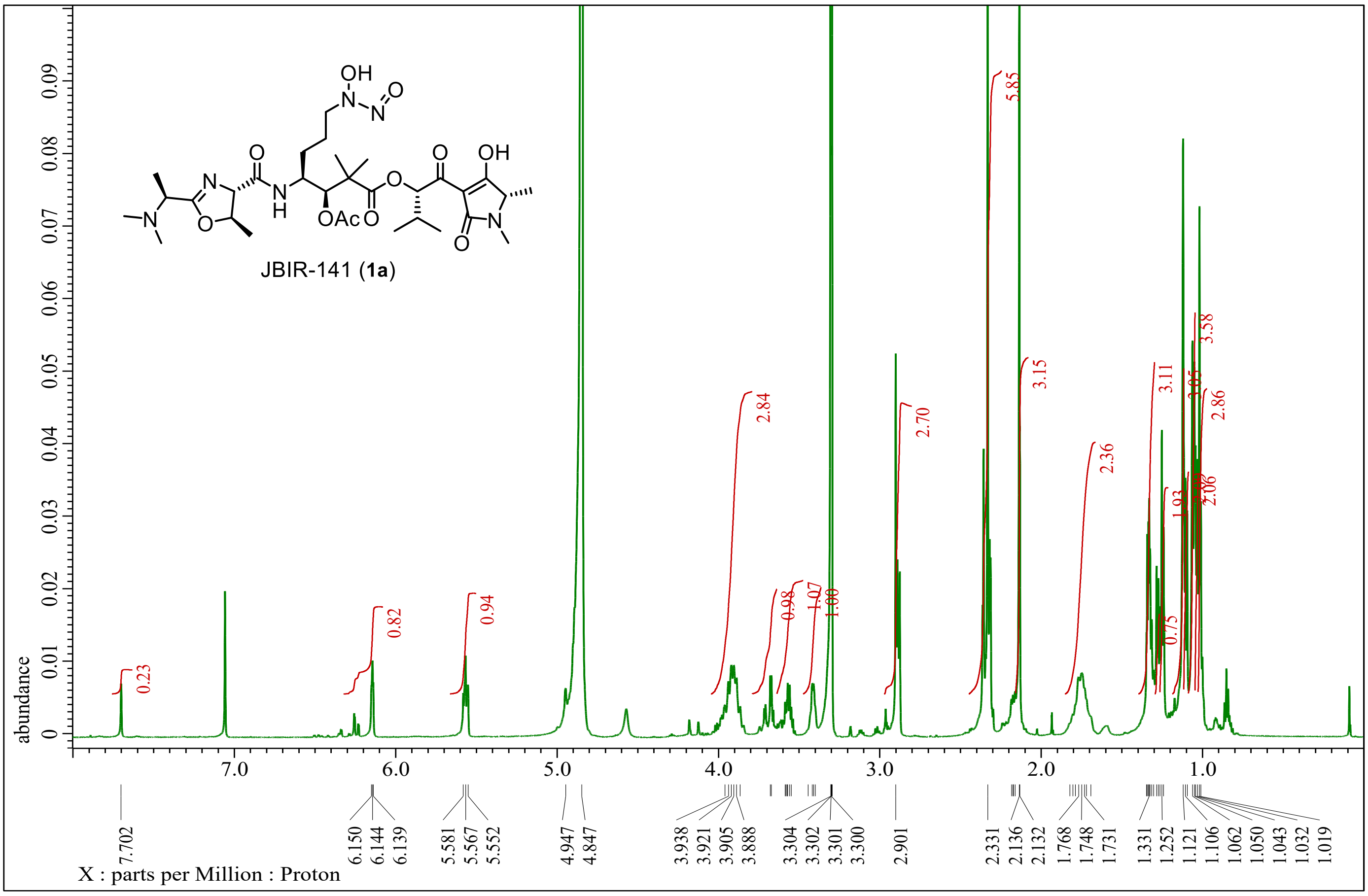

^{13}C NMR spectrum of compound **1a** (125 MHz, CD_3OD)

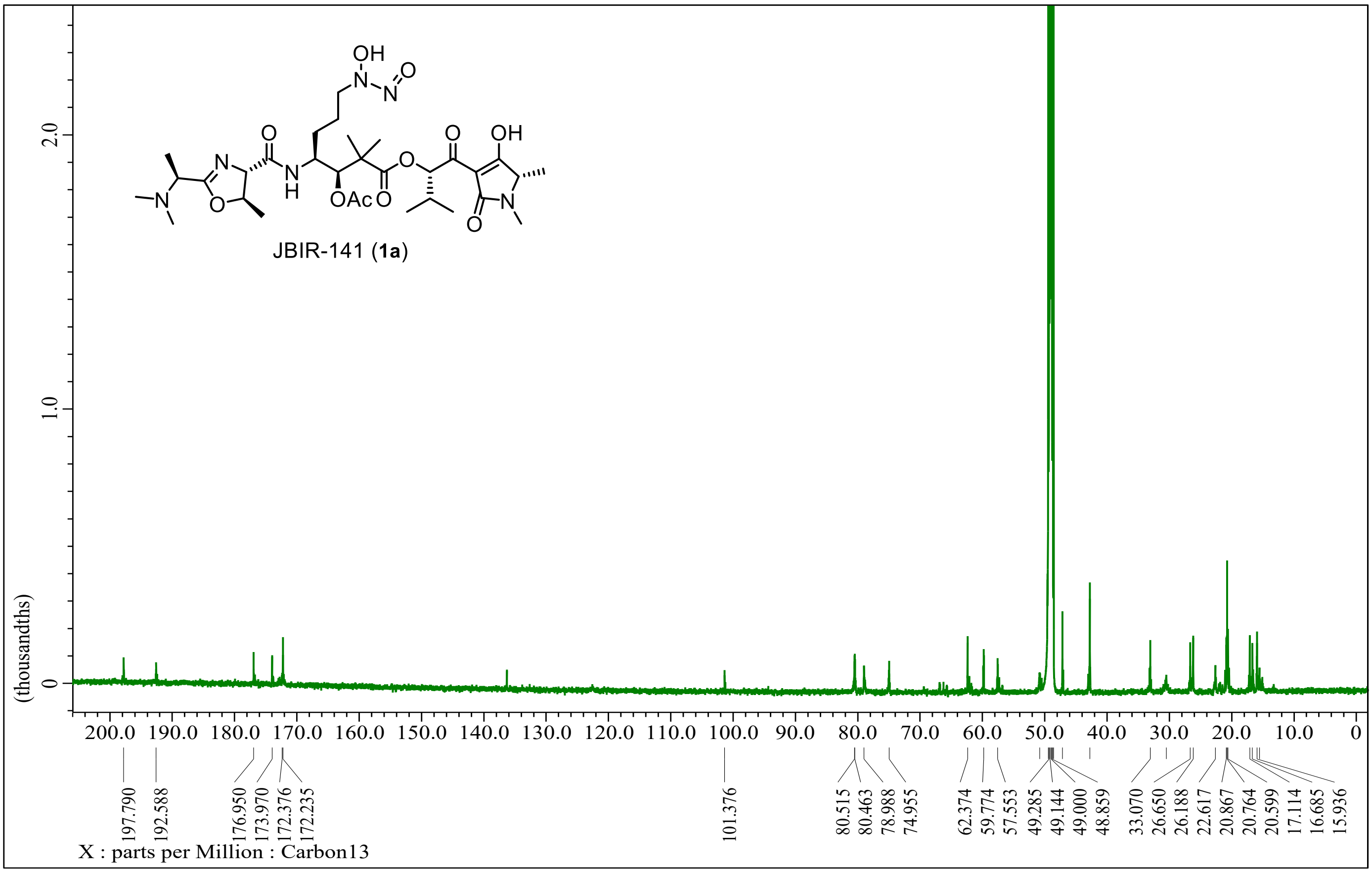

^{1}H NMR spectrum of compound **15b** (600 MHz, $CDCl_3$)

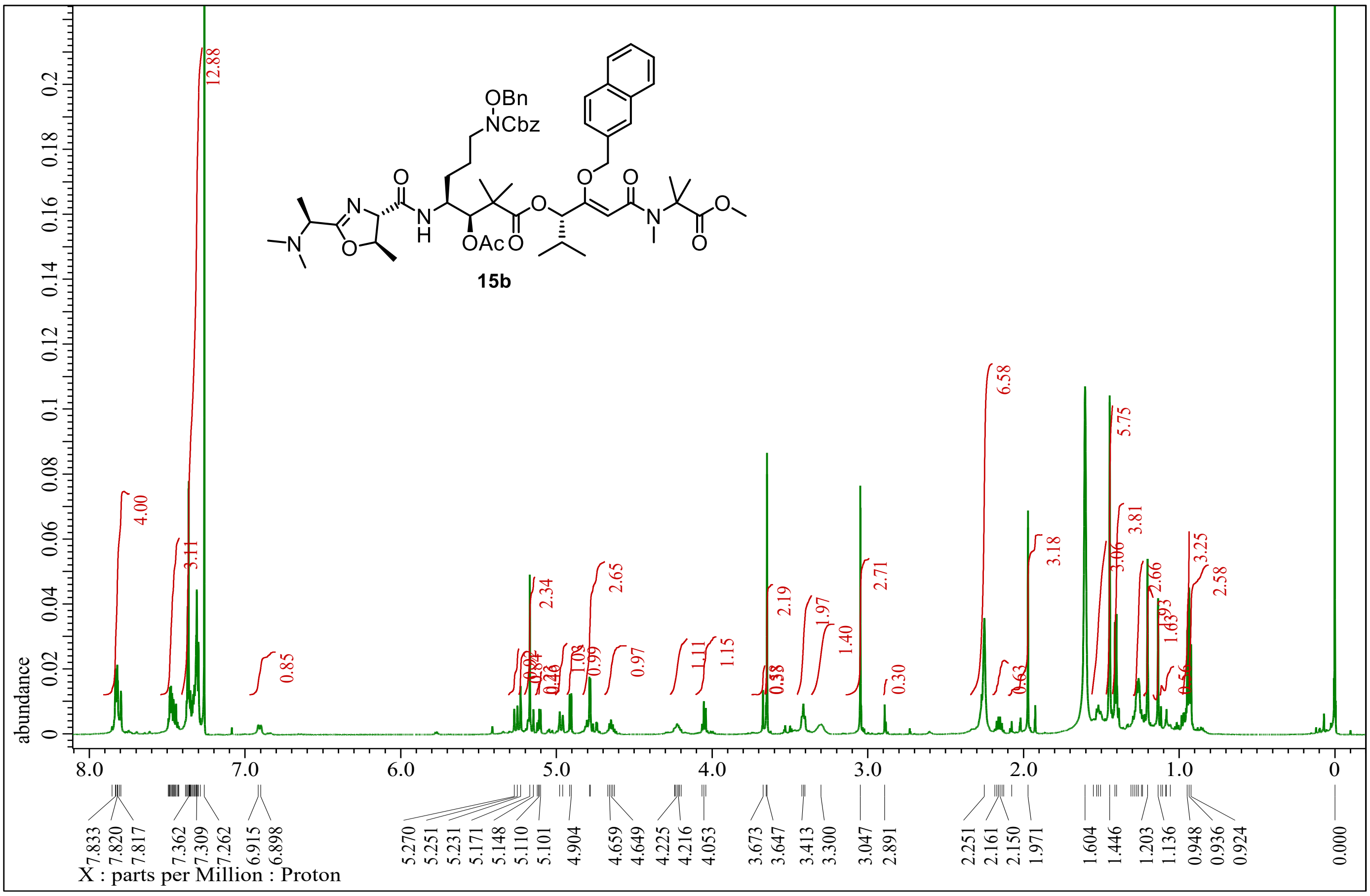

^{13}C NMR spectrum of compound **15b** (125 MHz, $CDCl_3$)

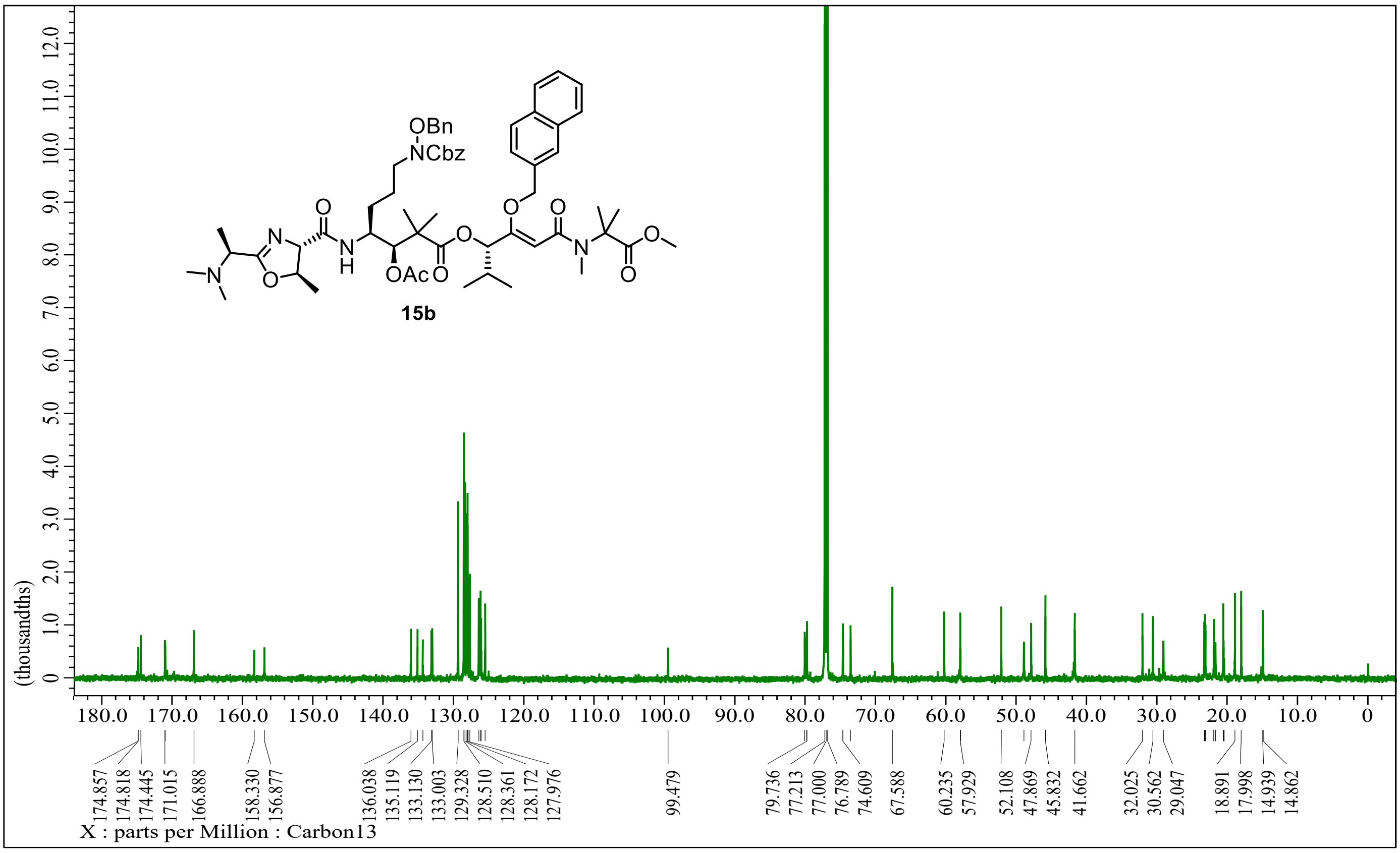

^{1}H NMR spectrum of compound **1b** (600 MHz, CD_3OD)

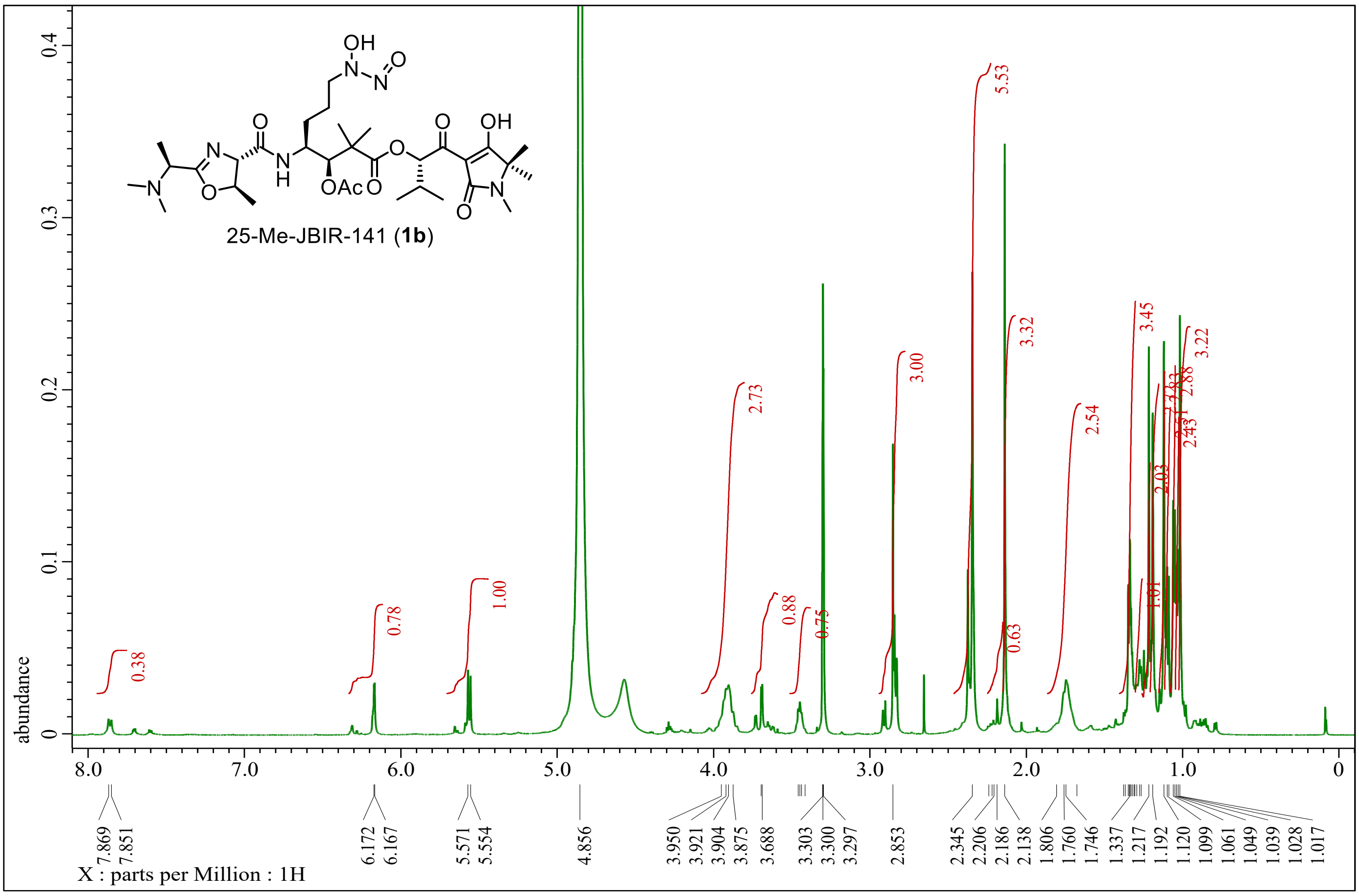

^{13}C NMR spectrum of compound **1b** (125 MHz, CD_3OD)

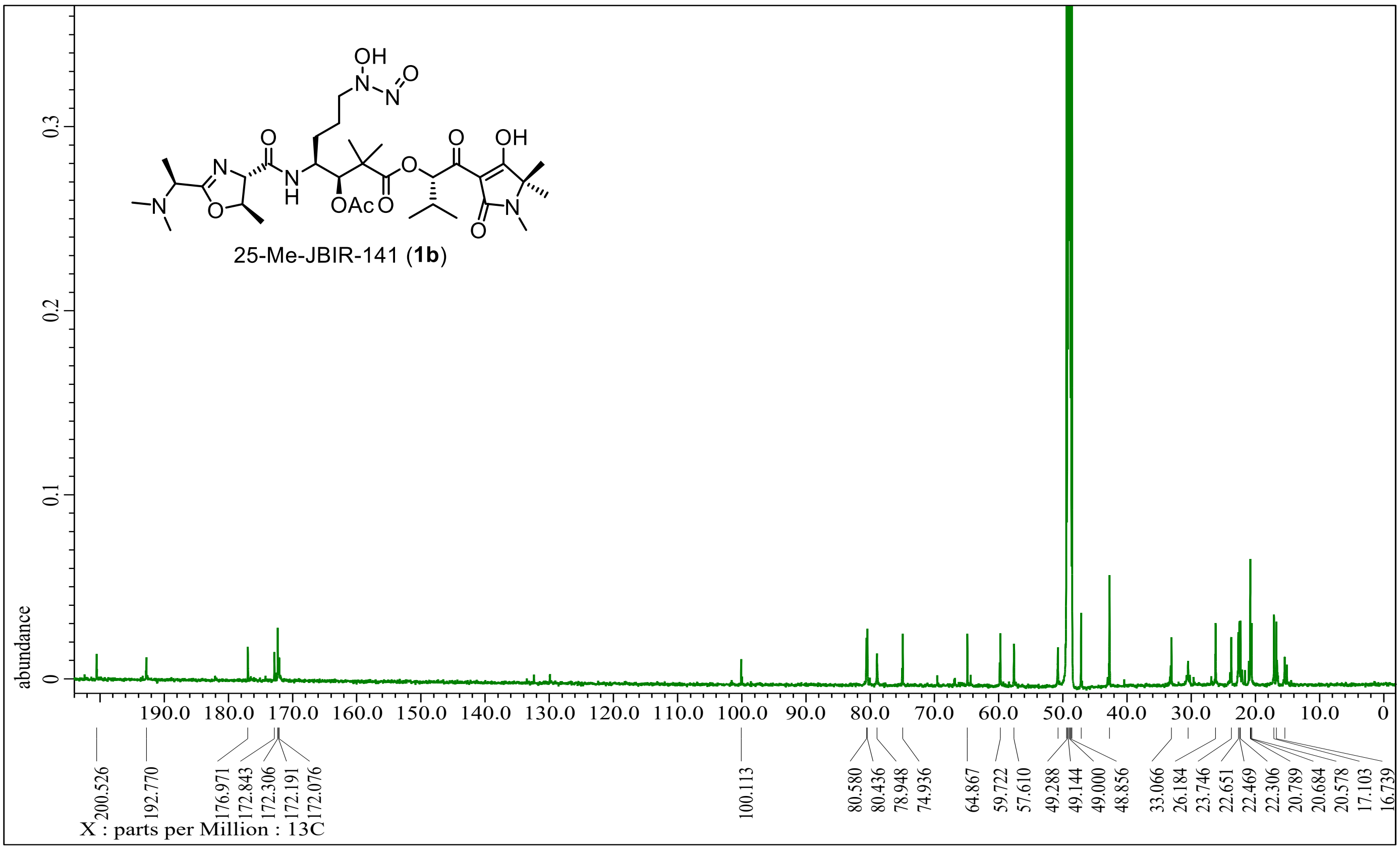

^{1}H NMR spectrum of compound **15c** (600 MHz, $CDCl_3$)

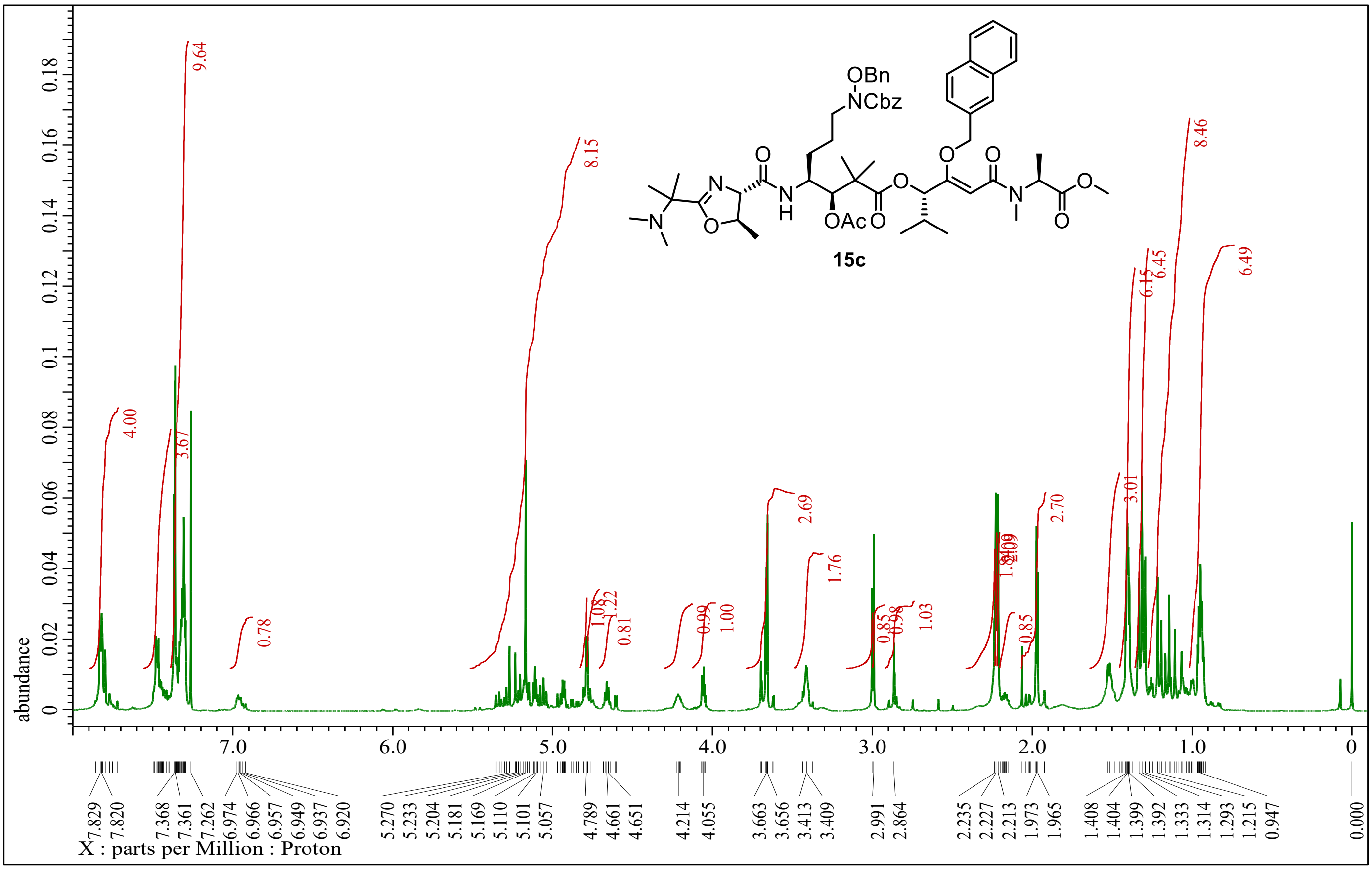

^{13}C NMR spectrum of compound **15c** (125 MHz, $CDCl_3$)

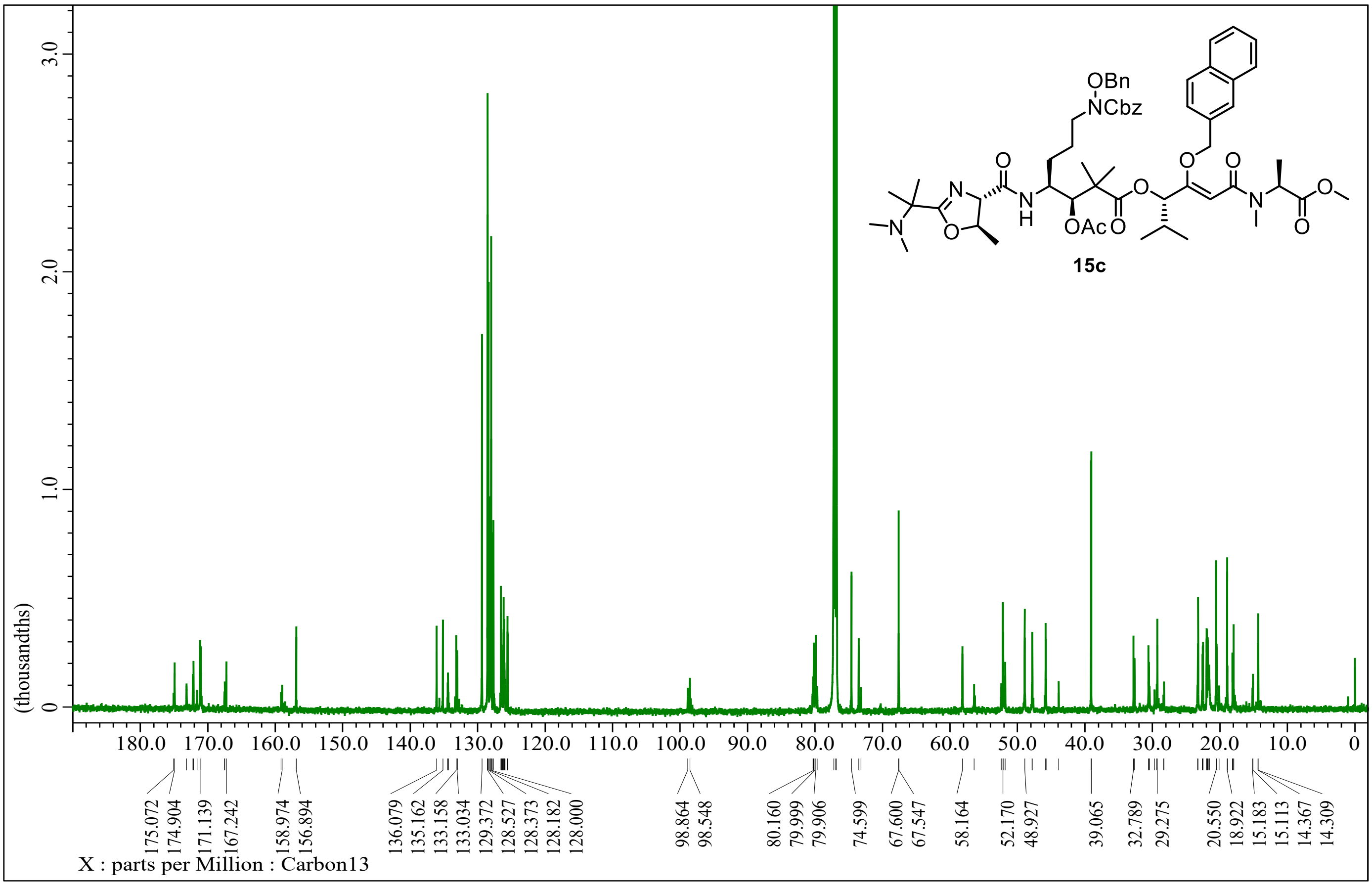

^{1}H NMR spectrum of compound **1c** (600 MHz, CD_3OD)

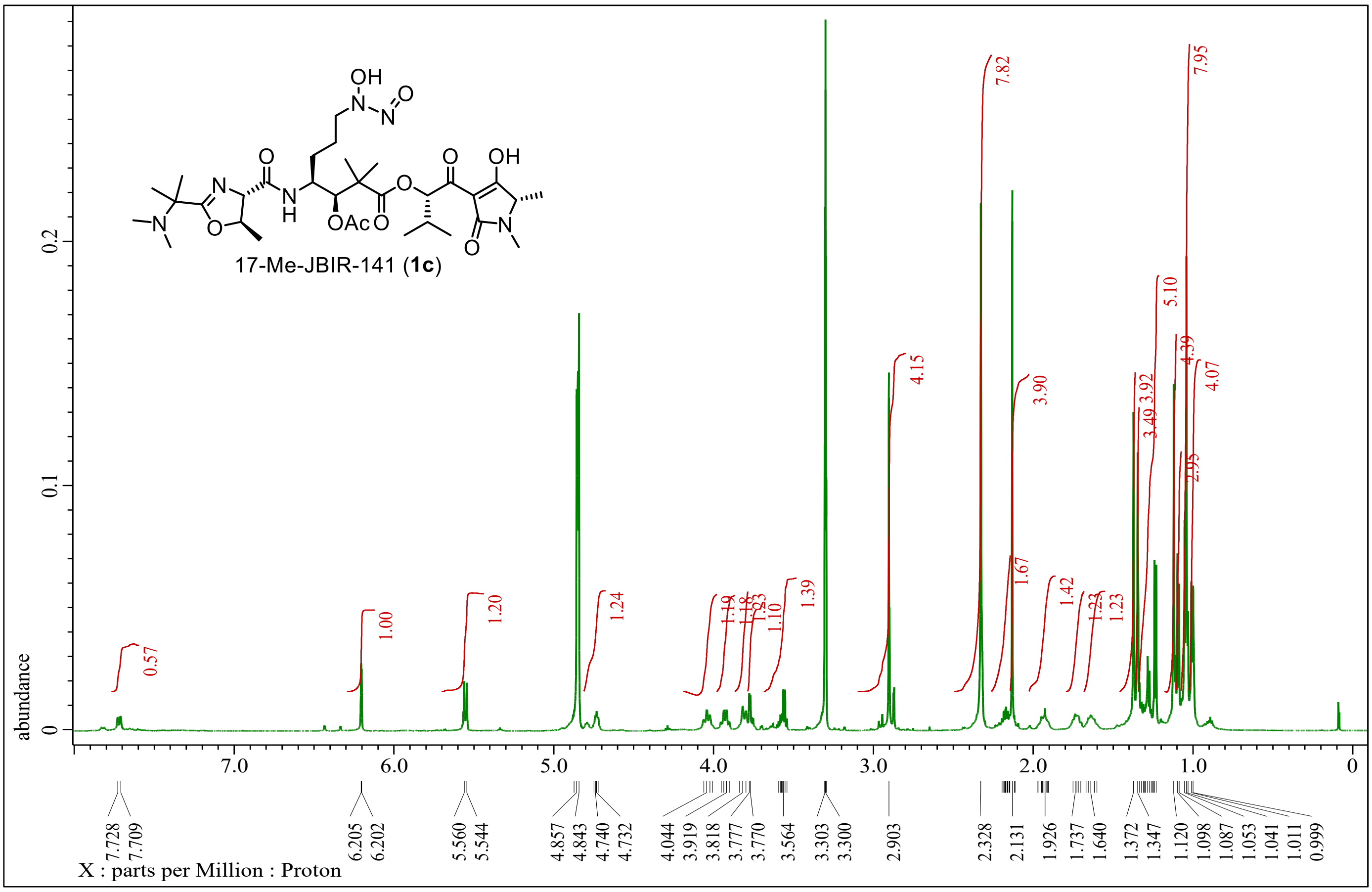

^{13}C NMR spectrum of compound **1c** (125 MHz, CD_3OD)

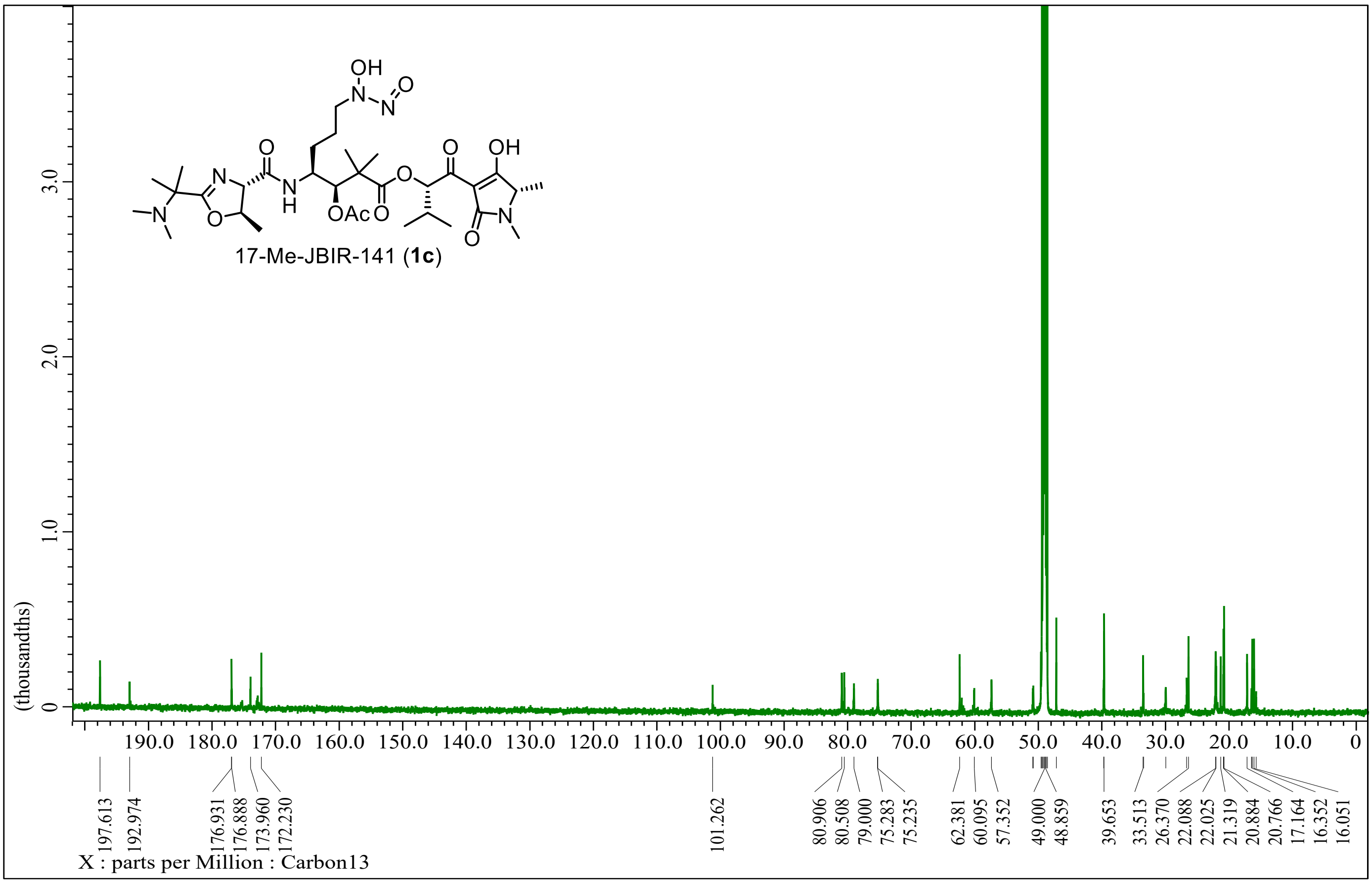

Supplement: Supplementary file 1 [file ol6c02149_si_001.pdf]
